# Pyrimidine metabolism regulator-mediated molecular subtypes display tumor microenvironmental hallmarks and assist precision treatment in bladder cancer

**DOI:** 10.3389/fonc.2023.1102518

**Published:** 2023-08-17

**Authors:** Zixuan Wu, Xiaohuan Li, Zhenchang Gu, Xinhua Xia, Jing Yang

**Affiliations:** ^1^ School of Pharmacy, Hunan University of Chinese Medicine, Changsha, China; ^2^ The Second Affiliated Hospital of Guangzhou University of Chinese Medicine, Guangzhou, China

**Keywords:** BLCA, PyMGs, immunity, m 6 A and immune checkpoint, drug prediction, CNV, SNP

## Abstract

**Background:**

Bladder cancer (BLCA) is a common urinary system malignancy with a significant morbidity and death rate worldwide. Non-muscle invasive BLCA accounts for over 75% of all BLCA cases. The imbalance of tumor metabolic pathways is associated with tumor formation and proliferation. Pyrimidine metabolism (PyM) is a complex enzyme network that incorporates nucleoside salvage, *de novo* nucleotide synthesis, and catalytic pyrimidine degradation. Metabolic reprogramming is linked to clinical prognosis in several types of cancer. However, the role of pyrimidine metabolism Genes (PyMGs) in the BLCA-fighting process remains poorly understood.

**Methods:**

Predictive PyMGs were quantified in BLCA samples from the TCGA and GEO datasets. TCGA and GEO provided information on stemness indices (mRNAsi), gene mutations, CNV, TMB, and corresponding clinical features. The prediction model was built using Lasso regression. Co-expression analysis was conducted to investigate the relationship between gene expression and PyM.

**Results:**

PyMGs were overexpressed in the high-risk sample in the absence of other clinical symptoms, demonstrating their predictive potential for BLCA outcome. Immunological and tumor-related pathways were identified in the high-risk group by GSWA. Immune function and m6a gene expression varied significantly between the risk groups. In BLCA patients, DSG1, C6orf15, SOST, SPRR2A, SERPINB7, MYBPH, and KRT1 may participate in the oncology process. Immunological function and m6a gene expression differed significantly between the two groups. The prognostic model, CNVs, single nucleotide polymorphism (SNP), and drug sensitivity all showed significant gene connections.

**Conclusions:**

BLCA-associated PyMGs are available to provide guidance in the prognostic and immunological setting and give evidence for the formulation of PyM-related molecularly targeted treatments. PyMGs and their interactions with immune cells in BLCA may serve as therapeutic targets.

## 1 Introduction

Bladder cancer (BLCA) is a common tumor worldwide with a significant morbidity and death rate (1). Over 500,000 new BLCA cases and 200,000 BLCA-related death have been reported each year globally (2, 3). The disease is classified into muscle-invasive BLCA and non-muscle-invasive BLCA. Despite a good 5-year survival rate of 90% observed in the non-muscle type, research has reported a risk of disease progression in 15-20% of BLCA patients, resulting in a compromised overall survival rate by at least 60% (4). Surgery and postoperative chemotherapy are mainstays of treatment for BLCA (5), whereas patient prognosis remains poor due to postoperative relapse despite radical surgical removal with curative intent (6).Muscle invasive or advanced bladder cancer is typically managed by chemotherapy. However, long-term chemotherapeutic medication may cause drug resistance, leading to tumor recurrence, disease progression, and increased mortality (7).

Metabolic reprogramming is a characteristic of cancer that promotes tumor cell proliferation and survival. Research has demonstrated that nucleotide metabolism influences tumor growth *via* sugar, lipid, and amino acid metabolism (8). Nucleotide metabolism involves the participation of several enzymes, including catalytic and rate-limiting enzymes such as lyase, synthase, amidotransferase, and dehydrogenase (9). PyM is a multifunctional enzyme network that performs nucleoside salvage, *de novo* nucleotide synthesis, and catalytic pyrimidine degradation. Cancer cells, unlike resting cells, rely on the *de novo* method to keep a stable supply of deoxyribonucleoside triphosphates, resulting in their uncontrolled proliferation (10). The purinosome is also a promising anti-cancer target. When used with the anti-folate methotrexate, small molecule inhibitors that disrupt purinosomes yielded a synergistic impact (11). Although more investigations are needed to confirm this protein structure as a viable target, direct control of purinosome formation may provide an exclusive means to precisely manage both the temporal and spatial accumulation of purines in cells (12). Similar to purine biosynthesis, pyrimidine nucleotide biosynthesis has both salvage and *de novo* processes, and the chemistry of both is well conserved (13). In cancer therapy, pyrimidine metabolism and purine metabolism are closely linked. Despite an association of PyM with leukemic cell differentiation, its role in solid tumor differentiation is unknown. PyM genes are connected to epithelial-to-mesenchymal transition, which is a genetic and molecular pathway associated with the loss of morphological and/or functional epithelial-like phenotypes in solid carcinomas. It causes greater resistance to treatment and improved stem-like cancer cell characteristics. These findings suggest an unique cancer therapy approach that targets purinosome development and PyM.

**Figure 1:**
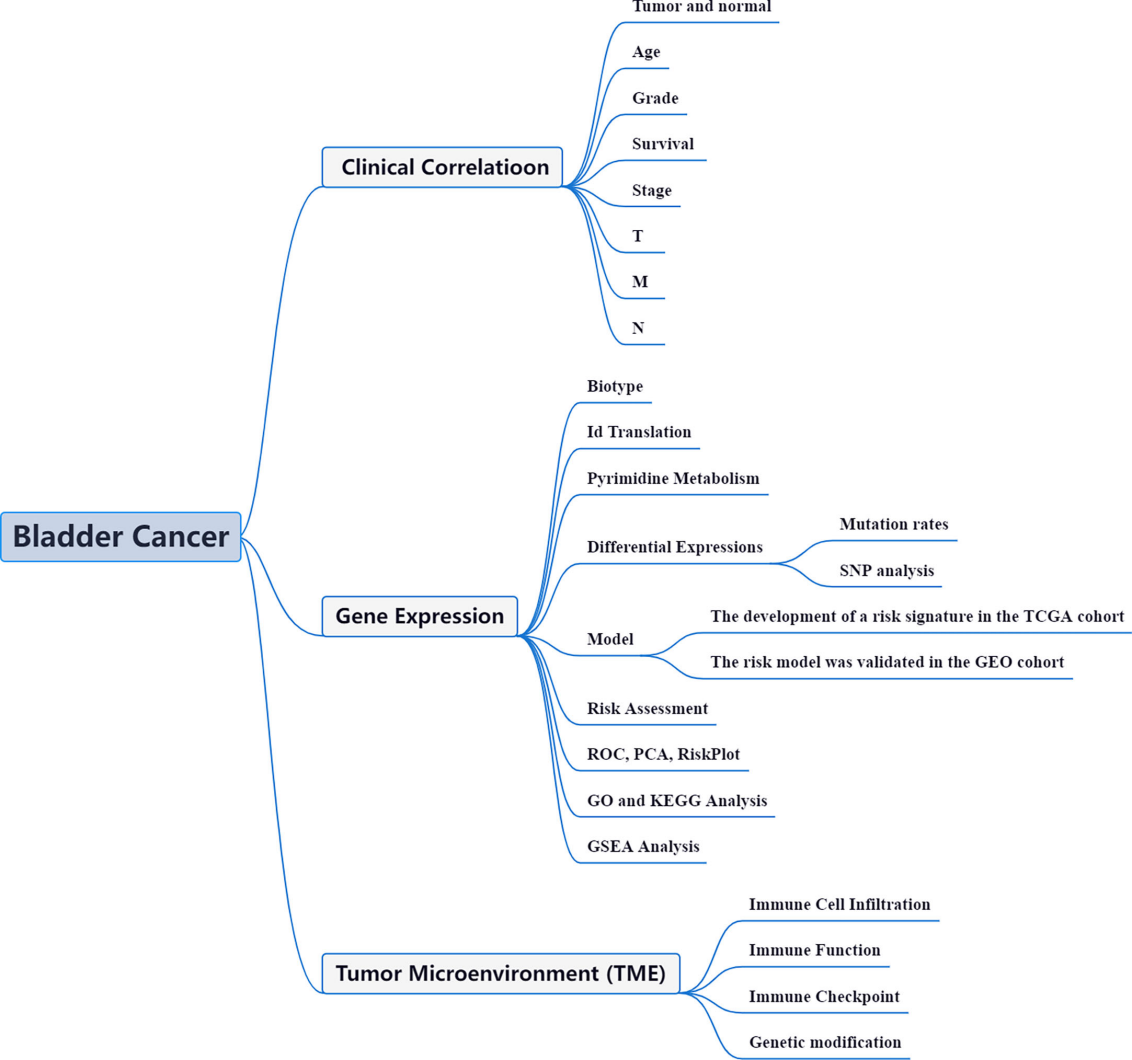
Framework based on an integration strategy of PyMGs. The data of BLCA patients were obtained from TCGA and GEO databases, and then the PyMGs were matched to carry out difference analysis and risk model construction, respectively. TCGA data set was used as the main body and GEO data were used to verify the model with good grouping, and PyMGs related to the prognosis of BLCA patients were obtained. Then, GO, KEGG and GSEA analyses were performed with multiple databases to obtain the functions related to PyMGs. Last, the immune cells, function and RNA changes were analyzed.

Tumor microenvironments (TME) are characterized by hypoxia, excessive oxidation, acidity, and malnutrition as a result of tumor cell proliferation and insufficient angiogenesis (14). Thus, cancer cells have metabolic properties that distinguish them from normal cells in order to protect the growth and survival of cancer cells through a process of metabolic reprogramming to address hazardous TMEs when carcinogenic signals are silenced. Energy metabolism reprogramming is essential for cancer cell proliferation and division (15). The utilization methods of nutrients differ between cancer cells and normal cells. It has been suggested that PyM has an impact on oncogenesis and cancer metastasis. There are 172 different types of RNA changesidentified, and M6A, m1A, M7G, and m5C are the most prevalent chemical alterations. One of the most common eukaryotic mRNA modifications is m6A (16). Immune checkpoint inhibitor (ICI) profiles in BLCA patients may aid in diagnosing, analyzing, and anticipating therapy results (17). The reason and methods of BLCA’s aberrant gene expression and PyM are yet poorly understood. Hence, exploration of regulation of BLCA synthesis *via* Pym may contribute to identifying effective biomarkers. The framework of the current investigation is depicted in **Figure 1**.

## 2 Materials and methods

The research methodology adopted herein was developed with reference to that of Zixuan Wu et al., 2022 (17).

### 2.1 Datasets and PyMGs

BLCA gene expression patterns and clinical data were obtained from TCGA (18). Microdata on mRNA expression was found using GEO.

### 2.2 DEGs and mutation rates

To obtain precise mRNA data, transcription data were matched and sorted using Perl. The IDs were then translated into gene names. To assess a substantial change in PyM genes expression, FDR<0.05 and |log2FC|≥1 were adopted. The relevance of differentially expressed PyMGs was investigated (DEGs). Cbioportal was used to estimate DEG variant frequencies.

### 2.3 Tumor classification using the DEGs

We performed cluster analysis using the Limma and ConsensusClusterPlus packages and divided the prognosis-related PyMGs into two clusters: cluster 1 and cluster 2. Survminer was used to investigate PyMG survivorship, and survival was utilized to assess PyMG predictive usefulness. The limma was utilized to identify alterations in particular genes between subtypes and tissue types.

### 2.4 Cluster DEGs

To assess a substantial change in PyMGs Cluster DEGs expression, FDR<0.05 and |log2FC|≥1 were utilized. These genes are then visualised in a heatmap.

### 2.5 PyMGs prognostic signature

DEGs were divided into two categories: a low-risk group and a high-risk group. Lasso regression was connected to two types of risk. The survival curves for the two categories were compared. To assess the model’s accuracy in predicting survival in BLCA, the timeROC was used to generate a ROC curve. PyMGs hazard and survival status were investigated for the risk score’s probability curve. A nurse-independent prediction study was conducted to determine the effect of the model on clinical factors. A link was identified between two populations ofPyMGs. Risk and clinical interaction studies are available. T-SNE and PCA techniques were also studied. To understand if the prognostic model was successful in separating patients into two groups, the desegregation of prognosticative markers was utilized to build a representation that predicts BLCA patients’ 1-, 3-, and 5-year OS.

### 2.6 GO and KEGG analysis

Using GO and KEGG, the biological function and pathways linked with the DEGs were investigated. BP, MF, and CC regulated by differentially expressed PyMGs were explored using R.

### 2.7 GSEA enrichment analyses

In a range of samples, GSEA was employed to identify related functions and route alterations. The accompanying score and diagrams were used to determine that activities and pathways within the various risk subcategories were dynamic. Each sample was labeled ‘H’ or ‘L’.

### 2.8 The levels of immune activation in different segments

The assessment of ssGSEA was employed. We assessed the enriching values of immune cells and activities and also examined the connection between PyMGs, checkpoints, and mRNA chemical modifications (m6A, m1A, M7G, and m5C). m6A, m1A, M7G, and m5C regulators were also identified (19) (**Table S2**).

## 3 Results

### 3.1 Datasets and GlnMgs

The data of 412 BLCA and 19 normal tissues were enrolled in the TCGA on October 28, 2022. Series: GSE13507, GSE48075, and GSE48276. Platform: GPL6102, GPL6947, and GPL14951. The GEO shared database was used to maintain the expression patterns of 307 BLCA cases (**Table 1**). A total of 105 PyMGs were obtained (**Table S1**).

**Table 1:** The clinical characteristics of patients.

TCGA		GEO (GSE13507, GSE48075, and GSE48276)	
Variables	Number of samples	Variables	Number of samples
Gender		Gender	
Male/Female	304/108	Male/Female	96/211
Age at diagnosis		Age at diagnosis	
≤65/>65	162/250	≤65/>65	127/182
Grade		Grade	
High/Low/NA	388/21/3	High/Low	60/105
Stage		Stage	
I/II/III/IV/NA	2/131/141/136/2	I/II/III/IV/NA	Unknow
T		T	
T1/T2/T3/T4/NA	3/120/196/59/34	T1/T2/T3/T4/NA	90/134/85/27/26
M		M	
M0/M1/NA	196/11/205	M0/M1/NA	337/9/6
N		N	
N0/N1/N2/N3/NA	239/47/76/8/42	N0/N1/N2/N3/NA	323/11/7/1/10

### 3.2 Differentially expressed PyMGs

**Figure 2:**
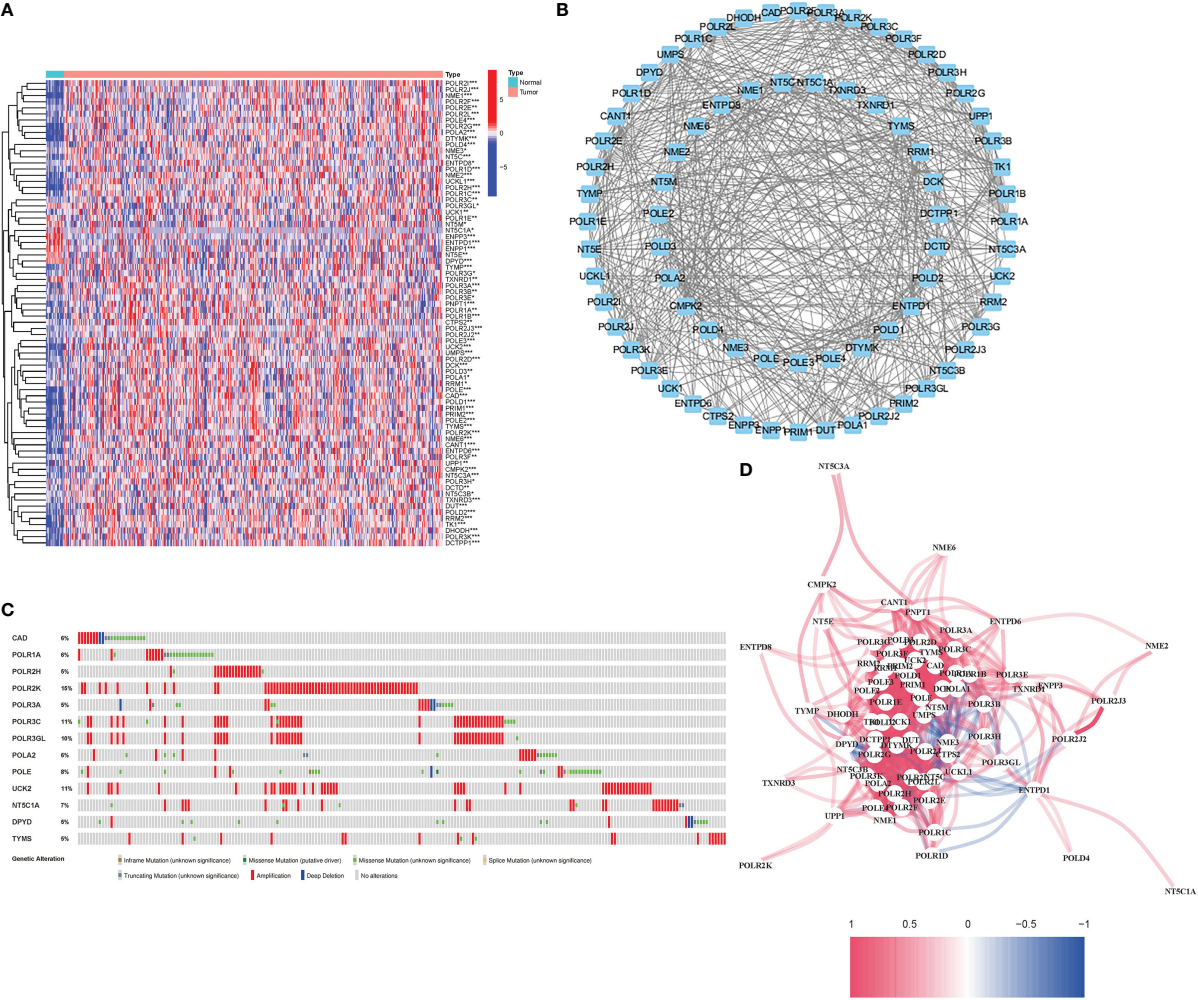
Expressions of the 76 PyMGs and their interactions **(A)** A PPI network illustrating the interactions of PyMGs (interaction score=0.7). **(B)**: The PyMGs correlation network (red line: positive correlation; blue line: negative correlation). **(C)** Mutations in PyMGs. 13 genes over a 5% mutation rate, with POLR2K being the most often modified (15%). **(D)** The correlation network of the PyMGs (red line: positive correlation; blue line: negative correlation. The depth of the colors reflects the strength of the relevance).

76 DEGs demonstrated a close association with PyM (67 upregulated, 9 downregulated; **Table S2**) (**Figure 2**). A protein-protein interaction (PPI) network was established to evaluate the interactions of PyMGs, as shown in **Figure 2**. By lowering the low required interaction value to 0.7, CTPS2, POLR1B, UMPS, RRM1, POLR1C, DHODH, and POLR1A were determined as hub genes (**Table S3**). These genes, which comprised all DEGs discovered in both normal and malignant tissues, showed predictive value for BLCA. **Figure 2** depicts a correlation network of all PyMGs. Genetic anomalies in these PyMGs were further investigated.The most common types of mutations were truncating and missense variants(**Figure 2**). A total of 13 genes mutated at a rate of more than 5%, with POLR2K commonly altered (15%).

### 3.3 Alterations of PyM regulatory genes are associated with clinicopathological and molecular characteristics

**Figure 3:**
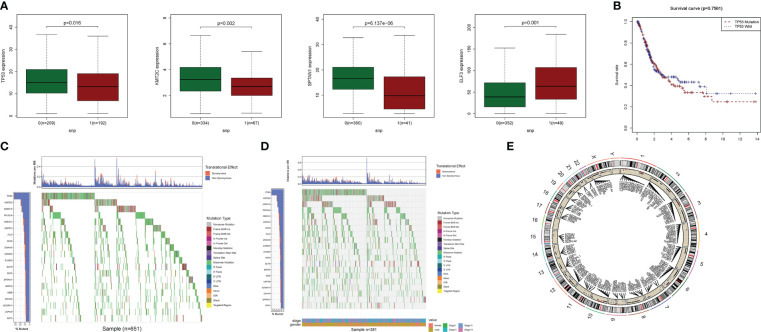
CNV, SNP and mutation analysis.**(A)** Correlation study of gene expression in prognostic signatures and SNP. **(B)** The survival analysis of TP53. **(C, D)**: The mutation distribution of genes in prognostic signatures. **(E)** CNV analysis.

The relationship between alterations in PyM regulatory genes (CNV, SNP, and mutation) and clinicopathological parameters of patients were assessed. Correlation analysis of DEG expression in the prognostic model and SNP revealed four SNP-driven DEGs: TP53, ELF3, KMT2C, and SPTAN1 (**Figure 3**). TP53 exhibited a significantly higher expression level in the single mutations group than in the non-mutations group (P<0.05), showing that SNP in BLCA may cause dysregulation of critical genes. A waterfall plot was employed to display the status of gene mutations. In the prognostic model, the total average mutation frequency of DEGs varied from 12 to 50% (**Figures 3B, C**), suggesting that BLCA mutations may be associated with the deregulation of critical genes. Correlation examination of DEG expression in the prognostic model and CNV revealed several CNV-driven DEGs (**Figure 3D**).

The model’s medication prediction revealed certain genes with significant differences (**Figure S1**). Furthermore, an investigation of the connection between DEG expression in the prognostic model indicated that numerous genes were associated with drug sensitivity. MYBPH is strongly linked to TESTOLACTONE, Procarbazine, Olaparib, and Simvastatin, indicating potential medication pathways (**Figure S2**).

### 3.4 Tumor categorization using the DEGs

**Figure 4:**
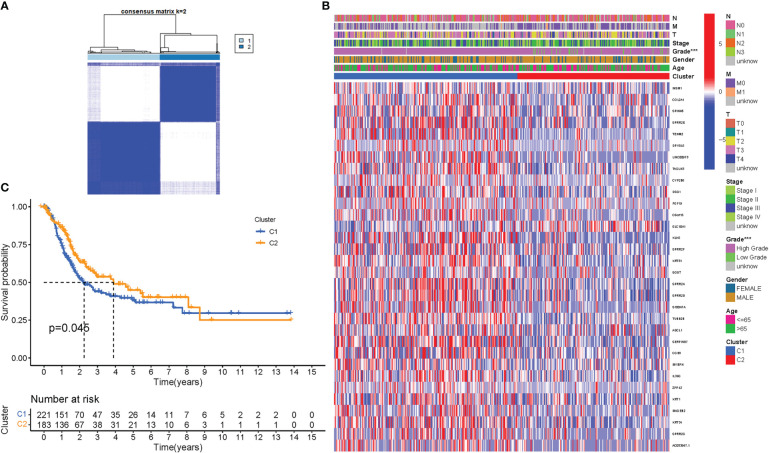
Tumor categorization based on DEGs associated with PyM. **(A)** The consensus clustering matrix (k=2) was used to divide 414 BLCA patients into two groups. Heatmap **(B)**. The heatmap and clinicopathologic features of the two clusters identified by these DEGs (T, Grade, and Stage indicate the degree of tumor differentiation. **(C)** Kaplan-Meier OS curves for the two clusters.

To assess the associations between PyMGs expression and BLCA, a consensus clustering analysis was performed on all 414 BLCA patients in the TCGA dataset. With the clustering variable (k) at 2, a strongest intragroup correlation and a weakest intergroup correlation were observed, showing that the 414 BLCA patients could be classified into two groups based on their PyMGs (**Figure 4A**). A heatmap depicts gene expression and clinical features (**Figure 4B**, **Table S4**). Survival research was carried out to investigate PyMGs’ predictive potential utilizing PyMGs subtypes, and cluster 2 had a better survival rate (P=0.045), **Figure 4C**.

### 3.5 In the TCGA cohort, a prognostic gene model was developed

**Figure 5:**
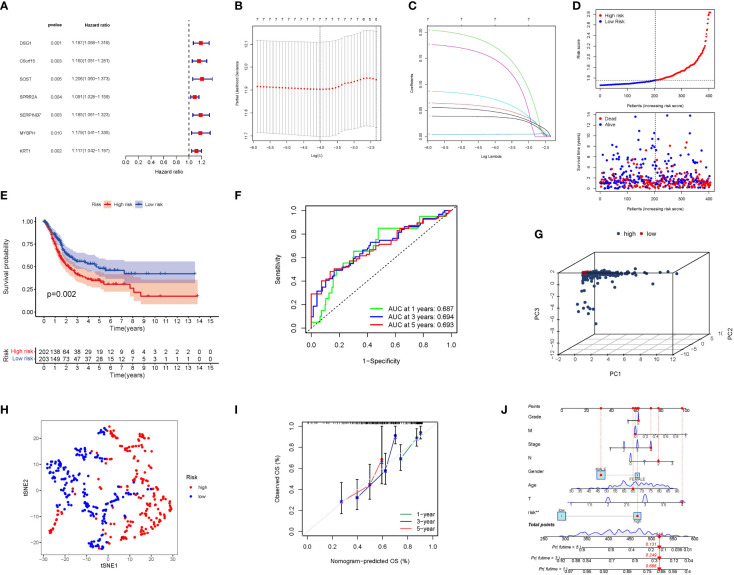
The development of a risk signature in the TCGA cohort. **(A)** A Univariate Cox regression analysis of OS for each PyMGs, with P<0.05 for 7 genes. **(B)** Regression of OS-related genes. **(C)**: Cross-validation is used in the LASSO regression to fine-tune parameter selection. **(D)** The patient’s chance of survival. **(E)** Kaplan-Meier curves. **(F)** The AUC for predicting the 1-, 3-, and 5-year survival rates. **(G)** A PCA plot. **(H)** A t-SNE plot. **(J, K)** Nomogram.

The univariate Cox study identified 7 significant PyMGs. These PyMGs (DSG1, C6orf15, SOST, SPRR2A, SERPINB7, MYBPH, and KRT1) were considered independent BLCA prognostic indicators (**Figure 5A**). The absolute minimal shrinkage and selection operator (LASSO), Cox regression analysis, and optimum value were used to establish a gene signature (**Figures 5B, C**). The risk scores of patients were negatively related to BLCA survival. The majority of the novel PyMGs found had a negative link with the risk model, requiring further investigations (**Figure 5D**). The presence of high-risk PyMGs signatures was associated with a lower likelihood of survival (P=0.002, **Figure 5E**). The AUC predictive value of the unique PyMGs signature for 1, 3, and 5-year survival rates was 0.687, 0.694, and 0.693, respectively (**Figures 5F**). Based on the PCA and t-SNE findings, patients with differing risks were separated into two groups (**Figures 5G, H**). The hybrid nomogram, including TCGA clinicopathological traits and PyMG’s prognostic signature, was stable and accurate, indicating considerable promise in the treatment of BLCA patients (**Figures 5J, K**).

### 3.6 The risk signature is validated externally

**Figure 6:**
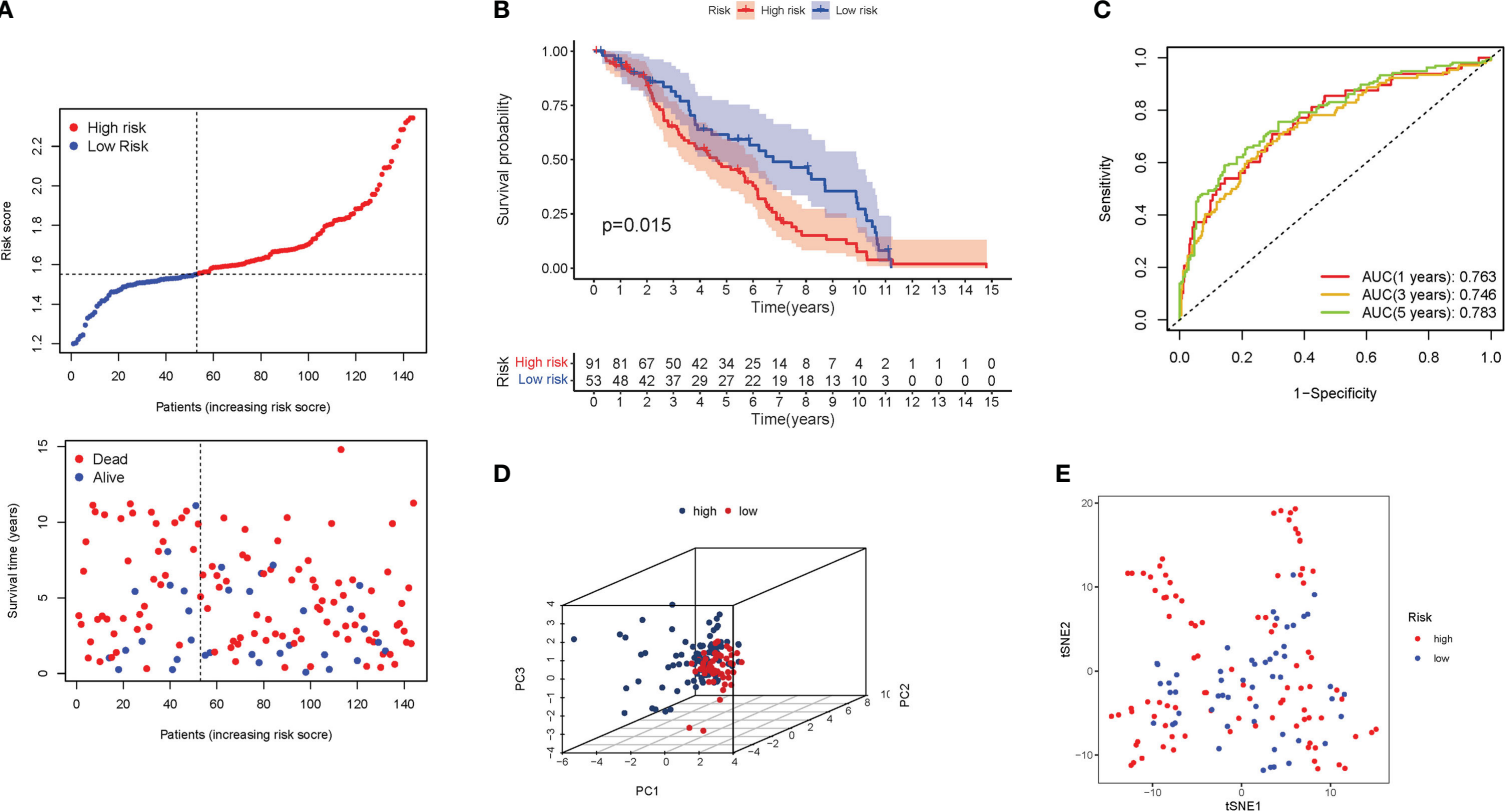
The risk model was validated in the GEO cohort. **(A)** Each patient’s chance of survival. **(B)** Kaplan-Meier curves for patients. **(C)** The AUC for predicting the 1-, 3-, and 5-year survival rates of BLCA. **(D)**: A PCA plot. **(E)**: A t-SNE plot.

The validation group was a GEO cohort of 307 BLCA patients. The risk score of patients was adversely proportional to BLCA survival. Similar to the TCGA findings, the majority of the new PyMGs found in the present study were negatively associated with the risk model (**Figure 6A**). The presence of high-risk PRG signatures indicated a compromised survival status (P=0.015). **Figure 6B** was plotted using Kaplan-Meier analysis. The AUC predictive value of the unique PyMGs signature was 0.763, 0.746, and 0.783 for 1, 3, and 5-year survival rates, respectively (**Figure 6C**). The vast majority of BLCA patients died within five years, which contributed to the lower AUC, and the PCA and t-SNE results indicated that patients with variable risks were effectively divided into two groups.(**Figures 6D, E**).

### 3.7 The risk model’s independent prognostic value

**Figure 7:**
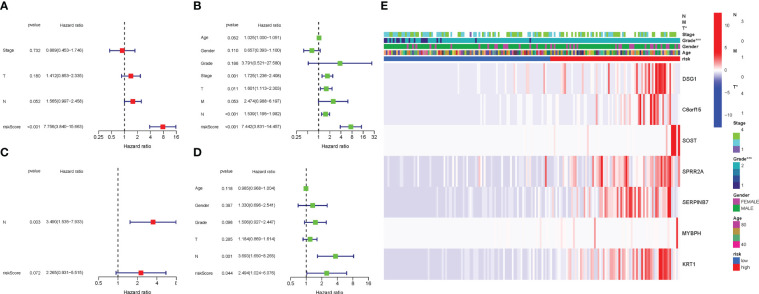
Cox regression analysis, both univariate and multivariate. **(A)** TCGA cohort multivariate analysis. **(B)**: TCGA cohort univariate analysis. **(C)**: GEO cohort multivariate analysis. **(D)**: GEO cohort univariate analysis. **(E)**: Heatmap illustrating the relationships between clinicopathologic characteristics and risk groups P values were showed as: ns not significant; *P < 0.05; **P < 0.01; ***P < 0.001.

COX analysis in the TCGA cohort revealed that the PyMGs signature (HR: 7.756, 95CI:3.840-15.663) was predominantly independent predictive factors for the OS of BLCA patients (**Figures 7A, B**). COX analysis in the GEO cohort revealed that N stage (HR: 3.490, 95CI: (1.535-7.933) was a largely independent predictive factor (**Figures 7C, D**). In addition, a heatmap of clinical features for the TCGA cohort was depicted (**Figures 7E**) (**Table S5-6**).

### 3.8 Enrichment analysis of PyMGs

**Figure 8:**
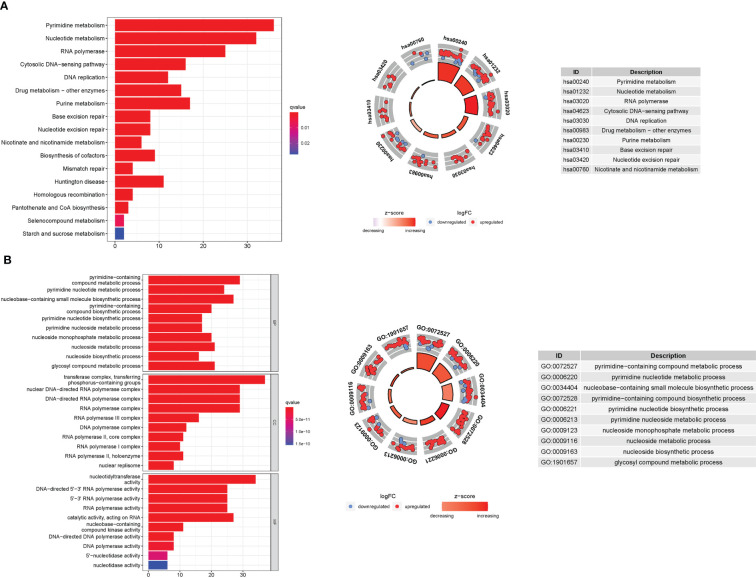
For PyMGs, GO, and KEGG analyses were performed. GO and KEGG analyses for genes participating in autophagy. **(A)**: Barplot graph for KEGG pathways (the longer bar means the more genes enriched, and the increasing depth of red means the differences were more obvious); The KEGG circle shows the scatter map of the logFC of the specified gene. The higher the Z-score value indicated, the higher expression of the enriched pathway. **(B)**: Bubble graph for GO enrichment (the bigger bubble means the more genes enriched, and the increasing depth of red means the differences were more obvious; q-value: the adjusted p-value); The GO circle shows the scatter map of the logFC of the specified gene.

GO enrichment analysis revealed 278 core targets, including BP, MF, and CC. The MF mainly involves nucleotidyltransferase activity (GO:0016779), catalytic activity, acting on RNA (GO:0140098), and DNA-directed 5’-3’ RNA polymerase activity (GO:0003899). CC mainly involves transferase complex, transferring phosphorus-containing groups (GO:0061695), nuclear DNA-directed RNA polymerase complex (GO:0055029), and DNA-directed RNA polymerase complex (GO:0000428). BP mainly involves pyrimidine-containing compound metabolic process (GO:0072527), nucleobase-containing small molecule biosynthetic process (GO:0034404), and pyrimidine nucleotide metabolic process (GO:0006220). In addition, the main signaling pathways were identified using KEGG enrichment analysis, which revealed that the over-expressed genes were involved in Pyrimidine metabolism (hsa00240), nucleotide metabolism (hsa01232), RNA polymerase (hsa03020), and pyrimidine metabolism (hsa00230). (**Figure 8** and **Table S7, 8**).

### 3.9 Analyses of gene set enrichment

**Figure 9:**
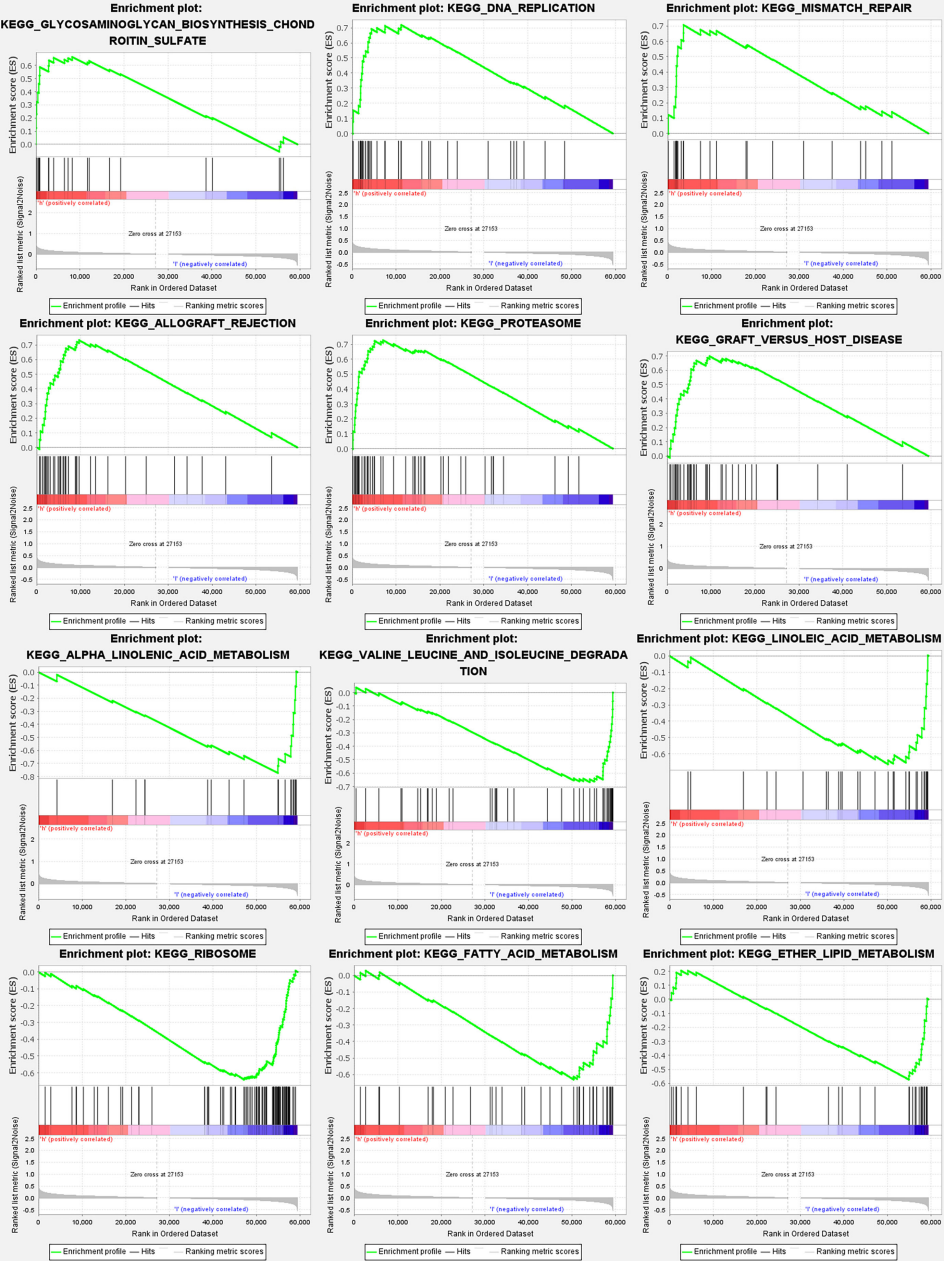
PyMGs gene set enrichment studies. The top six enriched functions or pathways of each cluster were provided to illustrate the distinction between related activities or pathways in various samples. The ‘nod-like receptor signaling pathway’ was the most enriched.

According to GSEA, most PyMGs prognostic signatures regulated immunological and tumor-related pathways such as allograft rejection, proteasome, DNA replication, alpha-linolenic acid metabolism, valine leucine, and isoleucine degradation, and linoleic acid metabolism. The top 6 enriched functions or pathways for each cluster are shown in **Figure 9** and **Table 2**. The “‘nod-like receptor signaling pathway” was the most enriched (**Tables S9A, B**).

**Table 2:** The top six enriched functions or pathways.

NAME	ES	NES	NOM p-val	FDR q-val
allograft rejection	0.7326922	1.7128558	0.044573642	0.05268823
proteasome	0.7270414	1.9887205	0.006465518	0.047390986
dna replication	0.7185649	1.8164389	0.014403292	0.04490206
alpha linolenic acid metabolism	-0.7724566	-2.2641535	0	3.60E-04
valine leucine and isoleucine degradation	-0.66707414	-2.1426907	0	0.002941816
linoleic acid metabolism	-0.6642279	-2.0364537	0	0.012096318

### 3.10 Immune activity levels in different subgroups are compared

Tumor-associated macrophages (TAMs) are an important cellular component of the tumor microenvironment (TME). Li suggested that TAMs produce considerable variability at the transcriptional, developmental, metabolic, and functional levels (20). Yulia Kushnareva used loss-of-function screening to identify genes that were specifically enriched in the Th1* subpopulation. The genetic screen identified candidates whose depletion dramatically reduced TCR-induced interferon gamma (IFN) production. These included genes previously connected to IFN or MTB susceptibility as well as new possibilities such ISOC1, which encodes a metabolic enzyme with uncertain roles in mammalian cells (21). ISOC1-depleted T cells generated less IFN and IL-17, had deficiencies in oxidative phosphorylation and glycolysis, and a pyrimidine metabolic pathway deficit. Extracellular pyrimidine supplementation restored both bioenergetics and IFN production in ISOC1-deficient T cells, demonstrating that pyrimidine metabolism is a critical driver of effector activities in CD4+ T cells and Th1* cells (22). This shows that pyrimidine metabolism is closely related to immunotherapy and infiltration.

**Figure 10:**
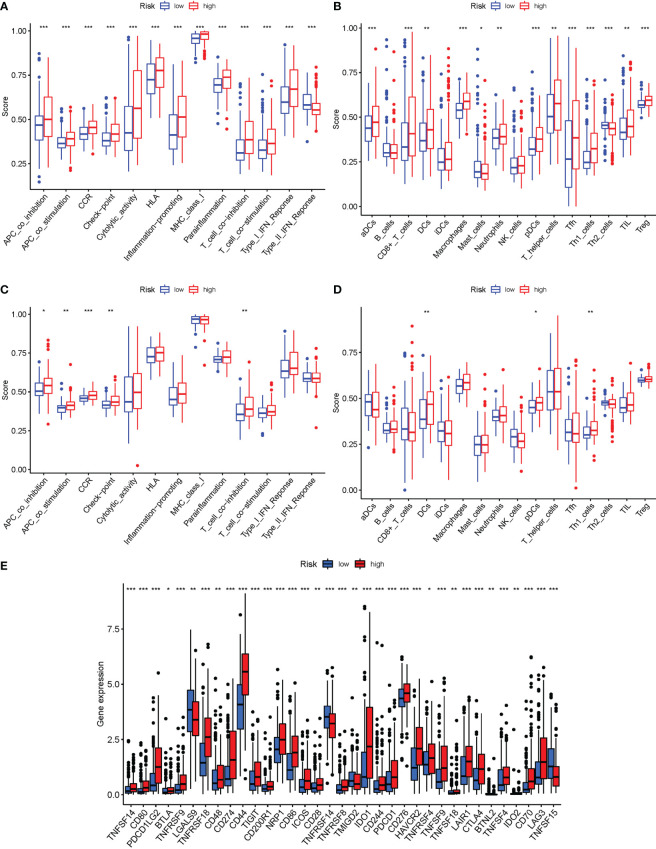
The ssGSEA scores are compared. **(A, B)** Comparison of the enrichment scores of 16 kinds of immune cells and 13 immune-related pathways in the TCGA cohort between the low-risk (green box) and high-risk (red box) groups. **(C, D)**: In the GEO cohort, tumor immunity was compared between the low-risk (blue box) and high-risk (red box) groups. P values were shown as follows: ns not significant; *P < 0.05; **P < 0.01; ***P < 0.001. **(E)** Immune checkpoint.

In the two cohorts, the enrichment scores of 16 types of immune cells and the activity of 13 immune-related activities in risk groups (ssGSEA) were evaluated. The low-risk group had a higher rate of Type II IF NReponse (**Figure 10A**). The low-risk category had more significant infiltration of Mast cells and Th2 cells (**Figure 10B**). Similar conclusions were obtained in the immunological condition of the GEO cohort (**Figures 10C, D**). Given the importance of checkpoint inhibitor-based immunotherapies, changes in immune checkpoint expression between the two groups were analyzed. LGALS9, TNFRSF14, TMIGD2 and TNFSF15 had a higher rate in low-risk group, as well as other genes, showed considerable alterations between the two groups (**Figure 10E**).

### 3.11 mRNA chemical modifications

**Figure 11:**
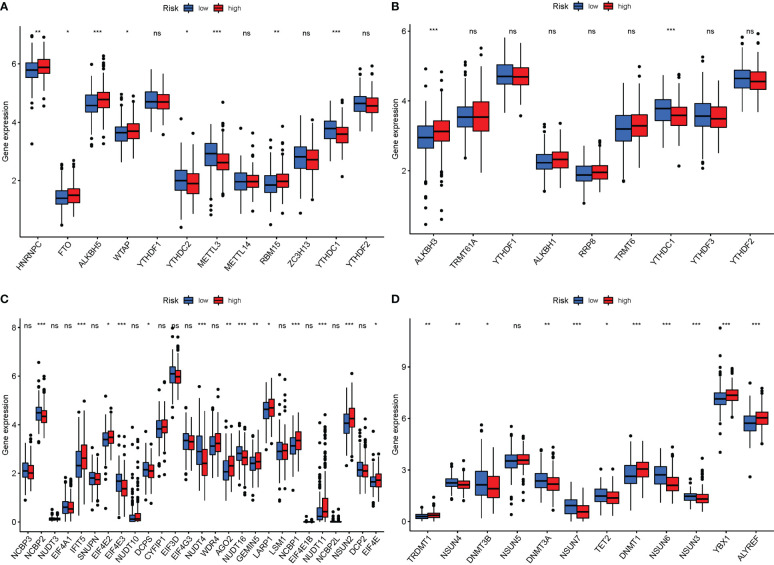
mRNA chemical modifications. **(A)** m6A (HNRNPC, FTO, ALKBH5, WTAP, and RBM15 were significantly more significant in the high-risk group. In the low-risk group, YTHDC2, METTL3, and RBM15 were significantly more significant). **(B)** M1A (ALKBH3 was substantially more significant in the high-risk group). **(C)** M7G (IFIT5, AGO2, GEMIN5, LARP1, NCBP1, NUDT11, NSUN2, and EIF4E were substantially more significant in the high-risk group). **(D)** M5C (TRDMT1, DNMT1, YBX1, and ALYREF were substantially more significant in the high-risk group). P values were showed as: ns, not significant; *P < 0.05; **P < 0.01; ***P < 0.001.

Currently, 172 distinct kinds of RNA alterations are known. The most common chemical modifications are M6A, m1A, M7G, and m5C. When PyMGs expression in M6a was compared between the two risk groups, HNRNPC, FTO, ALKBH5, WTAP, and RBM15 were more significant in the high-risk group. In the low-risk group, YTHDC2, METTL3, and RBM15 were more significant (**Figure 11A**). In M1A, ALKBH3 was more significant in the high-risk group (**Figure 11B**). In M7G, IFIT5, AGO2, GEMIN5, LARP1, NCBP1, NUDT11, NSUN2, and EIF4E were more significant in the high-risk group (**Figure 11C**). In M5C, TRDMT1, DNMT1, YBX1, and ALYREF were more significant in the high-risk group (**Figure 11D**).

## 4 Discussion

Bladder cancer (BLCA) remains the most prevalent type of urinary tract cancer (23), with its incidence increasing with age, particularly in individuals aged between 50 and 70 years. While the age-standardized incidence of BLCA exhibits considerable variation among different geographical locations, it is expected to rise further in the coming decade (24). Gender and exposure to various carcinogens, such as smoking, have significant impacts on BLCA risks (25). Age-standardized mortality rates for BLCA have declined in industrialized countries but exhibit an increasing trend in low-income areas. The primary symptom of BLCA is microscopic or extensive hematuria (26). The detection rate of urothelial bladder cancer (UBC) localized to the bladder mucosa is approximately 75% when a bladder tumor is discovered. Despite advances in surgical and anesthesiological techniques as well as widespread use of perioperative chemotherapy, long-term survival statistics for patients with UBC have remained stable for decades (27). To prevent the recurrence and progression of BLCA, prognostic indicators for high-risk populations must be identified through further research.

Cancer is believed to exhibite a fundamental trait of metabolic rewiring. Malignant cells alter their metabolic pathways in response to various intrinsic and extrinsic challenges to fuel cell survival and expansion (28). A vast body of evidence has shown that PyM failure is closely associated with cancer growth, and various medications targeting PyM have been authorized for a variety of cancers. As cancer evolves from premalignant lesions to clinically apparent tumors to metastatic malignancies, metabolic requirements, and phenotypes may emerge (10). Recent research on PyM aimed to examine the interaction between metabolic abnormalities and intrinsic genetic variation in cancer. It was discovered that KRAS mutant, PTEN deficient, and p53 deficient cells have enhanced pyrimidine *de novo* synthesis flow (8). This reliance on the pyrimidine pathway results in the synthetic lethal target of pyrimidine production in these cells, implying hardwired metabolic vulnerabilities in many gain-of-function mutant malignancies (29).The interconnection between the pyrimidine pathway and other metabolic pathways enables a better understanding of metabolic heterogeneity and the development of clinical therapeutic targeting techniques. Therefore, comprehending the complex cancer metabolism and identifying its weaknesses could accelerate the discovery of novel therapies for treating human cancer (9). The study of different PyM patterns in BLCA progression may aid in understanding PyM in BLCA progression, pointing to an appropriate therapy approach.

In this study, 76 DEGs associated with PyM were identified and categorized into two groups in BLCA. PyMGs were strongly related to BLCA prognosis, as prior research has shown that 7 prognostic PyMGs were expressed differentially in those at risk, with some PyMGs being overexpressed in the high-risk population (*P*<0.05). Furthermore, the role of PyMGs in BLCA was studied, and survival analysis was used to assess the prognostic value of PyMGs. Patients who got low-risk PyMGs showed a higher chance of survival. Moreover, DSG1, C6orf15, SOST, SPRR2A, SERPINB7, MYBPH, and KRT1 were considerably expressed in the high-risk group, indicating their potential roles as cancer-promoting genes in the development of BLCA. The findings of the aforementioned genes provide insights for future studies, but significant evidence for their involvement in the expression of certain transcription factors involved in the control of iron toxicity (e.g. Fin56, NRF2, and SFRS9) is inadequately studied (30–32).

Through an extensive literature review, it was discovered that these genes are associated with BLCA and PyM. MYBPH is a transcriptional target of TTF-1, a master regulator of lung development that acts as a lineage-survival oncogene in the formation of lung cancer (33). Aimy Sebastian discovered that a lower SOST expression in the tumor microenvironment may enhance bone metastasis in prostate cancer *via* up-regulation of MALAT1 in an *in vitro* co-culture model of PC3 prostate cancer cells and osteoblasts (34). In gastric cancer, serum small proline-rich protein 2A (SPRR2A) is a noninvasive biomarker (35). Protein expression of DSG1 was investigated by immunohistochemistry in a cohort of 53 anal cancer patients treated with radiation alone or in combination with 5-fluorouracil and mitomycin C in Myklebust’s research. They found that DSG1 expression is a prognostic predictor in individuals with anal cancer (36). According to Funosas, KRT1 is overexpressed in squamous carcinomas and is linked to aggressive pathology in breast cancer (37). These investigations corroborate and reinforce our findings since these 7 PyMGs were linked to the development of BLCA. The OS and ROC analyses of the Kaplan-Meier curves for GSE13507, GSE48075, and GSE48276 demonstrated that a PyM-related signature may serve as a useful prognostic predictor. Nevertheless, little knowledge is available related to the gene alterations associated with PyM. As a result, more research is needed to discover the mechanism of PyMGs changes, as well as to identify and confirm present findings.

Based on KEGG analysis, it was found that the genes were predominantly engaged in the Pyrimidine, Nucleotide, Purine metabolism, RNA polymerase. Many lipid metabolism-related genes, including ACLY, were found to be abnormally expressed in endometrial cancer tissues in Dai’s investigation. ACLY upregulation increased EC cell proliferation and colony formation while decreasing apoptosis (9). Zhu found that UBE2T was favorably associated with PyM, and that key PyM products were significantly increased in UBE2T-overexpressing cells. UBE2T overexpression boosted the activity of multiple key enzymes involved in *de novo* pyrimidine synthesis, including CAD, DHODH, and UMPS (38). 17 metabolites separated BCa patients’ urine profiles from healthy individuals’ urine profiles in a study by Jacyna. These metabolites are mostly involved in amino acid metabolism, purine and PyM metabolism, and energy metabolism, and they may play a role in BCa pathogenesis (39). The nod like receptor signaling pathway was considered the most highly enriched route in GSEA. Recent investigations have found that some botanicals and natural items can control NOD-like receptor signaling. NOD-like receptors (NLRs) have been identified as critical regulators of carcinogenesis, angiogenesis, cancer cell stemness, and chemoresistance in response to inflammation (40, 41). NLRs detect pathogen-associated molecular patterns and respond by activating other signaling regulators such as Rip2 kinase, NF-B, MAPK, and ASC/caspase-1, resulting in cytokine production (42). Considering the aforementioned features, PyMGs may alter BLCA cell migration and proliferation *via* influencing the nod-like receptor signaling pathway. A number of clinical trials have also demonstrated that PyM has an effect on BLCA patient survival.

Pro-inflammatory signaling is linked to Th1* cells in rheumatoid arthritis, multiple sclerosis, and Crohn’s disease. This specific subset of cells plays a crucial role in the defense against infection and the management of autoimmunity and inflammation (43). Previous research has focused on the transcriptome of immunological hallmark genes in human Th1* cells. More than 400 genes are expressed differently in Th1* individuals compared to Th1, Th2, and Th17 populations. A study of the functional networks of genes specifically up-regulated in Th1* revealed enrichment of components related to T cell proliferation, effector activities, and MTB immunity (44). Yulia Kushnareva discovered that through disrupting cellular pyrimidine metabolism, ISOC1 deletion lowered IFN and IL-17 production. The findings show that functional screening may be used to validate immunological signature genes and emphasize the role of metabolic fitness in adaptive immunity, demonstrating that pyrimidine metabolism is linked to immunotherapy and invasion. The survival of BLCA patients was successfully predicted in this research. An increase in the risk score, according to the PMG’s prognostic model, is connected to an increase in mortality and the high-risk ratio. PyMGs may serve as useful biomarkers for predicting the outcomes of BLCA patients. Recent research has found a relationship between various cell death mechanisms and anticancer immunity (45).In ICI-resistant cancers, the activation of proptosis, ferroptosis, and necroptosis resulted in synergistically improved anticancer efficiency (46). Insulin involvement in immune checkpoint regulation enhances PD-L1 expression in pancreatic ductal adenocarcinoma cells *via* many routes in the three cell lines studied, including increased InsR-A expression in A818-6 cells and modification of the adaptor protein Gab1 in BxPc3 cells (47). Kyrollis Attalla has identified TIM-3 and TIGIT as viable targets for monotherapy or in conjunction with other immune checkpoint inhibitors in patients with urothelial cancer of the bladder. A microscopic examination of the interaction between ICI, m6a, and PyM was carried out, and the results indicated a link between PyMGs alterations and the origin and development of BLCA.

The relationship between PyM and BLCA has been marginally explored. Studies have used bioinformatics analysis to analyze a relationship between PyM and cancer (10, 48). DEG analysis was used by Wu et al. to identify DEGs in the pyrimidine metabolic signaling pathway. They discovered NT5E, DPYS, and UPP1, three genes that are substantially expressed in GC. Wang et al. created a novel Lung cancer prediction model that integrates 5 PyMGs, including P2RX1, P2RX7, P2RY12, P2RY13, and P2RY14, which might be utilized to predict prognosis in Lung cancerpatients. There are now several studies on BLCA, including bioinformatics. Xia et al. provided uncertain ideas for the prevention and treatment of BLCA based on iron death or lncRNA through bioinformatics, and also gave certain references in the calculation model (49–51).

Despite the existing literature on the relationship between PyMGs and cancer. there remains a dearth of predictive models for PyMGs and cancer. This study adopted a novel approach to the investigation of this relationship. First, the current study expanded on earlier research by utilizing more PyMGs data from the continuously updated TCGA database. Second, TCGA data were used as the primary analysis, with GEO data being incorporated into the common pattern for model validation. The GO and KEGG analyses, as well as the GSEA analysis, all added credibility to the study. Finally, there is almost no prediction model for Pyrimidine metabolism genes that gives specific recommendations for future metabolic research or therapy based on metabolic interference BLCA. The present study has the following limitations. First, the current study expanded on prior research by using more PyMGs data from the TCGA database, which is regularly updated. Second, TCGA data were employed as the primary source of analysis, with GEO data being used to validate the model using a similar pattern. The GO and KEGG analyses, as well as the GSEA research, all supported the findings. Fourth, several databases were employed to examine immune cells and function in order to increase the trustworthiness of the results. The following are the study’s challenges. This risk model is mostly based on publicly accessible databases. Furthermore, protein expression may differ from RNA expression, necessitating additional research with more data collection.

## 5 Conclusions

For BLCA, the present study identified seven expected regulatory patterns of PyMGs, along with transcriptome and immune infiltration features. The current study elucidates the functions of PyMGs regulators and provides insight into the reasons for varied clinical outcomes and immunotherapy responses across different PyMGs regulatory patterns. The diverse and complex TME is influenced by multiple PyMGs changing patterns. Our data suggest that PyMGs may serve as promising prognostic markers, which could lead to novel BLCA treatment alternatives for BLCA.

## Data availability statement

The original contributions presented in the study are included in the article/**Supplementary Material**. Further inquiries can be directed to the corresponding authors.

## Author contributions

ZW drafted and revised the manuscript. XL and ZG were in charge of data collection. JY and XX conceived and designed this article, in charge of syntax modification and revised of the manuscript. All authors contributed to the article and approved the submitted version. After revised contributions and all authors agree, agree to join these authors, and so the responsible author is solely responsible.
