# Supplementary material for: Pyrimidine metabolism regulator-mediated molecular subtypes display tumor microenvironmental hallmarks and assist precision treatment in bladder cancer
Source: Front Oncol. 2023 Aug 17;13:1102518. doi: 10.3389/fonc.2023.1102518 (PMC10470057; doi:10.3389/fonc.2023.1102518)
Supplement: Supplementary file 1 [file DataSheet_1.doc]

Pyrimidine Metabolism Regulator-Mediated Molecular Subtypes Display Tumor Microenvironmental Hallmarks and Assist Precision Treatment in Bladder Cancer

**Supplementary appendix to the manuscript**

Contents of supplementary appendix

[Appendix 1 3](#__RefHeading___Toc18887)

[Datasets and Pyrimidine Metabolism 3](#__RefHeading___Toc19186)

[Table 1. Pyrimidine Metabolism genes 3](#__RefHeading___Toc31181)

[Appendix 2 4](#__RefHeading___Toc11930)

[Table 2a. m6A genes. 4](#__RefHeading___Toc713)

[Table 2b. m1A genes. 4](#__RefHeading___Toc19048)

[Table 2c. m7G genes. 4](#__RefHeading___Toc4917)

[Table 2d. m5C genes. 5](#__RefHeading___Toc21828)

[Appendix 3 5](#__RefHeading___Toc1888)

[DEGs linked to FAMGs 5](#__RefHeading___Toc24354)

[Table 3. 76 DEGs linked to purine metabolism genes. 5](#__RefHeading___Toc18636)

[Appendix 4 8](#__RefHeading___Toc18285)

[The drug prediction of the model 8](#__RefHeading___Toc11618)

[Appendix 5 9](#__RefHeading___Toc22506)

[Correlation analysis of gene expression in prognostic signatures and drug sensitivity 10](#__RefHeading___Toc32071)

[Appendix 6 10](#__RefHeading___Toc12223)

[hub genes analysis 10](#__RefHeading___Toc15450)

[Table 6. Hub genes. 11](#__RefHeading___Toc18289)

[Appendix 7 13](#__RefHeading___Toc31907)

[The gene expression profile and clinical characteristics 14](#__RefHeading___Toc24092)

[Table 7. The gene expression profile and clinical characteristics. 14](#__RefHeading___Toc26937)

[Appendix 8 15](#__RefHeading___Toc24887)

[7 risk PRGs 15](#__RefHeading___Toc30064)

[Table 8. 7 risk PRGs. 16](#__RefHeading___Toc11162)

[Appendix 9 44](#__RefHeading___Toc14920)

[GO and KEGG enrichment analysis 44](#__RefHeading___Toc7594)

[Table 9a. GO enrichment analysis. 45](#__RefHeading___Toc7280)

[Table 9b. KEGG enrichment analysis. 67](#__RefHeading___Toc24524)

[Appendix 10 69](#__RefHeading___Toc26465)

[gene set enrichment analyses (GSEA) 69](#__RefHeading___Toc27026)

[Table 10a. GSEA of high rish. 70](#__RefHeading___Toc11872)

[Table 10b. GSEA of low rish. 81](#__RefHeading___Toc31556)

# Appendix 1

**Datasets and Pyrimidine Metabolism**

**Table 1. Pyrimidine Metabolism genes**

| CAD | POLR1D | DCTD | ENPP1 | TWISTNB |
| --- | --- | --- | --- | --- |
| DHODH | POLR3H | TK2 | ENPP3 | ENTPD6 |
| UMPS | POLR3GL | TK1 | TXNRD1 | CTPS1 |
| CMPK1 | POLR3G | DTYMK | TXNRD2 | CTPS2 |
| CMPK2 | POLR3F | NUDT2 | TXNRD3 | POLR1A |
| NME6 | POLA1 | POLR2H | RRM1 | POLR1B |
| NME7 | POLA2 | POLR2I | RRM2B | ZNRD1 |
| NME2 | PRIM1 | POLR2L | RRM2 | POLR2C |
| NME4 | PRIM2 | POLR2J | DCTPP1 | POLR2D |
| NME1 | POLD1 | POLR2J3 | DUT | POLR2E |
| NME3 | POLD2 | POLR2J2 | TYMS | POLR2F |
| NME1-NME2 | POLD3 | POLR2K | CDA | POLR2G |
| AK9 | POLD4 | POLR3A | TYMP | POLR1E |
| PNPT1 | POLE | POLR3B | PNP | UPP1 |
| ENTPD3 | POLE2 | POLR3C | DCK | UPRT |
| ENTPD8 | POLE3 | POLR3D | NT5C2 | DPYD |
| ENTPD1 | POLE4 | POLR3E | NT5C1A | DPYS |
| CANT1 | UCK1 | RPC5 | NT5C1B | UPB1 |
| ENTPD4 | UCK2 | POLR1C | NT5C | POLR2A |
| ENTPD5 | UCKL1 | POLR3K | NT5M | NT5C3A |
| POLR2B | UPP2 | NT5E | NT5C1B-RDH14 | NT5C3B |

# Appendix 2

**mRNA chemical modifications**

**Table 2a. m6A genes.**

| METTL3 | YTHDC1 | ZC3H13 | RBM15 | HNRNPC |
| --- | --- | --- | --- | --- |
| METTL14 | YTHDC2 | FTO | YTHDF2 | KIAA1429 |
| WTAP | YTHDF1 | ALKBH5 |  |  |

**Table 2b. m1A genes.**

| YTHDF2 | YTHDF1 | TRMT61A | YTHDC1 | YTHDF3 |
| --- | --- | --- | --- | --- |
| RRP8 | ALKBH1 | ALKBH3 | TRMT6 |  |

**Table 2c. m7G genes.**

| METTL1 | EIF4E | EIF4A1 | NUDT4 | NCBP1 |
| --- | --- | --- | --- | --- |
| WDR4 | EIF4E1B | EIF4G3 | NUDT48 | NCBP2 |
| NSUN2 | EIF4E2 | IFIT5 | AGO2 | NCBP3 |
| DCP2 | EIF4E3 | LSM1 | CYFIP1 | EIF3D |
| DCPS | GEMIN5 | NCBP2L | NUDT16 | NUDT11 |
| NUDT10 | LARP1 | SNUPN | NUDT3 |  |

**Table 2d. m5C genes.**

| NSUN1 | DNMT2 | NSUN7 | TET2 | NSUN4 |
| --- | --- | --- | --- | --- |
| NSUN | DNMT3A | ALYREF | TRDMT1 | NSUN5 |
| NSUN3 | DNMT3B | DNMT1 | YBX1 | NSUN6 |

# Appendix 3

## **DEGs linked to FAMGs**

**Table 3. 76 DEGs linked to purine metabolism genes.**

| gene | conMean | treatMean | logFC | pValue |
| --- | --- | --- | --- | --- |
| CAD | 4.955726316 | 10.44211019 | 1.075244868 | 1.73E-08 |
| DHODH | 1.458805263 | 2.184296117 | 0.58238114 | 1.09E-05 |
| UMPS | 3.778926316 | 6.681419417 | 0.822178237 | 2.64E-07 |
| CMPK2 | 1.496505263 | 5.052232524 | 1.755323687 | 4.90E-05 |
| NME6 | 2.188647368 | 3.355904854 | 0.616662285 | 2.04E-06 |
| NME2 | 0.231384211 | 0.631837379 | 1.449262868 | 1.44E-07 |
| NME1 | 3.801884211 | 10.66328592 | 1.487865578 | 2.73E-10 |
| NME3 | 23.9072 | 31.11527937 | 0.380178027 | 0.036447038 |
| PNPT1 | 5.031947368 | 8.485930583 | 0.753956045 | 4.10E-07 |
| ENTPD8 | 0.061089474 | 1.204743447 | 4.301658332 | 0.012053775 |
| ENTPD1 | 4.975915789 | 2.22391165 | -1.161862595 | 4.10E-05 |
| CANT1 | 11.55090526 | 20.868725 | 0.853336538 | 1.24E-06 |
| ENTPD6 | 5.930252632 | 12.73940704 | 1.103132658 | 2.51E-09 |
| CTPS2 | 4.106736842 | 5.589102427 | 0.444624111 | 0.003821908 |
| POLR1A | 3.6594 | 5.143890534 | 0.49125282 | 0.001092782 |
| POLR1B | 2.255005263 | 3.919981796 | 0.797716154 | 1.60E-06 |
| POLR1E | 13.11182632 | 10.03986481 | -0.385128807 | 0.007752084 |
| POLR2D | 4.689589474 | 6.90170267 | 0.557490688 | 4.71E-06 |
| POLR2E | 27.47598421 | 33.91892184 | 0.303919152 | 0.003620625 |
| POLR2F | 4.463021053 | 6.94216335 | 0.6373647 | 1.55E-06 |
| POLR2G | 21.16087895 | 41.6199301 | 0.975874989 | 1.81E-09 |
| POLR2H | 10.79895263 | 25.00149587 | 1.211123021 | 2.45E-10 |
| POLR2I | 2.978510526 | 5.611044417 | 0.913678276 | 2.99E-07 |
| POLR2L | 69.31041053 | 93.2353466 | 0.427804937 | 0.000447486 |
| POLR2J | 22.2345 | 41.34390825 | 0.894874811 | 2.23E-07 |
| POLR2J3 | 0.01 | 0.044654612 | 2.158809176 | 1.60E-06 |
| POLR2J2 | 0.035315789 | 0.089865534 | 1.347454559 | 0.003136299 |
| POLR2K | 23.24354737 | 41.42327524 | 0.833611363 | 2.43E-06 |
| POLR3A | 2.894268421 | 3.753154369 | 0.374904903 | 0.000438068 |
| POLR3B | 2.401678947 | 3.255493204 | 0.43883282 | 0.003316532 |
| POLR3C | 5.645489474 | 7.217371117 | 0.354374769 | 0.004514837 |
| POLR3E | 3.281084211 | 4.174754612 | 0.347518778 | 0.016273481 |
| POLR1C | 3.697273684 | 6.217155583 | 0.749792842 | 2.65E-05 |
| POLR3K | 7.044 | 11.19791141 | 0.668762857 | 1.67E-06 |
| POLR1D | 8.503363158 | 11.56664005 | 0.443864384 | 0.000390889 |
| POLR3H | 2.631057895 | 3.237213592 | 0.299109564 | 0.01272909 |
| POLR3GL | 7.686521053 | 6.47935801 | -0.246479902 | 0.010381916 |
| POLR3G | 0.505173684 | 1.131371359 | 1.163221162 | 0.011107577 |
| POLR3F | 4.261678947 | 5.609759709 | 0.396517063 | 0.003017345 |
| POLA1 | 1.205610526 | 1.771375 | 0.555105744 | 0.013777848 |
| POLA2 | 0.733868421 | 1.874884466 | 1.353208373 | 2.16E-09 |
| PRIM1 | 0.782768421 | 2.119496359 | 1.437064028 | 1.58E-07 |
| PRIM2 | 1.774515789 | 3.548624029 | 0.99983432 | 8.06E-09 |
| POLD1 | 2.675657895 | 7.96053568 | 1.572971849 | 7.94E-11 |
| POLD2 | 19.87543158 | 35.01331141 | 0.816917324 | 4.13E-06 |
| POLD3 | 3.995942105 | 5.4867 | 0.457403014 | 0.004009303 |
| POLD4 | 2.088231579 | 3.151276699 | 0.593654725 | 0.000290762 |
| POLE | 1.870484211 | 4.227539806 | 1.176406551 | 2.84E-09 |
| POLE2 | 0.787910526 | 2.820518932 | 1.839856906 | 1.15E-09 |
| POLE3 | 18.53132632 | 28.84271602 | 0.638240884 | 5.94E-05 |
| POLE4 | 16.29894211 | 22.58602354 | 0.470651966 | 0.000497475 |
| UCK1 | 9.191026316 | 13.05901286 | 0.506747973 | 0.001469134 |
| UCK2 | 2.462615789 | 5.518933981 | 1.164198068 | 9.32E-08 |
| UCKL1 | 8.826626316 | 16.20337864 | 0.876360641 | 3.73E-08 |
| NT5C1A | 0.031315789 | 0.011026942 | -1.505857526 | 0.019358539 |
| NT5C | 9.244805263 | 16.26957961 | 0.815462138 | 1.31E-05 |
| NT5M | 1.736805263 | 1.349306796 | -0.364217587 | 0.013490964 |
| NT5C3A | 6.517136842 | 10.50475558 | 0.688732402 | 2.11E-05 |
| NT5C3B | 11.58985263 | 15.26842743 | 0.397689257 | 0.013139808 |
| NT5E | 8.641731579 | 8.926609709 | 0.046791929 | 0.003054518 |
| UPP1 | 10.62533684 | 15.10020558 | 0.507059613 | 0.002355473 |
| DPYD | 5.039878947 | 3.397413835 | -0.56895212 | 0.000260561 |
| ENPP1 | 1.074336842 | 0.875247087 | -0.295684139 | 1.08E-05 |
| ENPP3 | 0.222310526 | 0.066632524 | -1.738277807 | 3.51E-08 |
| TXNRD1 | 33.34712105 | 18.48595291 | -0.851132802 | 0.007752084 |
| TXNRD3 | 2.045668421 | 3.115809223 | 0.607034582 | 0.000151155 |
| RRM1 | 14.12525789 | 20.18890874 | 0.515285723 | 0.033518889 |
| RRM2 | 3.198436842 | 11.86983228 | 1.89186065 | 7.97E-08 |
| DCTPP1 | 16.66056842 | 26.85737015 | 0.688880421 | 3.87E-06 |
| DUT | 11.26334737 | 16.86453325 | 0.582356743 | 2.17E-05 |
| TYMS | 1.220294737 | 4.215602427 | 1.78850917 | 4.33E-08 |
| TYMP | 12.25549474 | 41.07923689 | 1.744980657 | 0.000185267 |
| DCK | 4.867947368 | 7.874188592 | 0.693817697 | 0.000205685 |
| DCTD | 18.14561579 | 23.207925 | 0.354996521 | 0.001704614 |
| TK1 | 13.24651579 | 59.05623592 | 2.156476466 | 1.73E-09 |
| DTYMK | 7.655889474 | 18.2439267 | 1.252774372 | 1.40E-09 |

# Appendix 4

**The drug prediction of the model**


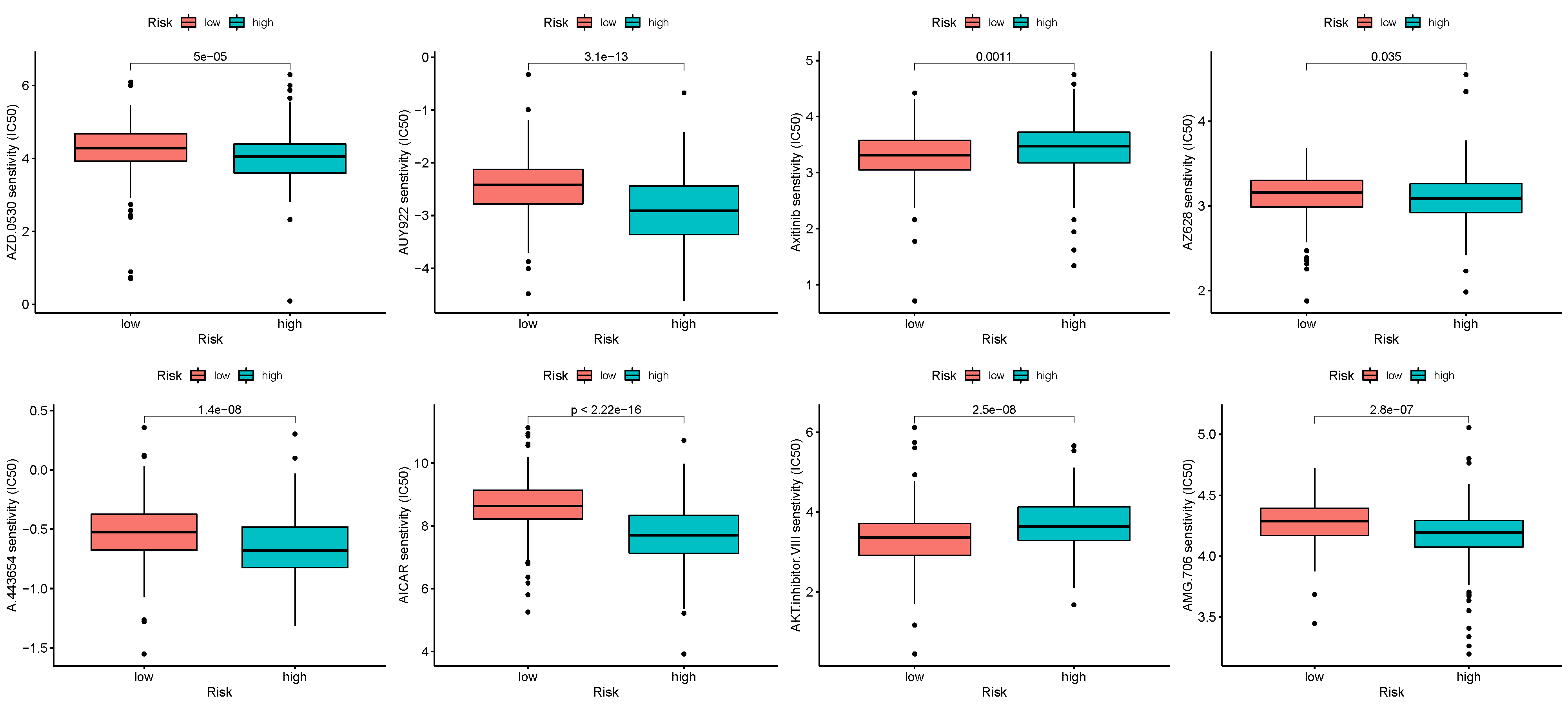


Figure S1. The drug prediction of the model.

# Appendix 5

## **Correlation analysis of gene expression in prognostic signatures and drug sensitivity**


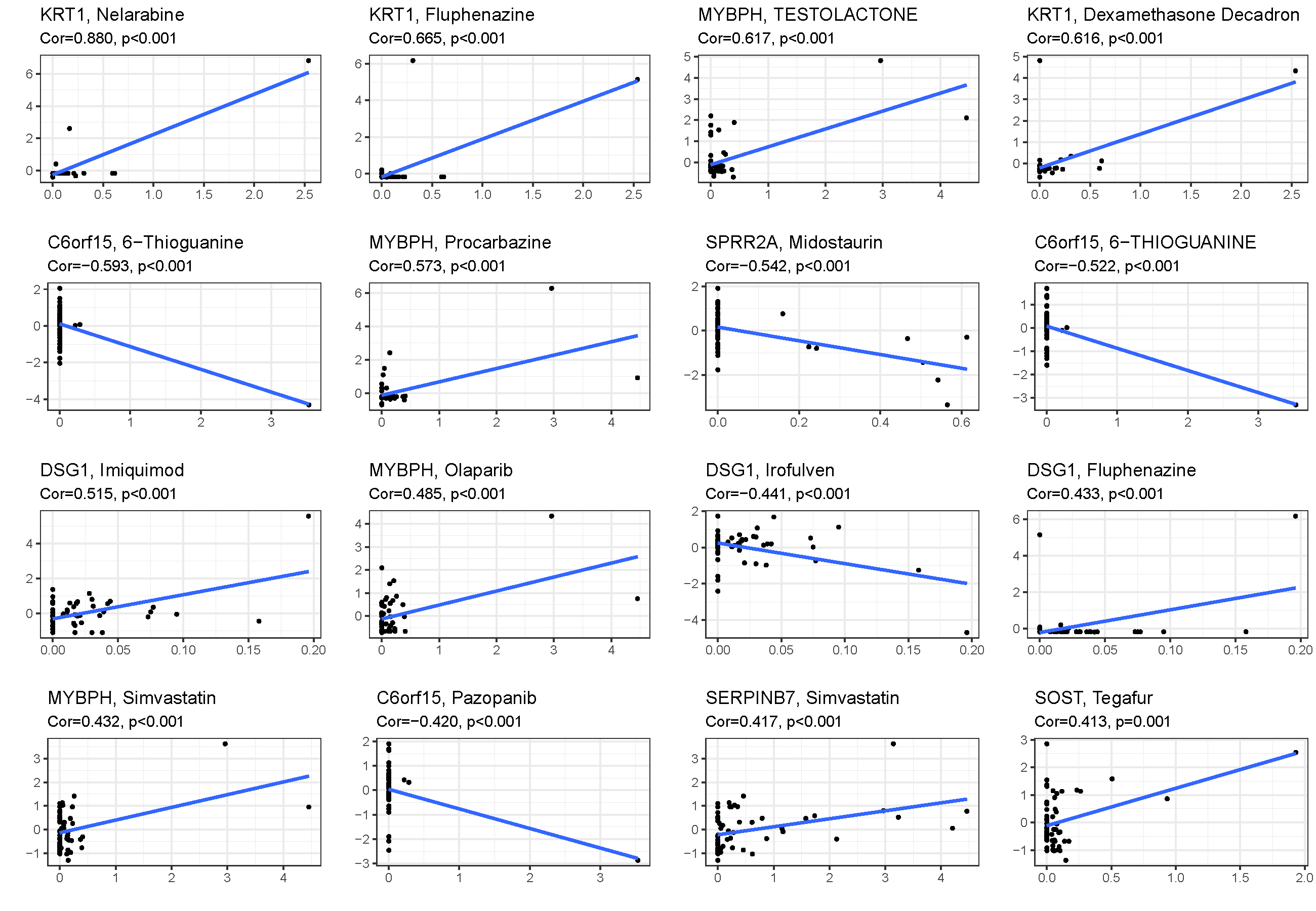


Figure S2. Correlation analysis of gene expression in prognostic signatures and drug sensitivity (KRT1, MYBPH, C6orf15, SPRR2A, DSG1, SERPINB7, and SOST).

# Appendix 6

## **hub genes analysis**

**Table 6. Hub genes.**

| name | Betweenness | Closeness | Degree | Network |
| --- | --- | --- | --- | --- |
| CAD | 0 | 0.36453202 | 2 | 2 |
| DHODH | 648.1006735 | 0.415730337 | 3 | 1.5 |
| UMPS | 825.4536241 | 0.474358974 | 25 | 20.56541219 |
| CANT1 | 109.5637013 | 0.411111111 | 15 | 7.942063492 |
| NT5C1A | 19.35941547 | 0.397849462 | 18 | 14.09345238 |
| NT5C | 19.35941547 | 0.397849462 | 18 | 14.09345238 |
| NT5E | 45.75567111 | 0.404371585 | 21 | 16.04958938 |
| UCKL1 | 3.699911853 | 0.36097561 | 10 | 8.111111111 |
| NME1 | 157.5963788 | 0.422857143 | 11 | 8.1 |
| ENTPD8 | 418.9292856 | 0.47133758 | 24 | 18.20342481 |
| NME6 | 180.4516902 | 0.430232558 | 14 | 9.513461538 |
| NME2 | 157.5963788 | 0.422857143 | 11 | 8.1 |
| NT5M | 22.00583689 | 0.4 | 19 | 14.82542017 |
| UCK1 | 3.699911853 | 0.36097561 | 10 | 8.111111111 |
| ENTPD6 | 3.908509911 | 0.359223301 | 9 | 6.5 |
| CTPS2 | 1759.81924 | 0.443113772 | 11 | 4.233333333 |
| ENPP3 | 79.95743194 | 0.408839779 | 14 | 10.57200855 |
| ENPP1 | 65.50268512 | 0.406593407 | 13 | 10.05277778 |
| CMPK2 | 138.1358017 | 0.420454545 | 25 | 19.36193342 |
| NME3 | 150.6503528 | 0.420454545 | 10 | 8.222222222 |
| DUT | 241.6095237 | 0.408839779 | 22 | 13.85610727 |
| DTYMK | 47.92231385 | 0.408839779 | 20 | 14.29163874 |
| ENTPD1 | 334.3067191 | 0.459627329 | 20 | 13.34191526 |
| NT5C3B | 15.18246765 | 0.395721925 | 17 | 13.15260989 |
| RRM2 | 210.8423035 | 0.4 | 18 | 11.00833513 |
| UCK2 | 2.438757321 | 0.359223301 | 9 | 7.75 |
| DCTD | 8.972954924 | 0.385416667 | 15 | 12.22619048 |
| DCTPP1 | 24.32141032 | 0.381443299 | 15 | 9.042490842 |
| NT5C3A | 21.12640387 | 0.395721925 | 17 | 12.94583333 |
| DCK | 34.44614218 | 0.389473684 | 18 | 12.35719672 |
| RRM1 | 748.5399216 | 0.411111111 | 22 | 13.31262398 |
| POLR1A | 645.7891955 | 0.381443299 | 17 | 14.32115385 |
| POLR1B | 970.8520142 | 0.389473684 | 21 | 18.50035751 |
| TYMS | 561.873722 | 0.430232558 | 24 | 16.37248282 |
| TK1 | 36.98375259 | 0.397849462 | 15 | 10.24285714 |
| UPP1 | 20.03956044 | 0.354066986 | 13 | 7.758333333 |
| POLR1C | 650.5577709 | 0.375634518 | 25 | 23.8875 |
| DPYD | 1.568577415 | 0.355769231 | 4 | 3.333333333 |
| TYMP | 6.372423569 | 0.37 | 11 | 9.3 |
| POLA2 | 105.3282062 | 0.339449541 | 14 | 12.30128205 |
| POLE | 105.3282062 | 0.339449541 | 14 | 12.30128205 |
| PRIM1 | 99.09298455 | 0.339449541 | 14 | 11.50909091 |
| POLA1 | 134.904171 | 0.341013825 | 15 | 13.07142857 |
| POLE2 | 104.7336654 | 0.339449541 | 14 | 12.30769231 |
| POLD3 | 0.444444444 | 0.264285714 | 11 | 10.8 |
| POLD4 | 0 | 0.262411348 | 9 | 9 |
| POLE3 | 0.444444444 | 0.264285714 | 11 | 10.8 |
| POLE4 | 0.444444444 | 0.264285714 | 11 | 10.8 |
| POLD1 | 30.67755212 | 0.312236287 | 12 | 11.36363636 |
| POLD2 | 0.444444444 | 0.264285714 | 11 | 10.8 |
| PRIM2 | 76.55425533 | 0.337899543 | 13 | 11.00909091 |
| POLR3B | 10.79661125 | 0.317596567 | 20 | 18.76004042 |
| POLR2G | 11.8341679 | 0.316239316 | 19 | 17.26143791 |
| POLR3H | 9.727563635 | 0.316239316 | 19 | 17.60947712 |
| POLR2D | 10.49537891 | 0.314893617 | 18 | 16.56092437 |
| POLR3F | 11.35086811 | 0.317596567 | 20 | 18.60008194 |
| POLR3C | 9.534900742 | 0.316239316 | 19 | 17.90918803 |
| POLR2K | 19.68273589 | 0.32173913 | 23 | 22.38361653 |
| POLR3A | 24.32196852 | 0.32173913 | 23 | 22.31636254 |
| POLR2F | 26.31629087 | 0.323144105 | 24 | 23.8875 |
| POLR2L | 26.31629087 | 0.323144105 | 24 | 23.8875 |
| POLR1D | 11.60449781 | 0.318965517 | 21 | 20.012629 |
| POLR2E | 19.68273589 | 0.32173913 | 23 | 22.38361653 |
| POLR2H | 19.68273589 | 0.32173913 | 23 | 22.38361653 |
| POLR1E | 0.544047619 | 0.309623431 | 14 | 13.38461538 |
| POLR2I | 7.554955801 | 0.314893617 | 18 | 16.46250808 |
| POLR2J | 4.862342858 | 0.31092437 | 15 | 14.28571429 |
| POLR3K | 6.633136131 | 0.317596567 | 20 | 18.41748836 |
| POLR3E | 4.727244744 | 0.313559322 | 17 | 16.25 |
| POLR2J2 | 0 | 0.285714286 | 12 | 12 |
| POLR3GL | 0.142857143 | 0.285714286 | 12 | 11.81818182 |
| POLR2J3 | 0 | 0.285714286 | 12 | 12 |
| POLR3G | 0.5 | 0.287937743 | 14 | 13.67832168 |
| TXNRD1 | 146 | 0.294820717 | 2 | 0 |
| TXNRD3 | 0 | 0.228395062 | 1 | 0 |

# Appendix 7

# **The gene expression profile and clinical characteristics**

**Table 7. The gene expression profile and clinical characteristics.**

| gene | Mean1 | Mean2 | logFC | pValue | fdr |
| --- | --- | --- | --- | --- | --- |
| INSM1 | 3.324051351 | 0.171404891 | -4.277462485 | 0.024610888 | 0.035394932 |
| COL2A1 | 1.513090991 | 0.099028804 | -3.933506717 | 2.07E-05 | 5.32E-05 |
| SPINK6 | 4.712237387 | 0.270969022 | -4.120212388 | 0.000194112 | 0.000418973 |
| SPRR2E | 36.53576351 | 1.000240761 | -5.190890149 | 2.07E-06 | 6.28E-06 |
| TENM2 | 2.99566036 | 0.243630435 | -3.62010779 | 1.66E-05 | 4.34E-05 |
| DPYSL5 | 0.939557207 | 0.023079348 | -5.347306644 | 6.62E-06 | 1.85E-05 |
| LINC00973 | 0.996295045 | 0.056972826 | -4.128227172 | 4.28E-12 | 3.24E-11 |
| TAGLN3 | 1.567721171 | 0.126382609 | -3.632799135 | 6.83E-08 | 2.65E-07 |
| CYP2B6 | 1.121222973 | 0.016472826 | -6.088841313 | 0.004783354 | 0.007963778 |
| DSG1 | 4.216231982 | 0.276963043 | -3.928188859 | 3.36E-05 | 8.34E-05 |
| FGF19 | 3.615086937 | 0.190369565 | -4.247155587 | 0.020527936 | 0.030096025 |
| C6orf15 | 6.297987387 | 0.221871196 | -4.827096581 | 5.31E-08 | 2.10E-07 |
| SLC10A1 | 0.099387387 | 1.911061957 | 4.265167961 | 2.27E-05 | 5.79E-05 |
| KLK5 | 24.83468243 | 0.852378261 | -4.86471869 | 2.82E-13 | 2.62E-12 |
| SPRR2F | 4.274226126 | 0.149990217 | -4.832722921 | 0.000344265 | 0.00070915 |
| KRT81 | 41.67785991 | 2.021352717 | -4.365888207 | 6.22E-11 | 3.89E-10 |
| SOST | 4.175203153 | 0.216483152 | -4.26951974 | 0.003822854 | 0.006474399 |
| SPRR2A | 66.19441802 | 1.627046196 | -5.346382446 | 1.25E-09 | 6.46E-09 |
| SPRR2D | 32.43214955 | 2.073759783 | -3.967103954 | 8.48E-09 | 3.82E-08 |
| S100A7A | 5.774 | 0.408529348 | -3.82105948 | 2.49E-08 | 1.04E-07 |
| TUBB2B | 12.41819685 | 0.604527717 | -4.360503408 | 8.28E-13 | 7.06E-12 |
| ASCL1 | 2.547373423 | 0.062420652 | -5.350843221 | 0.003321402 | 0.005693128 |
| SERPINB7 | 4.350574324 | 0.355122826 | -3.614815865 | 1.29E-11 | 8.96E-11 |
| CGB8 | 2.087787387 | 0.164640217 | -3.664586103 | 0.000391075 | 0.00079565 |
| MYBPH | 3.434244144 | 0.026154348 | -7.036797999 | 0.000173701 | 0.000379166 |
| IL36G | 4.780741892 | 0.277263587 | -4.107904453 | 4.97E-07 | 1.68E-06 |
| ZFP42 | 1.052257207 | 0.040845109 | -4.687180259 | 0.00017977 | 0.000391163 |
| KRT1 | 56.71193514 | 2.628853261 | -4.431146865 | 0.000192908 | 0.000416674 |
| MAGEB2 | 2.946853604 | 0.134102174 | -4.457770861 | 0.000266971 | 0.000562343 |
| KRT34 | 1.799049099 | 0.147144565 | -3.611928398 | 9.80E-09 | 4.36E-08 |
| SPRR2G | 16.01842523 | 0.478430978 | -5.065277706 | 4.44E-07 | 1.51E-06 |
| AC083967.1 | 1.334611261 | 0.06507663 | -4.35813622 | 1.16E-06 | 3.67E-06 |

**Appendix 8**

**7 risk PRGs**

**Table 8. 7 risk PRGs.**

| id | DSG1 | C6orf15 | SOST | SPRR2A | SERPINB7 | MYBPH | KRT1 | risk |
| --- | --- | --- | --- | --- | --- | --- | --- | --- |
| TCGA-ZF-A9RN | 2.931899 | 2.76934 | 2.54112 | 2.429008 | 2.459915 | 3.257082 | 8.78884 | high |
| TCGA-CF-A1HR | 2.597324 | 2.76934 | 2.546338 | 2.382946 | 2.462976 | 3.237093 | 2.685695 | low |
| TCGA-XF-A8HH | 2.869911 | 2.76934 | 2.55736 | 2.645143 | 2.486826 | 3.232687 | 2.693166 | low |
| TCGA-ZF-AA4W | 3.552741 | 3.154458 | 2.574683 | 7.02559 | 5.358332 | 3.398852 | 3.990756 | high |
| TCGA-XF-A9SU | 2.661656 | 2.810621 | 2.497418 | 3.143062 | 2.64182 | 3.210315 | 3.067613 | low |
| TCGA-DK-A3IQ | 2.680817 | 2.866545 | 2.572003 | 2.417158 | 4.29387 | 3.197081 | 2.826405 | high |
| TCGA-FD-A6TK | 2.651704 | 3.947265 | 2.657392 | 3.039233 | 5.678848 | 3.238623 | 2.701309 | high |
| TCGA-DK-A1A7 | 2.613436 | 2.76934 | 2.507371 | 2.558983 | 2.434226 | 3.176865 | 3.414276 | low |
| TCGA-CU-A5W6 | 2.687102 | 2.76934 | 2.510851 | 4.921477 | 4.339483 | 3.250252 | 4.359507 | high |
| TCGA-DK-A3X1 | 2.815496 | 2.856873 | 2.561486 | 2.996316 | 2.434226 | 3.224808 | 3.119671 | low |
| TCGA-XF-A9ST | 2.602574 | 2.76934 | 2.497418 | 2.735936 | 2.434226 | 3.176865 | 2.615272 | low |
| TCGA-CF-A9FM | 2.597324 | 2.76934 | 2.497418 | 2.382946 | 2.785329 | 3.176865 | 2.628104 | low |
| TCGA-BT-A20V | 3.018928 | 3.083419 | 2.534963 | 2.382946 | 6.300741 | 3.558452 | 2.785941 | high |
| TCGA-GV-A3QF | 2.622821 | 2.76934 | 2.497418 | 2.423769 | 2.445584 | 3.176865 | 2.615272 | low |
| TCGA-DK-A2I4 | 2.597324 | 3.334901 | 2.584844 | 2.438939 | 2.659647 | 3.352297 | 2.699158 | low |
| TCGA-DK-A6B2 | 2.659617 | 2.76934 | 2.497418 | 2.627711 | 2.585552 | 3.22134 | 2.615272 | low |
| TCGA-DK-A1AG | 2.601015 | 2.794943 | 2.497418 | 3.610652 | 2.444102 | 3.176865 | 2.634912 | low |
| TCGA-XF-A9SZ | 2.633531 | 2.909482 | 3.501327 | 3.823096 | 2.542112 | 3.263701 | 2.670011 | high |
| TCGA-E7-A519 | 2.597324 | 2.858048 | 2.497418 | 2.613401 | 2.502411 | 3.483223 | 2.632521 | low |
| TCGA-2F-A9KO | 2.644573 | 2.806374 | 2.513655 | 2.529465 | 2.490348 | 3.20682 | 4.858913 | high |
| TCGA-ZF-A9R1 | 2.606533 | 2.801355 | 2.511519 | 2.978591 | 2.692708 | 3.176865 | 2.77731 | low |
| TCGA-4Z-AA7S | 2.623519 | 2.76934 | 2.497418 | 2.504976 | 2.434226 | 3.176865 | 2.638554 | low |
| TCGA-BL-A13J | 5.888462 | 2.952577 | 2.53489 | 4.571845 | 2.861187 | 3.191562 | 7.534836 | high |
| TCGA-DK-A3IK | 2.63989 | 2.85981 | 2.669212 | 3.172061 | 2.443158 | 3.176865 | 3.664037 | low |
| TCGA-C4-A0F1 | 3.378052 | 4.158249 | 2.597439 | 7.362665 | 3.099686 | 3.219024 | 5.62869 | high |
| TCGA-DK-A3IS | 2.634085 | 2.76934 | 2.497418 | 2.441831 | 2.442484 | 3.194345 | 2.99327 | low |
| TCGA-UY-A9PH | 2.634223 | 2.76934 | 2.497418 | 2.809528 | 2.454174 | 3.176865 | 4.61873 | high |
| TCGA-E5-A4TZ | 5.612351 | 3.596008 | 2.497418 | 7.041938 | 3.998842 | 3.212447 | 8.360981 | high |
| TCGA-DK-A3WW | 3.718727 | 2.953477 | 2.533517 | 3.778437 | 5.65237 | 3.243397 | 4.413036 | high |
| TCGA-K4-A5RH | 2.599597 | 2.785276 | 3.028704 | 2.447834 | 2.46457 | 3.189647 | 2.615272 | high |
| TCGA-ZF-AA56 | 3.166993 | 2.798458 | 3.139273 | 7.068151 | 3.277775 | 3.400614 | 4.580508 | high |
| TCGA-UY-A8OB | 2.723332 | 2.847584 | 2.514855 | 6.013167 | 4.16561 | 3.240534 | 2.864786 | high |
| TCGA-YC-A9TC | 2.751155 | 2.797542 | 2.746373 | 2.459282 | 2.538848 | 3.434489 | 2.787504 | high |
| TCGA-CF-A7I0 | 2.681487 | 2.76934 | 2.497418 | 2.515806 | 2.497365 | 3.176865 | 2.640809 | low |
| TCGA-K4-A5RJ | 2.602999 | 2.808499 | 2.531544 | 3.633735 | 2.564253 | 4.937628 | 2.615272 | high |
| TCGA-YF-AA3L | 2.606675 | 2.76934 | 2.497418 | 2.382946 | 2.495679 | 3.176865 | 2.676478 | low |
| TCGA-CF-A3MI | 2.629924 | 2.76934 | 2.497418 | 2.667481 | 2.434226 | 3.222882 | 2.647799 | low |
| TCGA-DK-A6B0 | 2.597324 | 2.76934 | 2.497418 | 2.42059 | 2.434226 | 3.199033 | 2.615272 | low |
| TCGA-DK-A6B1 | 3.831588 | 2.880507 | 2.497418 | 3.113522 | 2.434226 | 3.176865 | 2.748762 | low |
| TCGA-G2-AA3C | 2.597324 | 2.806526 | 2.497418 | 2.662236 | 2.517946 | 12.293386 | 2.862473 | high |
| TCGA-XF-AAN3 | 2.603989 | 2.76934 | 5.788218 | 4.484128 | 2.627646 | 3.205265 | 2.689677 | high |
| TCGA-CU-A0YO | 2.687502 | 2.76934 | 2.497418 | 2.864512 | 3.711176 | 3.176865 | 3.947997 | high |
| TCGA-BT-A3PJ | 3.734652 | 2.936531 | 2.497418 | 4.539702 | 5.54178 | 3.176865 | 2.824455 | high |
| TCGA-CF-A47Y | 2.619607 | 2.76934 | 2.497418 | 2.382946 | 2.434226 | 3.176865 | 2.615272 | low |
| TCGA-FD-A62O | 2.602007 | 2.801964 | 2.497418 | 4.406247 | 2.459249 | 3.20332 | 2.974133 | low |
| TCGA-BL-A0C8 | 3.318568 | 2.76934 | 2.497418 | 2.779548 | 2.442656 | 3.197935 | 2.645703 | low |
| TCGA-E7-A7DV | 2.60059 | 2.76934 | 2.633314 | 2.382946 | 4.796854 | 6.650431 | 2.624179 | high |
| TCGA-DK-A3WY | 2.621983 | 2.934295 | 2.510049 | 2.382946 | 2.445315 | 3.376871 | 2.615272 | low |
| TCGA-BT-A20N | 3.164325 | 2.806982 | 2.593799 | 3.360959 | 3.195607 | 3.176865 | 3.305848 | high |
| TCGA-G2-A2ES | 2.945982 | 2.835857 | 2.583831 | 11.978168 | 4.17159 | 3.176865 | 3.156971 | high |
| TCGA-GC-A3YS | 2.627282 | 2.799373 | 2.835747 | 2.612665 | 2.892823 | 3.225193 | 3.284329 | high |
| TCGA-FD-A43X | 2.600873 | 2.76934 | 2.497418 | 2.382946 | 2.434226 | 3.294049 | 2.689195 | low |
| TCGA-XF-A9SM | 2.644848 | 3.233395 | 2.715255 | 4.652393 | 3.756531 | 3.333982 | 3.713449 | high |
| TCGA-DK-A6AV | 2.632283 | 2.810166 | 2.497418 | 2.382946 | 2.598126 | 3.306096 | 2.631008 | low |
| TCGA-GU-A42Q | 3.558102 | 2.794025 | 8.91209 | 4.63183 | 2.587252 | 3.176865 | 4.339014 | high |
| TCGA-C4-A0F7 | 8.39035 | 2.76934 | 2.497418 | 6.539059 | 2.585917 | 3.191606 | 11.152529 | high |
| TCGA-CF-A47W | 2.597324 | 2.76934 | 2.497418 | 2.382946 | 2.434226 | 3.197472 | 2.625066 | low |
| TCGA-XF-AAMQ | 2.602432 | 2.76934 | 2.497418 | 2.951325 | 2.601491 | 3.176865 | 2.744961 | low |
| TCGA-K4-A3WS | 2.754085 | 2.76934 | 2.562644 | 2.715661 | 2.480148 | 3.249491 | 3.380994 | low |
| TCGA-C4-A0EZ | 2.680549 | 7.619665 | 2.521105 | 5.745263 | 2.698868 | 3.385408 | 6.832573 | high |
| TCGA-CF-A47S | 2.60144 | 2.78389 | 2.497418 | 3.054504 | 2.439782 | 3.176865 | 2.615272 | low |
| TCGA-ZF-AA5P | 2.630201 | 2.802269 | 2.497418 | 3.153031 | 2.781234 | 3.331977 | 2.736511 | low |
| TCGA-XF-A9SX | 2.597324 | 4.721743 | 2.576339 | 2.382946 | 2.503572 | 3.250442 | 2.765559 | high |
| TCGA-FD-A3B8 | 2.610905 | 2.793107 | 2.497418 | 2.978669 | 3.440636 | 3.289396 | 2.769752 | high |
| TCGA-CF-A27C | 2.614279 | 2.76934 | 2.507907 | 2.382946 | 2.479361 | 3.176865 | 2.633403 | low |
| TCGA-XF-A9T4 | 2.743352 | 2.892047 | 2.50536 | 5.898599 | 4.873682 | 3.191606 | 3.291116 | high |
| TCGA-HQ-A2OF | 2.601582 | 2.798611 | 2.522961 | 2.42328 | 2.434226 | 3.176865 | 2.637676 | low |
| TCGA-DK-AA74 | 2.619746 | 2.76934 | 2.670638 | 6.441182 | 2.657691 | 3.2603 | 4.172447 | high |
| TCGA-FD-A3SM | 2.60865 | 2.969421 | 2.751863 | 2.52271 | 2.570162 | 3.198252 | 2.615272 | low |
| TCGA-KQ-A41P | 2.60611 | 3.196851 | 2.497418 | 3.008048 | 2.91492 | 3.320455 | 3.01493 | high |
| TCGA-C4-A0F6 | 5.338694 | 2.76934 | 2.497418 | 2.510801 | 3.063187 | 3.215738 | 9.080917 | high |
| TCGA-FD-A3B5 | 8.881939 | 6.893058 | 2.605314 | 10.282227 | 7.359223 | 3.228079 | 8.668023 | high |
| TCGA-4Z-AA80 | 2.799188 | 2.76934 | 2.497418 | 2.616444 | 2.448274 | 3.206432 | 2.670622 | low |
| TCGA-FD-A3B6 | 2.635193 | 4.417543 | 2.599318 | 2.896135 | 4.358243 | 3.282489 | 2.665599 | high |
| TCGA-4Z-AA87 | 2.940522 | 2.76934 | 2.649936 | 3.31922 | 2.434226 | 3.2603 | 2.615272 | low |
| TCGA-DK-A1A6 | 2.765496 | 2.76934 | 2.497418 | 4.0222 | 2.451898 | 3.213803 | 2.615272 | low |
| TCGA-DK-A1AD | 2.672887 | 3.184397 | 2.611283 | 3.940036 | 2.434226 | 3.195127 | 3.636445 | high |
| TCGA-FD-A5BS | 2.597324 | 2.76934 | 3.307886 | 2.382946 | 2.630704 | 3.504558 | 2.696884 | high |
| TCGA-2F-A9KW | 2.62756 | 2.804551 | 2.512855 | 2.763281 | 2.461247 | 3.233454 | 2.642185 | low |
| TCGA-DK-AA6T | 2.597324 | 2.803487 | 2.497418 | 2.429737 | 2.572003 | 3.176865 | 2.628357 | low |
| TCGA-4Z-AA84 | 2.606957 | 2.802726 | 2.555292 | 2.636509 | 2.521897 | 3.176865 | 2.615272 | low |
| TCGA-FJ-A3Z9 | 5.071625 | 2.843734 | 2.497418 | 5.798549 | 4.737528 | 3.207403 | 3.113981 | high |
| TCGA-CU-A0YR | 2.720467 | 2.837048 | 2.512454 | 2.382946 | 2.447468 | 3.337804 | 2.667072 | low |
| TCGA-FD-A3B3 | 5.293271 | 3.433436 | 2.513389 | 8.785114 | 3.171604 | 3.23537 | 9.147857 | high |
| TCGA-FD-A43N | 2.998925 | 3.079474 | 2.507371 | 4.941933 | 2.598968 | 3.231536 | 2.910182 | low |
| TCGA-BL-A13I | 2.597324 | 2.76934 | 9.724249 | 2.622472 | 2.434226 | 3.283394 | 3.805163 | high |
| TCGA-KQ-A41S | 2.601299 | 2.76934 | 2.497418 | 2.74477 | 2.495939 | 3.176865 | 2.666704 | low |
| TCGA-4Z-AA86 | 2.6588 | 3.608389 | 2.746485 | 6.322202 | 4.636446 | 3.33835 | 4.264232 | high |
| TCGA-E7-A5KF | 2.666944 | 2.796778 | 2.497418 | 2.420835 | 2.434226 | 3.264456 | 2.615272 | low |
| TCGA-XF-AAN7 | 2.631867 | 2.76934 | 2.52402 | 2.382946 | 2.502927 | 3.273305 | 2.661172 | low |
| TCGA-DK-AA6M | 2.628951 | 2.897928 | 2.573407 | 2.708472 | 2.739375 | 3.247779 | 3.501518 | high |
| TCGA-CU-A3YL | 2.662742 | 2.798305 | 2.57213 | 2.382946 | 2.521133 | 3.200398 | 2.701786 | low |
| TCGA-ZF-A9RD | 3.551652 | 6.717628 | 2.682343 | 10.948204 | 6.084738 | 3.222111 | 7.803016 | high |
| TCGA-C4-A0F0 | 2.84075 | 2.76934 | 2.497418 | 3.135724 | 5.176015 | 3.176865 | 3.358718 | high |
| TCGA-FD-A3SR | 2.605545 | 2.826291 | 2.510049 | 3.347003 | 2.714421 | 3.200203 | 2.771448 | low |
| TCGA-DK-A6B5 | 2.765622 | 2.883111 | 2.517917 | 4.25014 | 3.190404 | 3.195909 | 4.599789 | high |
| TCGA-HQ-A5ND | 2.72164 | 2.76934 | 2.497418 | 3.474928 | 2.549241 | 4.399957 | 2.627219 | high |
| TCGA-DK-AA76 | 2.868972 | 3.003771 | 2.497418 | 4.394454 | 3.886956 | 3.176865 | 3.120961 | high |
| TCGA-CF-A47V | 2.608932 | 2.76934 | 2.509246 | 2.382946 | 2.434226 | 3.176865 | 2.79795 | low |
| TCGA-E7-A3Y1 | 2.693625 | 2.76934 | 2.497418 | 2.467918 | 2.446257 | 3.202346 | 3.166958 | low |
| TCGA-ZF-AA53 | 4.853113 | 2.958305 | 3.215941 | 6.822361 | 2.808406 | 3.332707 | 5.379113 | high |
| TCGA-ZF-A9R7 | 3.056258 | 2.76934 | 2.787396 | 2.561832 | 2.830591 | 3.213803 | 2.981055 | high |
| TCGA-GV-A40G | 2.60059 | 3.078965 | 2.497418 | 2.808449 | 2.468813 | 3.195127 | 2.666458 | low |
| TCGA-4Z-AA7M | 2.804727 | 2.806071 | 2.513655 | 3.191804 | 2.462444 | 3.176865 | 2.629368 | low |
| TCGA-BT-A2LD | 7.677542 | 5.043586 | 2.497418 | 6.908489 | 5.569821 | 3.421113 | 3.057718 | high |
| TCGA-4Z-AA82 | 2.673156 | 2.76934 | 2.552444 | 2.858731 | 4.248921 | 3.228079 | 3.180153 | high |
| TCGA-FD-A62P | 2.927061 | 2.76934 | 2.676917 | 5.579798 | 4.368931 | 3.263512 | 3.356862 | high |
| TCGA-DK-AA6U | 2.986765 | 2.795096 | 2.508711 | 3.984942 | 2.473306 | 3.218251 | 3.353213 | low |
| TCGA-FD-A3N6 | 10.508281 | 9.067884 | 2.648247 | 10.897095 | 6.568322 | 3.318804 | 11.731332 | high |
| TCGA-2F-A9KR | 2.983952 | 2.76934 | 2.511386 | 5.470087 | 2.617719 | 3.202541 | 8.457513 | high |
| TCGA-CF-A3MF | 2.601724 | 2.76934 | 2.497418 | 2.464612 | 2.445853 | 3.176865 | 2.626839 | low |
| TCGA-CU-A3KJ | 2.90388 | 2.76934 | 2.497418 | 4.777551 | 3.603137 | 3.387714 | 2.886757 | high |
| TCGA-FD-A6TC | 2.663014 | 2.901932 | 2.874792 | 2.626152 | 3.275292 | 3.199033 | 2.706907 | high |
| TCGA-XF-A8HB | 2.604272 | 2.793413 | 2.508041 | 2.479886 | 3.672896 | 3.176865 | 2.651776 | low |
| TCGA-XF-AAN1 | 2.603989 | 2.815459 | 2.497418 | 2.817685 | 2.452032 | 3.214384 | 2.667807 | low |
| TCGA-FJ-A871 | 2.597324 | 2.898357 | 2.520707 | 2.382946 | 2.434226 | 15.923566 | 2.645681 | high |
| TCGA-UY-A9PA | 2.609073 | 2.76934 | 2.567396 | 2.491973 | 2.704443 | 3.209733 | 2.895707 | low |
| TCGA-E7-A4IJ | 2.777818 | 2.76934 | 2.513655 | 2.482569 | 2.434226 | 3.176865 | 2.629494 | low |
| TCGA-XF-AAMZ | 2.653482 | 2.76934 | 2.497418 | 2.382946 | 2.434226 | 3.176865 | 2.835679 | low |
| TCGA-XF-AAMR | 2.605121 | 2.76934 | 2.846736 | 2.741453 | 2.449752 | 3.198643 | 2.889953 | low |
| TCGA-BT-A42E | 4.345019 | 2.76934 | 2.701049 | 4.138401 | 2.815192 | 3.176865 | 9.221881 | high |
| TCGA-K4-A6FZ | 3.985538 | 2.76934 | 2.537324 | 9.464154 | 4.077741 | 3.176865 | 8.898526 | high |
| TCGA-DK-A1AF | 2.614841 | 2.980322 | 2.515255 | 5.80589 | 2.671555 | 3.723225 | 3.076986 | high |
| TCGA-FD-A6TB | 2.597324 | 2.797847 | 2.509915 | 2.532941 | 2.69383 | 3.200008 | 2.637048 | low |
| TCGA-GU-AATO | 2.603989 | 6.424684 | 2.497418 | 2.382946 | 2.57139 | 3.287718 | 2.767713 | high |
| TCGA-BT-A20U | 3.915771 | 2.814402 | 3.931581 | 7.074633 | 3.526869 | 3.284358 | 9.446188 | high |
| TCGA-DK-A1AA | 2.730468 | 2.76934 | 2.568293 | 2.68844 | 2.442214 | 3.22654 | 2.764877 | low |
| TCGA-GV-A3JW | 4.420928 | 3.009511 | 2.497418 | 3.417011 | 2.635395 | 3.195909 | 5.48633 | high |
| TCGA-FD-A5BZ | 2.699193 | 3.040124 | 3.610698 | 4.025975 | 4.511297 | 3.176865 | 4.959422 | high |
| TCGA-ZF-A9R9 | 3.299952 | 2.813495 | 2.536013 | 2.382946 | 2.501119 | 3.247779 | 2.697962 | low |
| TCGA-G2-AA3F | 4.776856 | 2.76934 | 2.497418 | 3.689414 | 2.434226 | 3.353019 | 2.739643 | high |
| TCGA-ZF-AA5H | 2.603282 | 4.085591 | 2.515388 | 2.894371 | 6.492271 | 3.209927 | 2.661788 | high |
| TCGA-XF-A8HD | 2.604131 | 2.792953 | 2.681283 | 2.382946 | 2.98013 | 3.251961 | 2.624306 | low |
| TCGA-FD-A6TH | 3.092632 | 2.959544 | 2.532334 | 4.378618 | 3.503597 | 3.256893 | 2.841147 | high |
| TCGA-4Z-AA7O | 2.817323 | 2.854666 | 2.497418 | 5.529256 | 3.534196 | 3.176865 | 6.062265 | high |
| TCGA-DK-A6B6 | 2.866033 | 2.76934 | 2.530491 | 2.434952 | 2.434226 | 3.237858 | 2.629873 | low |
| TCGA-E7-A6MF | 2.674772 | 2.76934 | 2.497418 | 2.382946 | 3.12482 | 3.176865 | 2.626966 | low |
| TCGA-DK-AA6W | 2.841109 | 6.098722 | 2.512988 | 6.230358 | 4.588296 | 3.233837 | 4.366913 | high |
| TCGA-GC-A3BM | 3.294218 | 2.797542 | 2.509781 | 2.459519 | 2.508466 | 3.176865 | 5.975861 | high |
| TCGA-GU-A42P | 3.659185 | 2.831829 | 2.497418 | 4.260455 | 2.474361 | 3.210896 | 2.655246 | low |
| TCGA-GV-A3QI | 5.373225 | 2.76934 | 2.506701 | 2.382946 | 2.450557 | 3.176865 | 2.768846 | high |
| TCGA-XF-A9T3 | 2.597324 | 6.406043 | 2.619695 | 2.382946 | 7.13503 | 3.176865 | 2.669889 | high |
| TCGA-E7-A8O7 | 2.604131 | 2.76934 | 2.497418 | 2.824989 | 2.46138 | 3.196104 | 2.624306 | low |
| TCGA-DK-AA77 | 2.602007 | 2.925177 | 2.497418 | 2.382946 | 2.932624 | 3.203125 | 2.779333 | low |
| TCGA-XF-AAML | 2.986549 | 2.76934 | 2.497418 | 5.248843 | 2.451227 | 3.247779 | 2.76044 | low |
| TCGA-2F-A9KQ | 2.703685 | 2.76934 | 2.497418 | 2.46272 | 2.543491 | 3.176865 | 2.754843 | low |
| TCGA-CF-A3MH | 2.628117 | 2.76934 | 2.507907 | 2.382946 | 2.566595 | 3.1963 | 2.624433 | low |
| TCGA-XF-A8HC | 3.458766 | 2.807589 | 2.497418 | 3.732009 | 2.463508 | 3.176865 | 2.644558 | low |
| TCGA-ZF-AA4T | 2.617646 | 2.76934 | 2.512988 | 2.382946 | 2.434226 | 3.176865 | 2.628862 | low |
| TCGA-FD-A6TI | 2.991404 | 3.641934 | 2.497418 | 2.382946 | 2.539853 | 3.212253 | 2.623672 | low |
| TCGA-H4-A2HO | 2.823519 | 2.800288 | 2.497418 | 2.544976 | 2.434226 | 3.22654 | 2.782473 | low |
| TCGA-CF-A47X | 2.651841 | 2.76934 | 2.497418 | 2.382946 | 2.442079 | 3.176865 | 2.623037 | low |
| TCGA-XF-A9SP | 2.621704 | 7.220015 | 2.497418 | 2.600731 | 3.673278 | 3.258219 | 2.615272 | high |
| TCGA-K4-A4AC | 4.076448 | 2.76934 | 2.497418 | 5.27634 | 3.934955 | 3.176865 | 9.661293 | high |
| TCGA-BL-A5ZZ | 4.200416 | 4.645874 | 2.509648 | 3.462452 | 5.431942 | 4.098768 | 4.074202 | high |
| TCGA-S5-AA26 | 2.639614 | 2.76934 | 2.497418 | 3.519184 | 2.754902 | 3.176865 | 6.023216 | high |
| TCGA-E7-A677 | 2.607945 | 2.806071 | 2.505494 | 3.204359 | 2.496717 | 3.176865 | 2.955487 | low |
| TCGA-DK-A1AC | 3.643 | 2.76934 | 2.497418 | 2.414332 | 2.434226 | 3.249491 | 2.623926 | low |
| TCGA-GU-A766 | 2.691765 | 4.961128 | 2.497418 | 8.776012 | 3.611093 | 3.538517 | 4.883463 | high |
| TCGA-DK-A2HX | 2.600306 | 3.276974 | 2.515388 | 3.08869 | 3.819448 | 3.242634 | 2.785047 | high |
| TCGA-UY-A8OC | 2.616104 | 2.865814 | 2.497418 | 5.501407 | 3.022602 | 3.229616 | 2.689074 | low |
| TCGA-FD-A5BR | 2.717074 | 2.76934 | 2.512588 | 3.684346 | 3.194056 | 3.313288 | 4.469951 | high |
| TCGA-DK-AA6S | 2.648966 | 3.12937 | 2.719613 | 2.52733 | 2.618549 | 3.23537 | 4.087615 | high |
| TCGA-4Z-AA7Q | 2.792266 | 2.76934 | 2.514189 | 2.667984 | 3.527537 | 3.297392 | 2.658583 | high |
| TCGA-HQ-A2OE | 2.662607 | 2.801355 | 2.497418 | 2.588684 | 2.434226 | 3.176865 | 3.084171 | low |
| TCGA-XF-AAMX | 3.027775 | 2.76934 | 2.723045 | 2.52969 | 2.517563 | 3.20682 | 2.657471 | low |
| TCGA-LT-A5Z6 | 2.627699 | 3.148376 | 2.497418 | 3.245624 | 2.91368 | 3.20546 | 4.619501 | high |
| TCGA-YC-A89H | 2.993772 | 2.76934 | 2.622282 | 3.0199 | 5.231704 | 3.224616 | 2.954117 | high |
| TCGA-E7-A8O8 | 2.662742 | 2.889458 | 2.497418 | 3.734646 | 2.526599 | 3.176865 | 2.761465 | low |
| TCGA-GC-A3OO | 2.619047 | 2.76934 | 2.497418 | 2.504518 | 2.962947 | 3.365788 | 3.440505 | high |
| TCGA-DK-AA75 | 4.159857 | 2.76934 | 2.497418 | 4.769689 | 2.455244 | 3.306096 | 3.114327 | high |
| TCGA-BT-A2LB | 2.661928 | 2.76934 | 2.685988 | 4.064952 | 2.797333 | 3.195518 | 3.67878 | high |
| TCGA-ZF-AA54 | 2.621844 | 2.803639 | 2.497418 | 2.382946 | 2.447468 | 3.204682 | 2.729058 | low |
| TCGA-FT-A3EE | 2.769027 | 4.305388 | 2.497418 | 2.470509 | 6.473748 | 3.176865 | 2.664248 | high |
| TCGA-FJ-A3ZF | 5.592809 | 2.788352 | 2.505762 | 2.409155 | 2.434226 | 3.176865 | 2.636923 | high |
| TCGA-DK-A3X2 | 6.892554 | 3.148864 | 2.509112 | 4.299155 | 3.257046 | 3.176865 | 8.548702 | high |
| TCGA-XF-AAME | 2.939964 | 3.226804 | 2.520441 | 8.673055 | 4.532133 | 3.240152 | 4.104116 | high |
| TCGA-ZF-AA4R | 2.636714 | 2.84714 | 3.104935 | 5.333081 | 3.179234 | 3.176865 | 5.882551 | high |
| TCGA-GV-A3JZ | 2.663149 | 2.76934 | 2.497418 | 2.382946 | 2.47291 | 4.991119 | 2.630756 | high |
| TCGA-DK-A2I2 | 3.165469 | 4.113688 | 2.497418 | 8.873302 | 7.307799 | 3.490641 | 7.32866 | high |
| TCGA-K4-A83P | 2.654574 | 2.76934 | 2.589518 | 2.456311 | 2.434226 | 3.283798 | 2.656235 | low |
| TCGA-4Z-AA7Y | 2.606533 | 2.76934 | 2.525343 | 2.830573 | 2.619142 | 3.176865 | 2.6988 | low |
| TCGA-E7-A7XN | 2.838352 | 2.76934 | 2.497418 | 7.270049 | 2.920341 | 3.201762 | 5.282828 | high |
| TCGA-2F-A9KP | 2.861083 | 2.76934 | 2.497418 | 2.569143 | 2.434226 | 3.200203 | 3.573027 | low |
| TCGA-UY-A78M | 2.698664 | 2.76934 | 3.860242 | 4.815683 | 2.521515 | 3.336531 | 4.987441 | high |
| TCGA-FD-A6TD | 2.601724 | 2.859223 | 2.497418 | 3.822734 | 3.178292 | 3.201567 | 2.727772 | low |
| TCGA-BT-A20Q | 2.601724 | 2.76934 | 2.777997 | 2.382946 | 2.537086 | 3.201762 | 2.627092 | low |
| TCGA-ZF-AA51 | 2.641407 | 2.76934 | 2.64583 | 2.660517 | 2.434226 | 3.316416 | 2.82033 | low |
| TCGA-GU-A42R | 2.973191 | 2.76934 | 2.497418 | 2.529914 | 2.59656 | 3.176865 | 7.051611 | high |
| TCGA-PQ-A6FI | 3.244975 | 2.812739 | 2.506969 | 9.26151 | 2.707888 | 3.176865 | 6.657306 | high |
| TCGA-ZF-AA4X | 2.631173 | 2.76934 | 2.497418 | 3.878306 | 2.501377 | 3.200983 | 2.671234 | low |
| TCGA-FD-A5BU | 5.431126 | 2.802726 | 2.597063 | 9.086048 | 3.562466 | 3.203904 | 6.713034 | high |
| TCGA-CF-A8HX | 2.632283 | 2.799983 | 2.510851 | 2.382946 | 2.434226 | 3.250442 | 2.909675 | low |
| TCGA-XF-A9SK | 2.618207 | 2.805767 | 6.020151 | 2.433014 | 2.886804 | 3.176865 | 2.926297 | high |
| TCGA-GD-A3OS | 2.647045 | 3.590974 | 2.580789 | 4.536688 | 2.766645 | 3.538517 | 2.990037 | high |
| TCGA-FD-A6TG | 2.611608 | 2.794331 | 2.508443 | 2.450826 | 2.481591 | 3.236901 | 2.843392 | low |
| TCGA-4Z-AA7N | 3.373613 | 2.76934 | 2.497418 | 2.735746 | 2.521388 | 3.48373 | 3.170434 | high |
| TCGA-GV-A3QK | 2.597324 | 2.76934 | 2.515655 | 2.546971 | 2.442214 | 3.243778 | 2.631261 | low |
| TCGA-K4-A5RI | 2.609637 | 2.76934 | 3.389929 | 5.846337 | 2.488523 | 3.176865 | 3.608008 | high |
| TCGA-GV-A40E | 2.604979 | 4.452551 | 2.865059 | 4.956224 | 3.284615 | 3.458921 | 2.713782 | high |
| TCGA-FD-A43Y | 3.141053 | 2.945999 | 4.35359 | 5.825754 | 2.980675 | 3.20682 | 5.631112 | high |
| TCGA-DK-A3IV | 2.615964 | 2.76934 | 2.634291 | 2.919755 | 2.825816 | 3.21206 | 2.640183 | low |
| TCGA-G2-A2EL | 2.758914 | 3.705312 | 2.497418 | 5.45286 | 2.596199 | 3.294049 | 7.078624 | high |
| TCGA-E7-A541 | 2.616945 | 2.803639 | 2.497418 | 2.382946 | 2.992607 | 3.204682 | 2.79983 | low |
| TCGA-XF-AAN2 | 3.657771 | 6.232737 | 2.518848 | 5.097461 | 5.380077 | 3.216318 | 4.877093 | high |
| TCGA-LC-A66R | 2.619467 | 4.219116 | 2.497418 | 6.786567 | 3.158366 | 3.376514 | 3.89624 | high |
| TCGA-ZF-A9RE | 2.921867 | 2.76934 | 2.497418 | 9.899787 | 2.658842 | 3.176865 | 3.463383 | high |
| TCGA-UY-A9PE | 2.679878 | 2.97188 | 2.520839 | 4.1588 | 2.553103 | 3.176865 | 4.222632 | high |
| TCGA-XF-AAMG | 3.325131 | 2.930514 | 2.526797 | 2.429129 | 2.434226 | 3.204098 | 2.714963 | low |
| TCGA-GU-A763 | 2.648006 | 2.790501 | 2.497418 | 2.382946 | 2.489957 | 3.210896 | 2.738832 | low |
| TCGA-G2-AA3D | 2.720206 | 2.866106 | 2.497418 | 4.034344 | 2.44693 | 3.229808 | 2.665108 | low |
| TCGA-ZF-A9R4 | 2.807427 | 2.76934 | 2.509781 | 2.724349 | 2.466295 | 3.176865 | 2.62608 | low |
| TCGA-E7-A97P | 2.710001 | 2.800593 | 2.538241 | 7.383692 | 2.942465 | 3.176865 | 2.615272 | low |
| TCGA-5N-A9KM | 2.597324 | 2.76934 | 2.997281 | 2.382946 | 2.635278 | 3.176865 | 2.627219 | high |
| TCGA-ZF-A9RC | 2.602999 | 2.808651 | 2.497418 | 2.632376 | 2.817037 | 3.27105 | 3.637019 | low |
| TCGA-MV-A51V | 2.862146 | 2.804703 | 2.497418 | 2.47825 | 2.46138 | 3.233645 | 2.615272 | low |
| TCGA-S5-A6DX | 2.614279 | 3.092668 | 2.497418 | 4.651434 | 2.464438 | 3.529793 | 4.56809 | high |
| TCGA-FJ-A3ZE | 2.798448 | 2.76934 | 2.497418 | 2.755515 | 2.502411 | 3.176865 | 4.048777 | low |
| TCGA-YC-A8S6 | 2.666267 | 2.813646 | 2.516853 | 2.443635 | 2.580807 | 3.447431 | 3.504831 | high |
| TCGA-UY-A9PF | 2.682959 | 2.76934 | 2.519778 | 2.452378 | 2.473174 | 3.176865 | 2.654008 | low |
| TCGA-GV-A3JV | 2.677059 | 2.76934 | 2.497418 | 2.414332 | 2.443023 | 3.176865 | 2.615272 | low |
| TCGA-ZF-AA5N | 8.952015 | 3.229004 | 2.562644 | 4.520538 | 9.505648 | 3.296464 | 3.034022 | high |
| TCGA-XF-A9T8 | 2.782691 | 2.76934 | 2.497418 | 11.033538 | 5.374342 | 3.287718 | 2.6988 | high |
| TCGA-5N-A9KI | 2.60144 | 2.76934 | 2.534306 | 2.921402 | 2.664698 | 3.266908 | 2.940233 | low |
| TCGA-CF-A3MG | 2.699987 | 2.802878 | 2.512187 | 2.516373 | 2.459915 | 3.176865 | 2.615272 | low |
| TCGA-GC-A4ZW | 2.626586 | 2.76934 | 2.512454 | 2.382946 | 2.434226 | 3.176865 | 2.641309 | low |
| TCGA-E7-A4XJ | 6.01062 | 3.25869 | 2.497418 | 3.308127 | 3.693783 | 3.176865 | 2.749107 | high |
| TCGA-FD-A3NA | 2.689235 | 2.942664 | 2.527193 | 6.287647 | 2.76707 | 3.35356 | 2.786835 | low |
| TCGA-DK-A3IT | 2.650883 | 2.796472 | 2.497418 | 2.491626 | 2.525203 | 3.176865 | 3.455207 | low |
| TCGA-R3-A69X | 2.730209 | 2.76934 | 2.509246 | 2.382946 | 2.485127 | 3.262379 | 2.91766 | low |
| TCGA-4Z-AA83 | 2.603282 | 2.76934 | 2.515655 | 2.691801 | 2.434226 | 3.210509 | 2.708214 | low |
| TCGA-ZF-A9R0 | 2.646771 | 2.76934 | 2.497418 | 2.48699 | 2.434226 | 3.269356 | 2.615272 | low |
| TCGA-GC-A3I6 | 2.75434 | 2.76934 | 2.556585 | 6.630375 | 3.47652 | 3.176865 | 6.01639 | high |
| TCGA-XF-A9SH | 2.597324 | 2.76934 | 3.050748 | 2.755421 | 2.45404 | 3.176865 | 2.654503 | high |
| TCGA-UY-A78L | 2.636299 | 3.718376 | 2.623267 | 8.820229 | 4.166065 | 3.197081 | 4.700065 | high |
| TCGA-XF-AAN5 | 2.668838 | 2.76934 | 2.797811 | 3.155425 | 5.06981 | 3.227887 | 2.686662 | high |
| TCGA-UY-A78P | 2.683628 | 2.830483 | 2.576721 | 5.637956 | 2.834135 | 3.644505 | 3.127304 | high |
| TCGA-CF-A8HY | 2.602291 | 2.76934 | 2.497418 | 2.759638 | 2.434226 | 3.176865 | 2.615272 | low |
| TCGA-CF-A9FL | 2.731115 | 2.76934 | 2.497418 | 2.427548 | 3.234566 | 3.203125 | 2.676235 | low |
| TCGA-E7-A6MD | 2.657711 | 2.76934 | 2.51192 | 2.471802 | 2.434226 | 3.229808 | 2.857202 | low |
| TCGA-CF-A9FF | 2.668026 | 2.76934 | 2.497418 | 3.843421 | 2.497495 | 3.176865 | 2.708689 | low |
| TCGA-XF-AAMY | 2.63989 | 2.819078 | 2.497418 | 3.536885 | 2.453371 | 3.176865 | 2.671845 | low |
| TCGA-G2-A3IE | 2.728266 | 2.76934 | 2.497418 | 3.018529 | 2.444102 | 3.197667 | 2.960174 | low |
| TCGA-GU-A767 | 2.754085 | 2.829287 | 2.497418 | 3.239373 | 2.649836 | 3.176865 | 3.245794 | low |
| TCGA-G2-A3IB | 7.994448 | 4.727626 | 4.584279 | 11.038869 | 6.46843 | 3.231536 | 11.525553 | high |
| TCGA-CF-A1HS | 6.648511 | 3.039602 | 2.497418 | 10.232502 | 3.476268 | 3.176865 | 10.953132 | high |
| TCGA-DK-A3IN | 3.746923 | 4.662166 | 2.497418 | 4.696797 | 2.987648 | 3.237667 | 2.699995 | high |
| TCGA-2F-A9KT | 2.769405 | 2.888594 | 2.515388 | 4.96672 | 4.09071 | 3.305911 | 6.286331 | high |
| TCGA-UY-A78N | 2.826184 | 2.76934 | 2.497418 | 2.579329 | 2.443967 | 3.217671 | 3.686584 | low |
| TCGA-DK-A1A5 | 2.615122 | 2.830782 | 2.731358 | 2.439421 | 2.924983 | 3.176865 | 2.906019 | low |
| TCGA-E7-A5KE | 2.646222 | 2.800746 | 2.497418 | 2.382946 | 2.539099 | 3.22731 | 2.63918 | low |
| TCGA-FD-A62S | 2.769153 | 3.583437 | 2.531149 | 4.57112 | 5.025738 | 3.239005 | 5.067333 | high |
| TCGA-G2-AA3B | 2.930326 | 2.812891 | 2.497418 | 5.207158 | 2.434226 | 3.176865 | 2.632017 | low |
| TCGA-K4-A54R | 2.740142 | 2.76934 | 2.580027 | 4.046069 | 2.666759 | 3.328144 | 2.644683 | low |
| TCGA-XF-A9T5 | 2.636438 | 2.76934 | 2.497418 | 2.642272 | 2.434226 | 3.258976 | 3.190855 | low |
| TCGA-BT-A20J | 2.757518 | 2.805919 | 2.513522 | 2.382946 | 2.434226 | 3.481196 | 2.859207 | low |
| TCGA-XF-AAMW | 2.686835 | 3.274504 | 2.518981 | 6.1986 | 3.836425 | 3.176865 | 2.706788 | high |
| TCGA-ZF-AA4V | 2.60865 | 5.199053 | 2.553998 | 4.080342 | 5.822765 | 3.198057 | 3.123794 | high |
| TCGA-GU-AATP | 2.696412 | 2.929813 | 4.461826 | 2.440145 | 2.698085 | 3.210703 | 2.647176 | high |
| TCGA-CU-A0YN | 6.067414 | 5.805109 | 2.521635 | 10.764197 | 6.730859 | 3.307758 | 8.955591 | high |
| TCGA-XF-AAMH | 2.668973 | 6.899648 | 2.716747 | 5.517001 | 6.43805 | 3.886887 | 3.043096 | high |
| TCGA-DK-A2I6 | 2.624496 | 2.76934 | 2.505762 | 2.409278 | 3.735554 | 3.176865 | 2.672577 | high |
| TCGA-DK-AA6Q | 2.654711 | 2.842251 | 9.980024 | 7.247704 | 2.795773 | 3.20682 | 3.886415 | high |
| TCGA-XF-A8HI | 2.602007 | 2.80166 | 2.525608 | 2.590717 | 2.446661 | 3.254239 | 2.821743 | low |
| TCGA-DK-A2I1 | 2.777818 | 2.76934 | 2.693598 | 3.206461 | 2.453505 | 3.315496 | 2.812585 | low |
| TCGA-E7-A7PW | 3.025463 | 2.845216 | 2.531018 | 3.189877 | 2.548368 | 3.327779 | 5.690475 | high |
| TCGA-XF-AAMT | 2.867562 | 2.918268 | 3.057809 | 3.084141 | 2.922996 | 3.218444 | 3.867207 | high |
| TCGA-BT-A20R | 3.164992 | 2.795861 | 2.497418 | 2.382946 | 2.570285 | 3.240343 | 2.615272 | low |
| TCGA-H4-A2HQ | 2.663014 | 2.76934 | 2.497418 | 2.467092 | 2.48146 | 3.176865 | 3.089995 | low |
| TCGA-FD-A43S | 2.610341 | 2.76934 | 2.497418 | 2.464731 | 2.457382 | 3.225578 | 3.334957 | low |
| TCGA-ZF-AA58 | 2.597324 | 2.92869 | 2.533649 | 2.458094 | 2.52736 | 3.370264 | 2.707857 | low |
| TCGA-DK-AA6P | 2.76701 | 2.76934 | 2.497418 | 2.382946 | 2.434226 | 3.176865 | 2.615272 | low |
| TCGA-XF-AAN0 | 2.603706 | 2.76934 | 2.573152 | 3.34847 | 2.57971 | 3.247589 | 2.657101 | low |
| TCGA-BT-A0YX | 2.837272 | 2.76934 | 2.575192 | 8.650011 | 2.826935 | 3.201372 | 2.829108 | low |
| TCGA-ZF-A9R5 | 2.624496 | 2.76934 | 2.497418 | 2.944799 | 2.434226 | 3.176865 | 2.627345 | low |
| TCGA-GV-A3JX | 2.738856 | 9.016366 | 2.516321 | 6.42605 | 2.704665 | 3.32851 | 6.629279 | high |
| TCGA-ZF-A9R3 | 2.641545 | 2.76934 | 2.497418 | 2.729342 | 2.463906 | 3.176865 | 3.335974 | low |
| TCGA-G2-A2EO | 2.666403 | 2.76934 | 2.510851 | 2.719916 | 2.682908 | 3.201762 | 2.627092 | low |
| TCGA-CF-A5UA | 2.665726 | 2.76934 | 2.497418 | 2.504289 | 2.468945 | 3.176865 | 2.615272 | low |
| TCGA-K4-A3WU | 3.129957 | 2.852014 | 2.524946 | 4.538584 | 2.602211 | 3.193954 | 2.82413 | low |
| TCGA-DK-AA71 | 2.731115 | 2.76934 | 2.497418 | 2.673304 | 3.734877 | 3.176865 | 6.077874 | high |
| TCGA-XF-A9T2 | 2.738727 | 11.742504 | 2.523888 | 2.382946 | 2.650415 | 6.542568 | 3.316584 | high |
| TCGA-FD-A5BY | 2.680683 | 2.76934 | 2.598567 | 3.579983 | 6.979911 | 3.224808 | 3.492364 | high |
| TCGA-BT-A20T | 2.661248 | 2.76934 | 2.54112 | 4.203914 | 2.986112 | 3.217478 | 6.201332 | high |
| TCGA-UY-A78O | 2.803867 | 2.76934 | 2.497418 | 2.427061 | 2.434226 | 3.20293 | 2.675626 | low |
| TCGA-4Z-AA89 | 2.732279 | 2.76934 | 2.497418 | 3.286383 | 2.477389 | 3.176865 | 2.877432 | low |
| TCGA-XF-A9SW | 2.597324 | 2.829736 | 2.649816 | 4.851272 | 2.651341 | 3.273681 | 2.769865 | low |
| TCGA-BT-A20W | 2.6907 | 2.810923 | 2.551666 | 2.494746 | 2.466163 | 3.176865 | 3.685204 | low |
| TCGA-FD-A3N5 | 9.457213 | 4.308223 | 2.497418 | 10.577479 | 8.114094 | 3.208762 | 13.710479 | high |
| TCGA-DK-A3WX | 5.019425 | 4.590459 | 2.609048 | 6.410817 | 4.55465 | 3.240343 | 5.950933 | high |
| TCGA-FD-A62N | 2.597324 | 2.895062 | 2.511653 | 2.629164 | 2.940596 | 3.304617 | 2.627725 | low |
| TCGA-HQ-A5NE | 2.636991 | 3.001227 | 2.510985 | 7.558998 | 4.269176 | 3.251012 | 3.481718 | high |
| TCGA-XF-A8HE | 2.769783 | 2.884411 | 2.548679 | 10.422887 | 5.300347 | 3.200788 | 4.545141 | high |
| TCGA-DK-AA6R | 2.985359 | 2.76934 | 2.497418 | 10.735197 | 5.8069 | 3.176865 | 4.111812 | high |
| TCGA-XF-A9SI | 2.683361 | 2.80166 | 2.52574 | 2.427426 | 2.446661 | 3.228848 | 2.627725 | low |
| TCGA-BT-A42C | 2.815252 | 2.813344 | 2.507103 | 2.821609 | 2.476205 | 3.264833 | 4.296159 | high |
| TCGA-KQ-A41O | 2.611749 | 2.76934 | 2.526797 | 2.382946 | 2.746513 | 3.176865 | 2.653636 | low |
| TCGA-YF-AA3M | 2.611608 | 2.866837 | 2.497418 | 3.024078 | 2.453371 | 3.176865 | 3.445913 | low |
| TCGA-ZF-AA4N | 2.746555 | 2.909624 | 9.105513 | 5.420419 | 2.630117 | 3.176865 | 5.929744 | high |
| TCGA-G2-A2EC | 2.618207 | 2.793719 | 2.497418 | 3.130228 | 2.507437 | 3.176865 | 2.633906 | low |
| TCGA-CF-A5U8 | 2.602007 | 2.76934 | 2.497418 | 2.382946 | 2.434226 | 3.176865 | 2.615272 | low |
| TCGA-UY-A8OD | 2.597324 | 2.76934 | 2.512187 | 2.473681 | 3.270901 | 3.204098 | 2.944582 | low |
| TCGA-4Z-AA7R | 2.60611 | 2.799831 | 2.497418 | 4.048058 | 2.445988 | 3.225963 | 2.706194 | low |
| TCGA-FD-A3B4 | 3.600553 | 4.324842 | 2.797703 | 7.943492 | 5.376101 | 3.241107 | 6.332517 | high |
| TCGA-FJ-A3Z7 | 2.832462 | 2.76934 | 2.497418 | 2.55084 | 2.434226 | 3.258786 | 2.66388 | low |
| TCGA-G2-A2EJ | 4.310522 | 8.102987 | 2.497418 | 11.293537 | 8.40683 | 3.176865 | 4.519647 | high |
| TCGA-SY-A9G0 | 2.739628 | 2.76934 | 2.515655 | 2.494054 | 2.434226 | 3.210315 | 2.631134 | low |
| TCGA-G2-A2EK | 2.628951 | 2.813344 | 2.516853 | 2.382946 | 2.434226 | 3.176865 | 3.242516 | low |
| TCGA-FD-A5BX | 2.597324 | 2.76934 | 2.497418 | 2.382946 | 2.434226 | 3.371516 | 2.615272 | low |
| TCGA-XF-AAN4 | 2.609637 | 2.893627 | 2.497418 | 2.382946 | 2.560299 | 3.437257 | 2.663757 | low |
| TCGA-FD-A3SO | 2.65102 | 2.798458 | 2.522961 | 3.899662 | 2.88972 | 3.292189 | 2.691603 | low |
| TCGA-FD-A6TA | 2.607522 | 2.76934 | 3.226525 | 3.026579 | 2.624935 | 3.261434 | 2.655493 | high |
| TCGA-FD-A3SS | 5.780272 | 4.606146 | 2.618832 | 3.65576 | 6.6448 | 3.212641 | 8.561921 | high |
| TCGA-FD-A43P | 2.693493 | 2.898644 | 2.555163 | 2.88881 | 2.442754 | 3.316783 | 2.729992 | low |
| TCGA-BT-A3PK | 2.781818 | 2.958856 | 2.504151 | 9.625485 | 3.834586 | 3.3674 | 2.717559 | high |
| TCGA-XF-A9T6 | 2.636438 | 7.886782 | 2.497418 | 2.602852 | 3.728611 | 3.176865 | 2.704766 | high |
| TCGA-FD-A5BV | 5.593309 | 2.76934 | 2.497418 | 2.55997 | 5.443773 | 3.176865 | 2.7634 | high |
| TCGA-ZF-AA4U | 2.621564 | 2.76934 | 2.497418 | 2.676806 | 2.447199 | 3.204293 | 4.723602 | high |
| TCGA-SY-A9G5 | 2.64361 | 2.76934 | 3.313651 | 2.526655 | 2.71232 | 3.263701 | 2.656483 | high |
| TCGA-KQ-A41R | 2.799435 | 2.901789 | 2.497418 | 3.326581 | 2.434226 | 3.176865 | 2.68376 | low |
| TCGA-GC-A6I1 | 2.865798 | 2.864937 | 2.525872 | 3.835434 | 3.540763 | 3.203125 | 3.713882 | high |
| TCGA-GU-A764 | 2.673156 | 5.259638 | 2.665164 | 3.392444 | 4.560221 | 3.176865 | 2.771561 | high |
| TCGA-G2-A2EF | 2.769783 | 2.82344 | 2.497418 | 3.739817 | 2.981311 | 3.206237 | 4.357812 | high |
| TCGA-FT-A61P | 2.620166 | 5.109578 | 2.525343 | 2.426817 | 3.526747 | 3.327048 | 2.687024 | high |
| TCGA-K4-AAQO | 2.981459 | 2.804095 | 2.512721 | 2.520903 | 2.434226 | 3.176865 | 6.233826 | high |
| TCGA-DK-AA6L | 2.597324 | 2.76934 | 2.594427 | 2.532941 | 3.754068 | 3.176865 | 2.68642 | high |
| TCGA-K4-A6MB | 5.904727 | 3.599933 | 2.508041 | 6.074762 | 2.704331 | 3.234987 | 8.500511 | high |
| TCGA-BT-A20P | 2.597324 | 2.821035 | 2.520176 | 2.45381 | 2.434226 | 3.176865 | 2.615272 | low |
| TCGA-FD-A3SJ | 2.836311 | 2.76934 | 2.497418 | 2.464376 | 2.441944 | 3.176865 | 2.788397 | low |
| TCGA-G2-A3VY | 2.992481 | 2.76934 | 2.497418 | 2.414332 | 2.443023 | 3.176865 | 2.624052 | low |
| TCGA-GC-A3WC | 2.816592 | 2.799831 | 7.964043 | 4.373659 | 2.480542 | 3.201567 | 4.09485 | high |
| TCGA-CU-A3QU | 2.80902 | 2.76934 | 2.497418 | 2.53596 | 2.434226 | 3.176865 | 2.615272 | low |
| TCGA-FD-A3SP | 2.645397 | 5.786361 | 3.40221 | 2.382946 | 2.559309 | 3.359324 | 3.016982 | high |
| TCGA-GC-A3RB | 2.792513 | 2.874995 | 2.497418 | 2.792874 | 2.459116 | 3.211866 | 3.377484 | low |
| TCGA-GU-AATQ | 2.767263 | 2.76934 | 2.843605 | 3.643984 | 2.479492 | 3.176865 | 2.746114 | low |
| TCGA-GC-A6I3 | 2.862264 | 2.808044 | 2.497418 | 2.628853 | 2.449215 | 3.20818 | 2.615272 | low |
| TCGA-GC-A3RD | 3.287325 | 3.622289 | 2.523358 | 4.337158 | 3.411204 | 3.176865 | 5.064204 | high |
| TCGA-GD-A3OQ | 2.694688 | 3.450891 | 2.515921 | 5.252852 | 4.37128 | 3.21109 | 3.174479 | high |
| TCGA-CF-A47T | 2.679342 | 2.864937 | 2.497418 | 4.339218 | 2.446795 | 3.203125 | 3.586227 | low |
| TCGA-ZF-AA52 | 2.602857 | 2.880796 | 3.967544 | 2.580084 | 2.477784 | 3.268038 | 2.754385 | high |
| TCGA-XF-A8HG | 2.626168 | 2.76934 | 2.497418 | 2.382946 | 2.434226 | 3.176865 | 2.615272 | low |
| TCGA-FD-A3B7 | 2.698134 | 2.76934 | 3.332328 | 3.603821 | 5.767582 | 3.195909 | 2.837826 | high |
| TCGA-DK-A3IL | 2.635608 | 2.76934 | 2.497418 | 4.549244 | 2.445719 | 3.176865 | 5.30817 | high |
| TCGA-E7-A6ME | 2.607663 | 2.805311 | 2.497418 | 2.382946 | 2.488784 | 3.206043 | 2.615272 | low |
| TCGA-GD-A2C5 | 2.65812 | 2.794637 | 2.519645 | 2.382946 | 2.519604 | 3.176865 | 2.788397 | low |
| TCGA-DK-AA6X | 2.605121 | 2.76934 | 2.520972 | 2.524966 | 2.731544 | 3.304062 | 2.926197 | low |
| TCGA-UY-A9PD | 2.753958 | 2.76934 | 2.497418 | 3.157679 | 2.434226 | 3.176865 | 2.834819 | low |
| TCGA-DK-A3IM | 6.116848 | 5.488279 | 2.613144 | 6.489902 | 3.810904 | 3.193954 | 8.448569 | high |
| TCGA-BT-A42F | 2.746555 | 2.76934 | 2.543601 | 4.542413 | 3.761649 | 3.440194 | 3.038705 | high |
| TCGA-FD-A3SL | 2.911307 | 2.76934 | 2.510183 | 2.382946 | 2.445449 | 3.200398 | 2.821199 | low |
| TCGA-BT-A3PH | 2.649103 | 2.790961 | 2.516321 | 4.778513 | 3.435905 | 3.194345 | 2.631765 | low |
| TCGA-FD-A3SQ | 2.597324 | 2.76934 | 2.581804 | 2.382946 | 2.518711 | 3.199618 | 3.218222 | low |
| TCGA-ZF-A9RF | 2.601724 | 2.76934 | 2.523888 | 2.382946 | 5.195005 | 4.4306 | 2.694486 | high |
| TCGA-DK-A1A3 | 2.653482 | 2.76934 | 2.497418 | 2.743255 | 2.556582 | 3.203709 | 2.804793 | low |
| TCGA-BT-A2LA | 2.62324 | 2.76934 | 2.549719 | 2.726272 | 3.168445 | 3.257272 | 2.615272 | low |
| TCGA-PQ-A6FN | 3.381412 | 3.000289 | 2.543209 | 7.173865 | 5.41746 | 3.261056 | 6.108323 | high |
| TCGA-GD-A3OP | 4.116155 | 2.810772 | 2.506567 | 2.382946 | 2.450154 | 3.210509 | 2.631134 | low |
| TCGA-FD-A5BT | 2.607663 | 2.941968 | 2.497418 | 2.432529 | 4.19012 | 3.346151 | 2.7846 | high |
| TCGA-BL-A3JM | 2.853863 | 13.015299 | 2.506165 | 2.382946 | 2.441944 | 3.317518 | 2.645556 | high |
| TCGA-E7-A85H | 4.70669 | 2.76934 | 2.497418 | 2.382946 | 2.453907 | 3.238814 | 2.625193 | low |
| TCGA-CU-A72E | 2.747962 | 2.76934 | 2.72954 | 6.097166 | 2.477915 | 3.176865 | 5.8333 | high |
| TCGA-FD-A3SN | 2.670324 | 2.908629 | 2.510049 | 2.534843 | 3.90523 | 3.246065 | 2.669644 | high |
| TCGA-GU-A762 | 2.597324 | 2.76934 | 2.510316 | 2.382946 | 6.358217 | 3.176865 | 2.64867 | high |
| TCGA-GV-A3QH | 2.618487 | 2.831978 | 2.497418 | 3.65037 | 2.434226 | 3.176865 | 2.623418 | low |
| TCGA-FD-A6TF | 2.609073 | 3.666933 | 2.497418 | 4.728901 | 5.79692 | 3.285105 | 2.914331 | high |
| TCGA-XF-A9SJ | 2.606533 | 3.521119 | 2.497418 | 2.469214 | 3.374749 | 3.25329 | 2.787504 | high |
| TCGA-ZF-A9RL | 2.726061 | 2.76934 | 2.497418 | 2.382946 | 2.448006 | 3.176865 | 3.636387 | low |
| TCGA-E7-A678 | 2.615122 | 2.76934 | 2.515522 | 2.382946 | 2.434226 | 3.176865 | 2.615272 | low |
| TCGA-GD-A76B | 2.657847 | 2.983035 | 2.508443 | 2.824545 | 2.463109 | 3.217478 | 2.62494 | low |
| TCGA-GV-A6ZA | 2.597324 | 2.861716 | 2.524814 | 2.508292 | 2.458316 | 3.227502 | 2.662773 | low |
| TCGA-FD-A5C1 | 2.60611 | 2.76934 | 2.721444 | 7.463787 | 5.851999 | 3.201762 | 5.997054 | high |
| TCGA-BT-A20X | 5.832421 | 5.952059 | 2.675616 | 7.437525 | 5.217042 | 3.312736 | 4.193582 | high |
| TCGA-K4-A3WV | 2.615122 | 2.973927 | 2.497418 | 8.961122 | 3.592591 | 3.201957 | 5.624463 | high |
| TCGA-FD-A5C0 | 2.733313 | 2.76934 | 2.510183 | 3.373958 | 2.542363 | 3.269544 | 4.253905 | high |
| TCGA-DK-A3IU | 4.147398 | 3.289713 | 2.517252 | 4.242283 | 4.368278 | 3.319354 | 7.721289 | high |
| TCGA-UY-A78K | 2.931899 | 2.76934 | 2.520972 | 3.441925 | 2.444641 | 3.176865 | 2.676113 | low |
| TCGA-DK-A1AE | 2.828481 | 2.76934 | 9.063965 | 5.772256 | 2.473174 | 3.176865 | 2.697962 | high |
| TCGA-LT-A8JT | 2.60865 | 2.76934 | 2.508979 | 2.454286 | 2.434226 | 3.176865 | 2.674529 | low |
| TCGA-BT-A20O | 2.602432 | 2.76934 | 8.367909 | 2.478016 | 2.447871 | 3.31568 | 2.68158 | high |
| TCGA-K4-A4AB | 2.828481 | 2.76934 | 2.652707 | 2.849792 | 5.679906 | 3.176865 | 2.673798 | high |
| TCGA-FD-A43U | 2.64526 | 2.76934 | 2.524285 | 2.720012 | 2.469342 | 3.75762 | 3.093249 | high |
| TCGA-FD-A6TE | 2.637129 | 2.76934 | 2.497418 | 2.603064 | 2.605805 | 3.211672 | 2.671967 | low |
| TCGA-XF-A9SV | 2.664777 | 2.76934 | 2.574428 | 2.540087 | 2.587131 | 3.176865 | 2.615272 | low |
| TCGA-GD-A6C6 | 2.807794 | 2.76934 | 2.497418 | 2.382946 | 2.460981 | 3.176865 | 2.62861 | low |
| TCGA-KQ-A41N | 2.710658 | 2.76934 | 2.497418 | 2.427913 | 2.738832 | 3.562999 | 2.640183 | low |
| TCGA-4Z-AA7W | 2.665319 | 2.76934 | 2.576976 | 3.508174 | 2.694503 | 3.20682 | 2.724846 | low |
| TCGA-CF-A9FH | 2.597324 | 2.76934 | 2.497418 | 2.382946 | 2.434226 | 3.207403 | 2.615272 | low |
| TCGA-KQ-A41Q | 2.611186 | 2.76934 | 2.707419 | 2.551172 | 2.434226 | 3.176865 | 2.710823 | low |
| TCGA-E7-A3X6 | 2.728525 | 2.76934 | 2.638189 | 2.598501 | 2.587616 | 3.176865 | 4.37759 | high |
| TCGA-XF-A9SL | 2.602007 | 2.832874 | 2.552832 | 2.382946 | 2.552854 | 3.25367 | 2.710468 | low |
| TCGA-4Z-AA81 | 2.602574 | 6.988134 | 2.513255 | 4.158862 | 5.17152 | 3.176865 | 2.656359 | high |
| TCGA-DK-A1AB | 3.148113 | 2.914306 | 2.840154 | 3.580617 | 3.527233 | 3.207209 | 7.104349 | high |
| TCGA-XF-A9T0 | 2.941415 | 2.805311 | 3.060658 | 3.035931 | 2.461779 | 3.206043 | 3.329276 | high |
| TCGA-GC-A3RC | 8.382833 | 2.76934 | 2.5347 | 8.803492 | 3.909543 | 3.176865 | 9.833192 | high |
| TCGA-XF-AAN8 | 2.597324 | 2.816214 | 2.497418 | 2.382946 | 2.4523 | 3.28865 | 3.10592 | low |
| TCGA-XF-A8HF | 4.087351 | 2.76934 | 2.497418 | 3.215096 | 2.470003 | 3.202151 | 4.706586 | high |
| TCGA-E5-A2PC | 2.676924 | 2.76934 | 2.531544 | 2.382946 | 2.456714 | 3.176865 | 2.645057 | low |
| TCGA-ZF-A9R2 | 2.788667 | 2.76934 | 2.544775 | 2.614871 | 2.489436 | 3.176865 | 2.77348 | low |
| TCGA-E5-A4U1 | 2.706057 | 2.76934 | 2.497418 | 2.382946 | 2.434226 | 3.205654 | 5.730109 | high |
| TCGA-BT-A0S7 | 3.336331 | 2.76934 | 2.497418 | 8.144131 | 2.570408 | 3.251012 | 6.841331 | high |
| TCGA-ZF-A9RM | 3.671989 | 2.827041 | 2.497418 | 2.422914 | 2.445449 | 3.176865 | 2.637425 | low |
| TCGA-UY-A9PB | 2.608227 | 6.088653 | 2.683873 | 2.532157 | 3.602792 | 3.296093 | 2.615272 | high |
| TCGA-XF-A9SY | 2.657438 | 2.882243 | 2.673248 | 2.6717 | 3.634291 | 3.269168 | 2.630125 | high |
| TCGA-E7-A7DU | 2.670999 | 2.76934 | 2.497418 | 2.454167 | 2.434226 | 3.219024 | 2.615272 | low |
| TCGA-XF-AAMJ | 2.688036 | 2.854372 | 2.535094 | 2.382946 | 2.51539 | 3.345788 | 2.648172 | low |

# Appendix 9

## **GO and KEGG enrichment analysis**

**Table 9a. GO enrichment analysis.**

| ONTOLOGY | ID | Description | BgRatio | pvalue | qvalue |
| --- | --- | --- | --- | --- | --- |
| BP | GO:0072527 | pyrimidine-containing compound metabolic process | 85/18862 | 2.97E-50 | 1.42E-47 |
| BP | GO:0006220 | pyrimidine nucleotide metabolic process | 50/18862 | 6.54E-46 | 1.57E-43 |
| BP | GO:0034404 | nucleobase-containing small molecule biosynthetic process | 115/18862 | 1.33E-41 | 2.12E-39 |
| BP | GO:0072528 | pyrimidine-containing compound biosynthetic process | 40/18862 | 1.07E-38 | 1.29E-36 |
| BP | GO:0006221 | pyrimidine nucleotide biosynthetic process | 30/18862 | 3.26E-34 | 3.12E-32 |
| BP | GO:0006213 | pyrimidine nucleoside metabolic process | 36/18862 | 2.30E-32 | 1.83E-30 |
| BP | GO:0009123 | nucleoside monophosphate metabolic process | 75/18862 | 5.66E-32 | 3.88E-30 |
| BP | GO:0009116 | nucleoside metabolic process | 104/18862 | 9.93E-31 | 5.94E-29 |
| BP | GO:0009163 | nucleoside biosynthetic process | 38/18862 | 1.85E-29 | 9.84E-28 |
| BP | GO:1901657 | glycosyl compound metabolic process | 129/18862 | 1.33E-28 | 5.96E-27 |
| BP | GO:1901659 | glycosyl compound biosynthetic process | 42/18862 | 1.37E-28 | 5.96E-27 |
| BP | GO:0009147 | pyrimidine nucleoside triphosphate metabolic process | 25/18862 | 3.60E-28 | 1.44E-26 |
| BP | GO:0009129 | pyrimidine nucleoside monophosphate metabolic process | 19/18862 | 6.65E-28 | 2.45E-26 |
| BP | GO:0009218 | pyrimidine ribonucleotide metabolic process | 29/18862 | 6.18E-27 | 2.11E-25 |
| BP | GO:0009148 | pyrimidine nucleoside triphosphate biosynthetic process | 19/18862 | 3.62E-25 | 1.16E-23 |
| BP | GO:0009262 | deoxyribonucleotide metabolic process | 40/18862 | 1.79E-24 | 5.35E-23 |
| BP | GO:0009220 | pyrimidine ribonucleotide biosynthetic process | 22/18862 | 4.61E-24 | 1.30E-22 |
| BP | GO:0046134 | pyrimidine nucleoside biosynthetic process | 16/18862 | 9.17E-24 | 2.44E-22 |
| BP | GO:1901293 | nucleoside phosphate biosynthetic process | 267/18862 | 3.21E-23 | 8.10E-22 |
| BP | GO:0032201 | telomere maintenance via semi-conservative replication | 27/18862 | 1.22E-22 | 2.92E-21 |
| BP | GO:0009130 | pyrimidine nucleoside monophosphate biosynthetic process | 14/18862 | 6.02E-22 | 1.37E-20 |
| BP | GO:0032481 | positive regulation of type I interferon production | 77/18862 | 8.02E-22 | 1.73E-20 |
| BP | GO:0009124 | nucleoside monophosphate biosynthetic process | 43/18862 | 8.32E-22 | 1.73E-20 |
| BP | GO:0009219 | pyrimidine deoxyribonucleotide metabolic process | 23/18862 | 2.78E-21 | 5.54E-20 |
| BP | GO:0009141 | nucleoside triphosphate metabolic process | 109/18862 | 4.01E-21 | 7.68E-20 |
| BP | GO:0009394 | 2'-deoxyribonucleotide metabolic process | 36/18862 | 8.54E-21 | 1.57E-19 |
| BP | GO:0019692 | deoxyribose phosphate metabolic process | 38/18862 | 1.83E-20 | 3.25E-19 |
| BP | GO:0009165 | nucleotide biosynthetic process | 264/18862 | 2.19E-20 | 3.74E-19 |
| BP | GO:0015949 | nucleobase-containing small molecule interconversion | 27/18862 | 2.64E-20 | 4.37E-19 |
| BP | GO:0032479 | regulation of type I interferon production | 128/18862 | 5.92E-20 | 9.45E-19 |
| BP | GO:0032606 | type I interferon production | 129/18862 | 6.74E-20 | 1.04E-18 |
| BP | GO:1901292 | nucleoside phosphate catabolic process | 83/18862 | 1.78E-19 | 2.67E-18 |
| BP | GO:0009173 | pyrimidine ribonucleoside monophosphate metabolic process | 14/18862 | 3.38E-19 | 4.76E-18 |
| BP | GO:0046049 | UMP metabolic process | 14/18862 | 3.38E-19 | 4.76E-18 |
| BP | GO:0009209 | pyrimidine ribonucleoside triphosphate biosynthetic process | 15/18862 | 8.42E-19 | 1.15E-17 |
| BP | GO:0006283 | transcription-coupled nucleotide-excision repair | 73/18862 | 1.79E-18 | 2.38E-17 |
| BP | GO:0009162 | deoxyribonucleoside monophosphate metabolic process | 17/18862 | 4.06E-18 | 5.26E-17 |
| BP | GO:0033260 | nuclear DNA replication | 59/18862 | 7.10E-18 | 8.95E-17 |
| BP | GO:0009208 | pyrimidine ribonucleoside triphosphate metabolic process | 18/18862 | 8.10E-18 | 9.94E-17 |
| BP | GO:0006289 | nucleotide-excision repair | 108/18862 | 8.73E-18 | 1.05E-16 |
| BP | GO:0044786 | cell cycle DNA replication | 64/18862 | 2.05E-17 | 2.40E-16 |
| BP | GO:0034656 | nucleobase-containing small molecule catabolic process | 50/18862 | 7.04E-17 | 8.03E-16 |
| BP | GO:0006241 | CTP biosynthetic process | 14/18862 | 1.40E-16 | 1.56E-15 |
| BP | GO:0009263 | deoxyribonucleotide biosynthetic process | 15/18862 | 2.99E-16 | 3.25E-15 |
| BP | GO:0072529 | pyrimidine-containing compound catabolic process | 40/18862 | 4.69E-16 | 4.99E-15 |
| BP | GO:0050434 | positive regulation of viral transcription | 26/18862 | 5.07E-16 | 5.28E-15 |
| BP | GO:0006206 | pyrimidine nucleobase metabolic process | 16/18862 | 5.96E-16 | 5.95E-15 |
| BP | GO:0046036 | CTP metabolic process | 16/18862 | 5.96E-16 | 5.95E-15 |
| BP | GO:0009142 | nucleoside triphosphate biosynthetic process | 84/18862 | 6.63E-16 | 6.48E-15 |
| BP | GO:0006354 | DNA-templated transcription, elongation | 119/18862 | 1.40E-15 | 1.34E-14 |
| BP | GO:0046434 | organophosphate catabolic process | 154/18862 | 1.43E-15 | 1.34E-14 |
| BP | GO:0006222 | UMP biosynthetic process | 10/18862 | 1.54E-15 | 1.37E-14 |
| BP | GO:0009174 | pyrimidine ribonucleoside monophosphate biosynthetic process | 10/18862 | 1.54E-15 | 1.37E-14 |
| BP | GO:0009176 | pyrimidine deoxyribonucleoside monophosphate metabolic process | 10/18862 | 1.54E-15 | 1.37E-14 |
| BP | GO:0006362 | transcription elongation from RNA polymerase I promoter | 30/18862 | 2.29E-15 | 2.00E-14 |
| BP | GO:0006363 | termination of RNA polymerase I transcription | 31/18862 | 3.22E-15 | 2.75E-14 |
| BP | GO:0008655 | pyrimidine-containing compound salvage | 11/18862 | 4.23E-15 | 3.49E-14 |
| BP | GO:0043097 | pyrimidine nucleoside salvage | 11/18862 | 4.23E-15 | 3.49E-14 |
| BP | GO:0006370 | 7-methylguanosine mRNA capping | 33/18862 | 6.12E-15 | 4.97E-14 |
| BP | GO:0009452 | 7-methylguanosine RNA capping | 34/18862 | 8.30E-15 | 6.52E-14 |
| BP | GO:0036260 | RNA capping | 34/18862 | 8.30E-15 | 6.52E-14 |
| BP | GO:0006260 | DNA replication | 280/18862 | 1.77E-14 | 1.36E-13 |
| BP | GO:0009161 | ribonucleoside monophosphate metabolic process | 56/18862 | 1.87E-14 | 1.42E-13 |
| BP | GO:0046135 | pyrimidine nucleoside catabolic process | 23/18862 | 2.22E-14 | 1.66E-13 |
| BP | GO:0006361 | transcription initiation from RNA polymerase I promoter | 38/18862 | 2.55E-14 | 1.88E-13 |
| BP | GO:0046782 | regulation of viral transcription | 42/18862 | 6.88E-14 | 4.99E-13 |
| BP | GO:0043174 | nucleoside salvage | 16/18862 | 1.44E-13 | 1.03E-12 |
| BP | GO:0006383 | transcription by RNA polymerase III | 47/18862 | 2.07E-13 | 1.46E-12 |
| BP | GO:0009223 | pyrimidine deoxyribonucleotide catabolic process | 18/18862 | 3.99E-13 | 2.77E-12 |
| BP | GO:0006353 | DNA-templated transcription, termination | 75/18862 | 4.10E-13 | 2.81E-12 |
| BP | GO:0098781 | ncRNA transcription | 109/18862 | 6.00E-13 | 4.05E-12 |
| BP | GO:0009112 | nucleobase metabolic process | 34/18862 | 7.94E-13 | 5.21E-12 |
| BP | GO:0009164 | nucleoside catabolic process | 34/18862 | 7.94E-13 | 5.21E-12 |
| BP | GO:0006244 | pyrimidine nucleotide catabolic process | 20/18862 | 9.66E-13 | 6.25E-12 |
| BP | GO:0006352 | DNA-templated transcription, initiation | 249/18862 | 1.12E-12 | 7.18E-12 |
| BP | GO:0006261 | DNA-dependent DNA replication | 157/18862 | 1.42E-12 | 8.94E-12 |
| BP | GO:0045815 | positive regulation of gene expression, epigenetic | 58/18862 | 1.56E-12 | 9.70E-12 |
| BP | GO:0000723 | telomere maintenance | 161/18862 | 1.91E-12 | 1.18E-11 |
| BP | GO:0009265 | 2'-deoxyribonucleotide biosynthetic process | 12/18862 | 3.17E-12 | 1.90E-11 |
| BP | GO:0046385 | deoxyribose phosphate biosynthetic process | 12/18862 | 3.17E-12 | 1.90E-11 |
| BP | GO:0006270 | DNA replication initiation | 40/18862 | 3.30E-12 | 1.95E-11 |
| BP | GO:0032200 | telomere organization | 174/18862 | 4.82E-12 | 2.81E-11 |
| BP | GO:0009125 | nucleoside monophosphate catabolic process | 13/18862 | 5.87E-12 | 3.39E-11 |
| BP | GO:0006360 | transcription by RNA polymerase I | 68/18862 | 6.99E-12 | 3.99E-11 |
| BP | GO:1901658 | glycosyl compound catabolic process | 44/18862 | 7.50E-12 | 4.23E-11 |
| BP | GO:0009264 | deoxyribonucleotide catabolic process | 26/18862 | 8.04E-12 | 4.48E-11 |
| BP | GO:0035019 | somatic stem cell population maintenance | 71/18862 | 1.05E-11 | 5.71E-11 |
| BP | GO:0009259 | ribonucleotide metabolic process | 425/18862 | 1.05E-11 | 5.71E-11 |
| BP | GO:0046386 | deoxyribose phosphate catabolic process | 27/18862 | 1.08E-11 | 5.82E-11 |
| BP | GO:0009260 | ribonucleotide biosynthetic process | 188/18862 | 1.20E-11 | 6.40E-11 |
| BP | GO:0009166 | nucleotide catabolic process | 73/18862 | 1.36E-11 | 7.06E-11 |
| BP | GO:0009201 | ribonucleoside triphosphate biosynthetic process | 73/18862 | 1.36E-11 | 7.06E-11 |
| BP | GO:0019693 | ribose phosphate metabolic process | 435/18862 | 1.49E-11 | 7.65E-11 |
| BP | GO:0042795 | snRNA transcription by RNA polymerase II | 74/18862 | 1.54E-11 | 7.84E-11 |
| BP | GO:0009301 | snRNA transcription | 75/18862 | 1.74E-11 | 8.78E-11 |
| BP | GO:0046390 | ribose phosphate biosynthetic process | 195/18862 | 1.85E-11 | 9.21E-11 |
| BP | GO:0009156 | ribonucleoside monophosphate biosynthetic process | 34/18862 | 6.41E-11 | 3.13E-10 |
| BP | GO:0043094 | cellular metabolic compound salvage | 34/18862 | 6.41E-11 | 3.13E-10 |
| BP | GO:0009199 | ribonucleoside triphosphate metabolic process | 87/18862 | 6.85E-11 | 3.31E-10 |
| BP | GO:0006368 | transcription elongation from RNA polymerase II promoter | 90/18862 | 9.34E-11 | 4.47E-10 |
| BP | GO:0048524 | positive regulation of viral process | 91/18862 | 1.03E-10 | 4.90E-10 |
| BP | GO:0001819 | positive regulation of cytokine production | 437/18862 | 1.83E-10 | 8.59E-10 |
| BP | GO:0006228 | UTP biosynthetic process | 10/18862 | 2.30E-10 | 1.07E-09 |
| BP | GO:0071897 | DNA biosynthetic process | 194/18862 | 3.28E-10 | 1.51E-09 |
| BP | GO:0046051 | UTP metabolic process | 12/18862 | 7.20E-10 | 3.28E-09 |
| BP | GO:0008543 | fibroblast growth factor receptor signaling pathway | 113/18862 | 7.33E-10 | 3.31E-09 |
| BP | GO:0060964 | regulation of gene silencing by miRNA | 121/18862 | 1.35E-09 | 6.05E-09 |
| BP | GO:0060147 | regulation of posttranscriptional gene silencing | 124/18862 | 1.68E-09 | 7.46E-09 |
| BP | GO:0000731 | DNA synthesis involved in DNA repair | 53/18862 | 1.73E-09 | 7.59E-09 |
| BP | GO:0060966 | regulation of gene silencing by RNA | 125/18862 | 1.81E-09 | 7.87E-09 |
| BP | GO:0009200 | deoxyribonucleoside triphosphate metabolic process | 16/18862 | 3.92E-09 | 1.69E-08 |
| BP | GO:0044344 | cellular response to fibroblast growth factor stimulus | 142/18862 | 5.59E-09 | 2.39E-08 |
| BP | GO:0060968 | regulation of gene silencing | 144/18862 | 6.32E-09 | 2.68E-08 |
| BP | GO:1901136 | carbohydrate derivative catabolic process | 198/18862 | 6.77E-09 | 2.84E-08 |
| BP | GO:0019827 | stem cell population maintenance | 146/18862 | 7.14E-09 | 2.97E-08 |
| BP | GO:0071774 | response to fibroblast growth factor | 148/18862 | 8.04E-09 | 3.29E-08 |
| BP | GO:0098727 | maintenance of cell number | 148/18862 | 8.04E-09 | 3.29E-08 |
| BP | GO:0006271 | DNA strand elongation involved in DNA replication | 19/18862 | 1.03E-08 | 4.20E-08 |
| BP | GO:0009132 | nucleoside diphosphate metabolic process | 156/18862 | 1.28E-08 | 5.13E-08 |
| BP | GO:0009119 | ribonucleoside metabolic process | 72/18862 | 1.55E-08 | 6.20E-08 |
| BP | GO:0042451 | purine nucleoside biosynthetic process | 22/18862 | 2.32E-08 | 9.03E-08 |
| BP | GO:0042455 | ribonucleoside biosynthetic process | 22/18862 | 2.32E-08 | 9.03E-08 |
| BP | GO:0046129 | purine ribonucleoside biosynthetic process | 22/18862 | 2.32E-08 | 9.03E-08 |
| BP | GO:0006297 | nucleotide-excision repair, DNA gap filling | 23/18862 | 2.95E-08 | 1.14E-07 |
| BP | GO:0019083 | viral transcription | 180/18862 | 4.42E-08 | 1.69E-07 |
| BP | GO:0022616 | DNA strand elongation | 26/18862 | 5.72E-08 | 2.17E-07 |
| BP | GO:0050792 | regulation of viral process | 186/18862 | 5.86E-08 | 2.21E-07 |
| BP | GO:0006367 | transcription initiation from RNA polymerase II promoter | 187/18862 | 6.14E-08 | 2.30E-07 |
| BP | GO:0006183 | GTP biosynthetic process | 11/18862 | 7.86E-08 | 2.92E-07 |
| BP | GO:0043903 | regulation of biological process involved in symbiotic interaction | 197/18862 | 9.59E-08 | 3.53E-07 |
| BP | GO:0019080 | viral gene expression | 198/18862 | 1.00E-07 | 3.66E-07 |
| BP | GO:0046128 | purine ribonucleoside metabolic process | 58/18862 | 1.20E-07 | 4.35E-07 |
| BP | GO:0040029 | regulation of gene expression, epigenetic | 205/18862 | 1.35E-07 | 4.85E-07 |
| BP | GO:0000082 | G1/S transition of mitotic cell cycle | 275/18862 | 1.51E-07 | 5.39E-07 |
| BP | GO:0042278 | purine nucleoside metabolic process | 61/18862 | 1.63E-07 | 5.78E-07 |
| BP | GO:1901070 | guanosine-containing compound biosynthetic process | 13/18862 | 1.69E-07 | 5.96E-07 |
| BP | GO:0032728 | positive regulation of interferon-beta production | 33/18862 | 2.02E-07 | 7.06E-07 |
| BP | GO:0009143 | nucleoside triphosphate catabolic process | 14/18862 | 2.36E-07 | 8.14E-07 |
| BP | GO:0043173 | nucleotide salvage | 14/18862 | 2.36E-07 | 8.14E-07 |
| BP | GO:0044843 | cell cycle G1/S phase transition | 298/18862 | 3.17E-07 | 1.08E-06 |
| BP | GO:0060249 | anatomical structure homeostasis | 466/18862 | 3.32E-07 | 1.13E-06 |
| BP | GO:0090305 | nucleic acid phosphodiester bond hydrolysis | 305/18862 | 3.92E-07 | 1.32E-06 |
| BP | GO:0019985 | translesion synthesis | 42/18862 | 7.04E-07 | 2.36E-06 |
| BP | GO:0046112 | nucleobase biosynthetic process | 18/18862 | 7.13E-07 | 2.37E-06 |
| BP | GO:0051607 | defense response to virus | 260/18862 | 9.96E-07 | 3.27E-06 |
| BP | GO:0140546 | defense response to symbiont | 260/18862 | 9.96E-07 | 3.27E-06 |
| BP | GO:0044282 | small molecule catabolic process | 431/18862 | 1.18E-06 | 3.83E-06 |
| BP | GO:0032648 | regulation of interferon-beta production | 50/18862 | 1.71E-06 | 5.53E-06 |
| BP | GO:0006301 | postreplication repair | 52/18862 | 2.08E-06 | 6.65E-06 |
| BP | GO:0032608 | interferon-beta production | 52/18862 | 2.08E-06 | 6.65E-06 |
| BP | GO:0046039 | GTP metabolic process | 24/18862 | 2.43E-06 | 7.72E-06 |
| BP | GO:0009133 | nucleoside diphosphate biosynthetic process | 10/18862 | 7.39E-06 | 2.33E-05 |
| BP | GO:0009134 | nucleoside diphosphate catabolic process | 12/18862 | 1.35E-05 | 4.22E-05 |
| BP | GO:0009615 | response to virus | 359/18862 | 1.37E-05 | 4.27E-05 |
| BP | GO:0006296 | nucleotide-excision repair, DNA incision, 5'-to lesion | 37/18862 | 1.45E-05 | 4.49E-05 |
| BP | GO:0072521 | purine-containing compound metabolic process | 460/18862 | 1.53E-05 | 4.68E-05 |
| BP | GO:0006165 | nucleoside diphosphate phosphorylation | 132/18862 | 1.53E-05 | 4.68E-05 |
| BP | GO:0046939 | nucleotide phosphorylation | 133/18862 | 1.60E-05 | 4.85E-05 |
| BP | GO:0006298 | mismatch repair | 38/18862 | 1.62E-05 | 4.88E-05 |
| BP | GO:0033683 | nucleotide-excision repair, DNA incision | 39/18862 | 1.80E-05 | 5.35E-05 |
| BP | GO:0042769 | DNA damage response, detection of DNA damage | 39/18862 | 1.80E-05 | 5.35E-05 |
| BP | GO:0009261 | ribonucleotide catabolic process | 40/18862 | 1.99E-05 | 5.85E-05 |
| BP | GO:1901068 | guanosine-containing compound metabolic process | 40/18862 | 1.99E-05 | 5.85E-05 |
| BP | GO:0000377 | RNA splicing, via transesterification reactions with bulged adenosine as nucleophile | 383/18862 | 2.29E-05 | 6.65E-05 |
| BP | GO:0000398 | mRNA splicing, via spliceosome | 383/18862 | 2.29E-05 | 6.65E-05 |
| BP | GO:0000375 | RNA splicing, via transesterification reactions | 386/18862 | 2.44E-05 | 7.03E-05 |
| BP | GO:0009126 | purine nucleoside monophosphate metabolic process | 43/18862 | 2.67E-05 | 7.65E-05 |
| BP | GO:0007595 | lactation | 46/18862 | 3.49E-05 | 9.96E-05 |
| BP | GO:0072523 | purine-containing compound catabolic process | 51/18862 | 5.27E-05 | 0.000149302 |
| BP | GO:0006163 | purine nucleotide metabolic process | 441/18862 | 6.85E-05 | 0.000193049 |
| BP | GO:0006359 | regulation of transcription by RNA polymerase III | 23/18862 | 0.000105055 | 0.000294243 |
| BP | GO:0008380 | RNA splicing | 481/18862 | 0.00013251 | 0.000368986 |
| BP | GO:0009206 | purine ribonucleoside triphosphate biosynthetic process | 67/18862 | 0.000153901 | 0.000426073 |
| BP | GO:0009145 | purine nucleoside triphosphate biosynthetic process | 68/18862 | 0.000163023 | 0.000448733 |
| BP | GO:0072522 | purine-containing compound biosynthetic process | 208/18862 | 0.000192469 | 0.000526756 |
| BP | GO:0009205 | purine ribonucleoside triphosphate metabolic process | 80/18862 | 0.000305257 | 0.000830693 |
| BP | GO:0000083 | regulation of transcription involved in G1/S transition of mitotic cell cycle | 35/18862 | 0.000374985 | 0.001014678 |
| BP | GO:0007589 | body fluid secretion | 86/18862 | 0.000402459 | 0.001076853 |
| BP | GO:0009144 | purine nucleoside triphosphate metabolic process | 86/18862 | 0.000402459 | 0.001076853 |
| BP | GO:0006195 | purine nucleotide catabolic process | 46/18862 | 0.000842493 | 0.002241721 |
| BP | GO:0006164 | purine nucleotide biosynthetic process | 197/18862 | 0.001197243 | 0.003168047 |
| BP | GO:0046940 | nucleoside monophosphate phosphorylation | 13/18862 | 0.00121429 | 0.003195501 |
| BP | GO:0009150 | purine ribonucleotide metabolic process | 408/18862 | 0.001275529 | 0.003338313 |
| BP | GO:0048732 | gland development | 413/18862 | 0.001367927 | 0.003560679 |
| BP | GO:0009151 | purine deoxyribonucleotide metabolic process | 14/18862 | 0.001412978 | 0.003658066 |
| BP | GO:0030879 | mammary gland development | 132/18862 | 0.001991974 | 0.005129306 |
| BP | GO:0031000 | response to caffeine | 17/18862 | 0.002095238 | 0.005346151 |
| BP | GO:0035690 | cellular response to drug | 63/18862 | 0.002098511 | 0.005346151 |
| BP | GO:0001889 | liver development | 138/18862 | 0.002341537 | 0.00593372 |
| BP | GO:0061008 | hepaticobiliary system development | 140/18862 | 0.002466872 | 0.006218432 |
| BP | GO:0014074 | response to purine-containing compound | 144/18862 | 0.002731258 | 0.006848842 |
| BP | GO:0031100 | animal organ regeneration | 73/18862 | 0.003193482 | 0.007966197 |
| BP | GO:0016311 | dephosphorylation | 491/18862 | 0.003610076 | 0.008958739 |
| BP | GO:0000288 | nuclear-transcribed mRNA catabolic process, deadenylation-dependent decay | 77/18862 | 0.003712981 | 0.009166611 |
| BP | GO:0006541 | glutamine metabolic process | 23/18862 | 0.00383729 | 0.009424924 |
| BP | GO:0009152 | purine ribonucleotide biosynthetic process | 175/18862 | 0.005466673 | 0.013358413 |
| BP | GO:0007565 | female pregnancy | 177/18862 | 0.00568879 | 0.013830615 |
| BP | GO:0045948 | positive regulation of translational initiation | 30/18862 | 0.00647868 | 0.015671448 |
| BP | GO:0097421 | liver regeneration | 31/18862 | 0.006907518 | 0.016624812 |
| BP | GO:0006356 | regulation of transcription by RNA polymerase I | 33/18862 | 0.007802748 | 0.018592565 |
| BP | GO:0009303 | rRNA transcription | 33/18862 | 0.007802748 | 0.018592565 |
| BP | GO:0000291 | nuclear-transcribed mRNA catabolic process, exonucleolytic | 35/18862 | 0.008747351 | 0.020638032 |
| BP | GO:0009154 | purine ribonucleotide catabolic process | 35/18862 | 0.008747351 | 0.020638032 |
| BP | GO:0044706 | multi-multicellular organism process | 204/18862 | 0.009297745 | 0.021829072 |
| BP | GO:0071398 | cellular response to fatty acid | 38/18862 | 0.010255015 | 0.023959085 |
| BP | GO:0006284 | base-excision repair | 39/18862 | 0.010781363 | 0.024945436 |
| BP | GO:0060416 | response to growth hormone | 39/18862 | 0.010781363 | 0.024945436 |
| BP | GO:0009167 | purine ribonucleoside monophosphate metabolic process | 40/18862 | 0.011319438 | 0.026064496 |
| BP | GO:0033574 | response to testosterone | 41/18862 | 0.011869141 | 0.027199491 |
| BP | GO:0014075 | response to amine | 42/18862 | 0.01243037 | 0.028349967 |
| BP | GO:0045089 | positive regulation of innate immune response | 223/18862 | 0.012572978 | 0.028539311 |
| BP | GO:0045454 | cell redox homeostasis | 43/18862 | 0.013003028 | 0.029376254 |
| BP | GO:0042493 | response to drug | 359/18862 | 0.014915829 | 0.033539424 |
| BP | GO:0046683 | response to organophosphorus | 130/18862 | 0.015604604 | 0.034924224 |
| BP | GO:0002762 | negative regulation of myeloid leukocyte differentiation | 48/18862 | 0.016034302 | 0.035719009 |
| BP | GO:0045005 | DNA-dependent DNA replication maintenance of fidelity | 49/18862 | 0.016673476 | 0.036970914 |
| BP | GO:0061014 | positive regulation of mRNA catabolic process | 52/18862 | 0.018655117 | 0.041174283 |
| BP | GO:0002833 | positive regulation of response to biotic stimulus | 258/18862 | 0.02036408 | 0.044740011 |
| CC | GO:0061695 | transferase complex, transferring phosphorus-containing groups | 253/19520 | 4.20E-50 | 2.87E-48 |
| CC | GO:0055029 | nuclear DNA-directed RNA polymerase complex | 103/19520 | 7.99E-48 | 2.52E-46 |
| CC | GO:0000428 | DNA-directed RNA polymerase complex | 104/19520 | 1.11E-47 | 2.52E-46 |
| CC | GO:0030880 | RNA polymerase complex | 108/19520 | 3.90E-47 | 6.68E-46 |
| CC | GO:0005666 | RNA polymerase III complex | 18/19520 | 7.81E-38 | 1.07E-36 |
| CC | GO:0042575 | DNA polymerase complex | 20/19520 | 5.99E-25 | 6.83E-24 |
| CC | GO:0005665 | RNA polymerase II, core complex | 15/19520 | 1.97E-24 | 1.93E-23 |
| CC | GO:0005736 | RNA polymerase I complex | 13/19520 | 1.22E-22 | 1.05E-21 |
| CC | GO:0016591 | RNA polymerase II, holoenzyme | 79/19520 | 1.07E-14 | 7.57E-14 |
| CC | GO:0043601 | nuclear replisome | 22/19520 | 1.11E-14 | 7.57E-14 |
| CC | GO:0030894 | replisome | 24/19520 | 2.53E-14 | 1.57E-13 |
| CC | GO:0043596 | nuclear replication fork | 35/19520 | 7.82E-13 | 4.46E-12 |
| CC | GO:0005657 | replication fork | 65/19520 | 1.53E-10 | 8.04E-10 |
| CC | GO:0000228 | nuclear chromosome | 250/19520 | 2.25E-10 | 1.10E-09 |
| CC | GO:0032993 | protein-DNA complex | 208/19520 | 1.14E-07 | 5.22E-07 |
| CC | GO:0005671 | Ada2/Gcn5/Ada3 transcription activator complex | 15/19520 | 0.001520066 | 0.006500281 |
| MF | GO:0016779 | nucleotidyltransferase activity | 131/18337 | 3.70E-54 | 2.84E-52 |
| MF | GO:0003899 | DNA-directed 5'-3' RNA polymerase activity | 39/18337 | 4.69E-52 | 1.80E-50 |
| MF | GO:0034062 | 5'-3' RNA polymerase activity | 43/18337 | 1.87E-50 | 3.59E-49 |
| MF | GO:0097747 | RNA polymerase activity | 43/18337 | 1.87E-50 | 3.59E-49 |
| MF | GO:0140098 | catalytic activity, acting on RNA | 386/18337 | 2.40E-26 | 3.69E-25 |
| MF | GO:0019205 | nucleobase-containing compound kinase activity | 42/18337 | 1.13E-17 | 1.44E-16 |
| MF | GO:0003887 | DNA-directed DNA polymerase activity | 25/18337 | 6.09E-14 | 6.69E-13 |
| MF | GO:0034061 | DNA polymerase activity | 36/18337 | 1.64E-12 | 1.58E-11 |
| MF | GO:0008253 | 5'-nucleotidase activity | 13/18337 | 6.95E-12 | 5.93E-11 |
| MF | GO:0008252 | nucleotidase activity | 15/18337 | 2.01E-11 | 1.55E-10 |
| MF | GO:0004550 | nucleoside diphosphate kinase activity | 18/18337 | 7.40E-11 | 5.17E-10 |
| MF | GO:0016776 | phosphotransferase activity, phosphate group as acceptor | 39/18337 | 1.21E-08 | 7.77E-08 |
| MF | GO:0047429 | nucleoside-triphosphate diphosphatase activity | 11/18337 | 8.79E-08 | 5.20E-07 |
| MF | GO:0140097 | catalytic activity, acting on DNA | 204/18337 | 1.64E-07 | 8.99E-07 |
| MF | GO:0017110 | nucleoside-diphosphatase activity | 14/18337 | 2.64E-07 | 1.35E-06 |
| MF | GO:0051539 | 4 iron, 4 sulfur cluster binding | 42/18337 | 8.08E-07 | 3.88E-06 |
| MF | GO:0051536 | iron-sulfur cluster binding | 67/18337 | 8.46E-06 | 3.47E-05 |
| MF | GO:0051540 | metal cluster binding | 67/18337 | 8.46E-06 | 3.47E-05 |
| MF | GO:0003697 | single-stranded DNA binding | 116/18337 | 8.58E-06 | 3.47E-05 |
| MF | GO:0042578 | phosphoric ester hydrolase activity | 367/18337 | 2.04E-05 | 7.85E-05 |
| MF | GO:0004527 | exonuclease activity | 82/18337 | 2.28E-05 | 8.33E-05 |
| MF | GO:0008144 | drug binding | 61/18337 | 0.000118878 | 0.00041522 |
| MF | GO:0016791 | phosphatase activity | 276/18337 | 0.000146187 | 0.000488404 |
| MF | GO:0004518 | nuclease activity | 206/18337 | 0.000212468 | 0.000680271 |
| MF | GO:0000287 | magnesium ion binding | 216/18337 | 0.000274247 | 0.00084295 |
| MF | GO:0008296 | 3'-5'-exodeoxyribonuclease activity | 11/18337 | 0.000910094 | 0.002689751 |
| MF | GO:0016668 | oxidoreductase activity, acting on a sulfur group of donors, NAD(P) as acceptor | 12/18337 | 0.001089182 | 0.003099816 |
| MF | GO:0016763 | transferase activity, transferring pentosyl groups | 50/18337 | 0.001165805 | 0.003199389 |
| MF | GO:0008408 | 3'-5' exonuclease activity | 55/18337 | 0.001537473 | 0.004073886 |
| MF | GO:0016796 | exonuclease activity, active with either ribo- or deoxyribonucleic acids and producing 5'-phosphomonoesters | 57/18337 | 0.00170461 | 0.004260735 |
| MF | GO:0019215 | intermediate filament binding | 15/18337 | 0.001718885 | 0.004260735 |
| MF | GO:0004536 | deoxyribonuclease activity | 59/18337 | 0.001882693 | 0.00452094 |
| MF | GO:0004551 | nucleotide diphosphatase activity | 20/18337 | 0.003068929 | 0.00693597 |
| MF | GO:0050145 | nucleoside monophosphate kinase activity | 20/18337 | 0.003068929 | 0.00693597 |
| MF | GO:0015035 | protein disulfide oxidoreductase activity | 21/18337 | 0.003382897 | 0.007427111 |
| MF | GO:0004529 | exodeoxyribonuclease activity | 22/18337 | 0.003711231 | 0.007707534 |
| MF | GO:0016895 | exodeoxyribonuclease activity, producing 5'-phosphomonoesters | 22/18337 | 0.003711231 | 0.007707534 |
| MF | GO:0050660 | flavin adenine dinucleotide binding | 81/18337 | 0.004629775 | 0.009362148 |
| MF | GO:0003727 | single-stranded RNA binding | 88/18337 | 0.005832925 | 0.011492673 |
| MF | GO:0031369 | translation initiation factor binding | 32/18337 | 0.007758404 | 0.014904303 |
| MF | GO:0015036 | disulfide oxidoreductase activity | 40/18337 | 0.011943145 | 0.022383815 |
| MF | GO:0016879 | ligase activity, forming carbon-nitrogen bonds | 47/18337 | 0.016246518 | 0.029724206 |
| MF | GO:0016667 | oxidoreductase activity, acting on a sulfur group of donors | 58/18337 | 0.024128538 | 0.043118318 |
| MF | GO:0016627 | oxidoreductase activity, acting on the CH-CH group of donors | 59/18337 | 0.024909094 | 0.043501528 |

**Table 9b. KEGG enrichment analysis.**

| ID | Description | GeneRatio | BgRatio | pvalue | qvalue |
| --- | --- | --- | --- | --- | --- |
| hsa00240 | Pyrimidine metabolism | 36/76 | 58/8165 | 1.87E-62 | 1.96E-61 |
| hsa01232 | Nucleotide metabolism | 32/76 | 85/8165 | 9.37E-46 | 3.49E-45 |
| hsa03020 | RNA polymerase | 25/76 | 34/8165 | 9.95E-46 | 3.49E-45 |
| hsa04623 | Cytosolic DNA-sensing pathway | 16/76 | 63/8165 | 1.56E-19 | 4.10E-19 |
| hsa03030 | DNA replication | 12/76 | 36/8165 | 1.79E-16 | 3.78E-16 |
| hsa00983 | Drug metabolism - other enzymes | 15/76 | 80/8165 | 3.30E-16 | 5.79E-16 |
| hsa00230 | Purine metabolism | 17/76 | 128/8165 | 1.24E-15 | 1.87E-15 |
| hsa03410 | Base excision repair | 8/76 | 33/8165 | 4.45E-10 | 5.86E-10 |
| hsa03420 | Nucleotide excision repair | 8/76 | 47/8165 | 9.09E-09 | 1.06E-08 |
| hsa00760 | Nicotinate and nicotinamide metabolism | 6/76 | 36/8165 | 8.31E-07 | 8.75E-07 |
| hsa01240 | Biosynthesis of cofactors | 9/76 | 153/8165 | 1.10E-05 | 1.05E-05 |
| hsa03430 | Mismatch repair | 4/76 | 23/8165 | 5.37E-05 | 4.71E-05 |
| hsa05016 | Huntington disease | 11/76 | 306/8165 | 0.000111185 | 9.00E-05 |
| hsa03440 | Homologous recombination | 4/76 | 41/8165 | 0.000540757 | 0.000406585 |
| hsa00770 | Pantothenate and CoA biosynthesis | 3/76 | 21/8165 | 0.000913682 | 0.00064118 |
| hsa00450 | Selenocompound metabolism | 2/76 | 17/8165 | 0.010623114 | 0.006988891 |
| hsa00500 | Starch and sucrose metabolism | 2/76 | 36/8165 | 0.043939601 | 0.027207183 |

**Appendix 10**

**gene set enrichment analyses (GSEA)**

**Table 10a. GSEA of high rish.**

| NAME | SIZE | ES | NES | NOM p-val | FDR q-val |
| --- | --- | --- | --- | --- | --- |
| KEGG_NOD_LIKE_RECEPTOR_SIGNALING_PATHWAY | 62 | 0.6077263 | 2.025884 | 0 | 0.12448892 |
| KEGG_CYTOKINE_CYTOKINE_RECEPTOR_INTERACTION | 264 | 0.5610116 | 2.0137787 | 0.001886793 | 0.06888902 |
| KEGG_PATHOGENIC_ESCHERICHIA_COLI_INFECTION | 56 | 0.58388335 | 2.0131338 | 0 | 0.046198167 |
| KEGG_PROTEASOME | 46 | 0.7270414 | 1.9887205 | 0.006465518 | 0.047390986 |
| KEGG_PRION_DISEASES | 35 | 0.6320332 | 1.961903 | 0.003944773 | 0.048800196 |
| KEGG_OOCYTE_MEIOSIS | 113 | 0.5327654 | 1.938159 | 0.008048289 | 0.053109407 |
| KEGG_BLADDER_CANCER | 42 | 0.5463707 | 1.9359212 | 0 | 0.04700271 |
| KEGG_FOCAL_ADHESION | 199 | 0.5436419 | 1.933101 | 0.009578544 | 0.042265322 |
| KEGG_GLIOMA | 65 | 0.52664065 | 1.9174618 | 0.005736138 | 0.04451395 |
| KEGG_REGULATION_OF_ACTIN_CYTOSKELETON | 213 | 0.48842514 | 1.9056575 | 0.003968254 | 0.04555585 |
| KEGG_LEISHMANIA_INFECTION | 70 | 0.63311845 | 1.8969704 | 0.013108614 | 0.044920843 |
| KEGG_NATURAL_KILLER_CELL_MEDIATED_CYTOTOXICITY | 132 | 0.5288756 | 1.8775663 | 0.007751938 | 0.048347775 |
| KEGG_NUCLEOTIDE_EXCISION_REPAIR | 44 | 0.6271349 | 1.8559482 | 0.01863354 | 0.05190936 |
| KEGG_PATHWAYS_IN_CANCER | 325 | 0.445362 | 1.8418822 | 0.001964637 | 0.054202832 |
| KEGG_TOLL_LIKE_RECEPTOR_SIGNALING_PATHWAY | 102 | 0.50453025 | 1.8384811 | 0.013108614 | 0.05275685 |
| KEGG_PANCREATIC_CANCER | 70 | 0.49580687 | 1.8340912 | 0.005825243 | 0.05086163 |
| KEGG_HEMATOPOIETIC_CELL_LINEAGE | 85 | 0.6116926 | 1.8313062 | 0.01764706 | 0.048968576 |
| KEGG_CELL_CYCLE | 125 | 0.55770487 | 1.8286173 | 0.033264033 | 0.047536463 |
| KEGG_MELANOMA | 71 | 0.51984745 | 1.8233558 | 0.001855288 | 0.047086164 |
| KEGG_PYRIMIDINE_METABOLISM | 98 | 0.50120145 | 1.817545 | 0.016161617 | 0.04688096 |
| KEGG_DNA_REPLICATION | 36 | 0.7185649 | 1.8164389 | 0.014403292 | 0.04490206 |
| KEGG_CYTOSOLIC_DNA_SENSING_PATHWAY | 55 | 0.54433346 | 1.7924676 | 0.012 | 0.05276161 |
| KEGG_GAP_JUNCTION | 90 | 0.480992 | 1.790301 | 0.003824092 | 0.051308427 |
| KEGG_MISMATCH_REPAIR | 23 | 0.7068 | 1.785422 | 0.016194332 | 0.05157111 |
| KEGG_ARRHYTHMOGENIC_RIGHT_VENTRICULAR_CARDIOMYOPATHY_ARVC | 74 | 0.537912 | 1.7751106 | 0.009505703 | 0.0536109 |
| KEGG_PROGESTERONE_MEDIATED_OOCYTE_MATURATION | 85 | 0.4836293 | 1.7734414 | 0.013461539 | 0.05217413 |
| KEGG_ECM_RECEPTOR_INTERACTION | 84 | 0.5971549 | 1.7712816 | 0.015748031 | 0.050942607 |
| KEGG_CHEMOKINE_SIGNALING_PATHWAY | 188 | 0.48229632 | 1.770285 | 0.013461539 | 0.04941177 |
| KEGG_AUTOIMMUNE_THYROID_DISEASE | 50 | 0.64821774 | 1.7651685 | 0.031496063 | 0.049681056 |
| KEGG_GLYCOSAMINOGLYCAN_BIOSYNTHESIS_CHONDROITIN_SULFATE | 22 | 0.66389006 | 1.7594471 | 0.01775148 | 0.050259408 |
| KEGG_ANTIGEN_PROCESSING_AND_PRESENTATION | 81 | 0.59687704 | 1.7502446 | 0.034285713 | 0.051877696 |
| KEGG_PURINE_METABOLISM | 159 | 0.43084908 | 1.7463726 | 0.005905512 | 0.05161025 |
| KEGG_SMALL_CELL_LUNG_CANCER | 84 | 0.4868536 | 1.7459351 | 0.021653544 | 0.050210513 |
| KEGG_RIG_I_LIKE_RECEPTOR_SIGNALING_PATHWAY | 71 | 0.49393126 | 1.7452338 | 0.013779528 | 0.04895732 |
| KEGG_WNT_SIGNALING_PATHWAY | 151 | 0.43059656 | 1.7405038 | 0.003875969 | 0.04943049 |
| KEGG_MELANOGENESIS | 101 | 0.44513002 | 1.7348063 | 0.007648184 | 0.050218415 |
| KEGG_P53_SIGNALING_PATHWAY | 68 | 0.47439903 | 1.7331371 | 0.012371134 | 0.049593624 |
| KEGG_VIRAL_MYOCARDITIS | 68 | 0.5703715 | 1.7265943 | 0.028790787 | 0.05093822 |
| KEGG_AXON_GUIDANCE | 129 | 0.43649104 | 1.7242342 | 0.028037382 | 0.050440885 |
| KEGG_ALLOGRAFT_REJECTION | 35 | 0.7326922 | 1.7128558 | 0.044573642 | 0.05268823 |
| KEGG_RENAL_CELL_CARCINOMA | 70 | 0.4704144 | 1.7093835 | 0.019157087 | 0.05256611 |
| KEGG_ASTHMA | 28 | 0.66194093 | 1.7058128 | 0.05882353 | 0.052516825 |
| KEGG_NEUROTROPHIN_SIGNALING_PATHWAY | 126 | 0.43992242 | 1.6858478 | 0.011904762 | 0.058217295 |
| KEGG_GRAFT_VERSUS_HOST_DISEASE | 37 | 0.6980182 | 1.6572709 | 0.0625 | 0.068087265 |
| KEGG_DILATED_CARDIOMYOPATHY | 90 | 0.48239508 | 1.6387417 | 0.044036698 | 0.07503841 |
| KEGG_COMPLEMENT_AND_COAGULATION_CASCADES | 69 | 0.5317069 | 1.6271497 | 0.04509804 | 0.07991968 |
| KEGG_CHRONIC_MYELOID_LEUKEMIA | 73 | 0.44153613 | 1.6257334 | 0.021782178 | 0.07890105 |
| KEGG_TGF_BETA_SIGNALING_PATHWAY | 86 | 0.4326254 | 1.6113114 | 0.02457467 | 0.08391915 |
| KEGG_CELL_ADHESION_MOLECULES_CAMS | 131 | 0.48288283 | 1.608851 | 0.055658627 | 0.08351915 |
| KEGG_ACUTE_MYELOID_LEUKEMIA | 57 | 0.4567059 | 1.6056194 | 0.029126214 | 0.08324606 |
| KEGG_ADHERENS_JUNCTION | 73 | 0.43117362 | 1.5998102 | 0.046875 | 0.08450753 |
| KEGG_APOPTOSIS | 87 | 0.4336244 | 1.5938829 | 0.030947777 | 0.086436905 |
| KEGG_GALACTOSE_METABOLISM | 25 | 0.5187108 | 1.583655 | 0.03508772 | 0.09063769 |
| KEGG_JAK_STAT_SIGNALING_PATHWAY | 155 | 0.41279265 | 1.5645084 | 0.047348484 | 0.099386424 |
| KEGG_T_CELL_RECEPTOR_SIGNALING_PATHWAY | 108 | 0.44566447 | 1.561556 | 0.06641366 | 0.099200405 |
| KEGG_HYPERTROPHIC_CARDIOMYOPATHY_HCM | 83 | 0.47334412 | 1.5579754 | 0.062030077 | 0.09916506 |
| KEGG_MAPK_SIGNALING_PATHWAY | 267 | 0.37839895 | 1.5574282 | 0.02504817 | 0.09758726 |
| KEGG_GNRH_SIGNALING_PATHWAY | 101 | 0.37360904 | 1.5529859 | 0.015296367 | 0.09824511 |
| KEGG_TYPE_I_DIABETES_MELLITUS | 41 | 0.6073136 | 1.5521511 | 0.11389961 | 0.096930444 |
| KEGG_COLORECTAL_CANCER | 62 | 0.4274616 | 1.535127 | 0.04990403 | 0.1049048 |
| KEGG_EPITHELIAL_CELL_SIGNALING_IN_HELICOBACTER_PYLORI_INFECTION | 68 | 0.4204741 | 1.5316656 | 0.042145595 | 0.105116725 |
| KEGG_LONG_TERM_POTENTIATION | 70 | 0.39867404 | 1.4919995 | 0.0503876 | 0.12726773 |
| KEGG_ERBB_SIGNALING_PATHWAY | 87 | 0.3837234 | 1.4809659 | 0.06007752 | 0.13273717 |
| KEGG_FC_GAMMA_R_MEDIATED_PHAGOCYTOSIS | 96 | 0.37836477 | 1.4637841 | 0.079303674 | 0.14275654 |
| KEGG_GLYCOLYSIS_GLUCONEOGENESIS | 61 | 0.41755208 | 1.4534209 | 0.087576374 | 0.14787622 |
| KEGG_GLYOXYLATE_AND_DICARBOXYLATE_METABOLISM | 16 | 0.5648796 | 1.4459481 | 0.11506276 | 0.15119961 |
| KEGG_LEUKOCYTE_TRANSENDOTHELIAL_MIGRATION | 116 | 0.38166925 | 1.4016634 | 0.10404624 | 0.18369727 |
| KEGG_DRUG_METABOLISM_OTHER_ENZYMES | 51 | 0.39142475 | 1.3892184 | 0.08316008 | 0.19109686 |
| KEGG_SPHINGOLIPID_METABOLISM | 39 | 0.40723333 | 1.3798772 | 0.13157895 | 0.19572861 |
| KEGG_AMINOACYL_TRNA_BIOSYNTHESIS | 41 | 0.47253463 | 1.3668557 | 0.18619247 | 0.20395675 |
| KEGG_CALCIUM_SIGNALING_PATHWAY | 178 | 0.33885336 | 1.3605005 | 0.11666667 | 0.20629565 |
| KEGG_BASAL_CELL_CARCINOMA | 55 | 0.39794323 | 1.348046 | 0.11646587 | 0.21429162 |
| KEGG_SYSTEMIC_LUPUS_ERYTHEMATOSUS | 137 | 0.4386153 | 1.3414683 | 0.14889336 | 0.21771686 |
| KEGG_NON_SMALL_CELL_LUNG_CANCER | 54 | 0.3751291 | 1.341362 | 0.13001911 | 0.21486919 |
| KEGG_INTESTINAL_IMMUNE_NETWORK_FOR_IGA_PRODUCTION | 46 | 0.5200702 | 1.3411403 | 0.22070312 | 0.21215755 |
| KEGG_BASAL_TRANSCRIPTION_FACTORS | 35 | 0.4288765 | 1.3375521 | 0.17107943 | 0.21242636 |
| KEGG_NICOTINATE_AND_NICOTINAMIDE_METABOLISM | 24 | 0.41418844 | 1.3299129 | 0.121703856 | 0.21688895 |
| KEGG_GLYCOSAMINOGLYCAN_BIOSYNTHESIS_HEPARAN_SULFATE | 26 | 0.41115424 | 1.3200998 | 0.12698413 | 0.22232085 |
| KEGG_PENTOSE_PHOSPHATE_PATHWAY | 27 | 0.42964637 | 1.3179221 | 0.17038539 | 0.22171645 |
| KEGG_PROSTATE_CANCER | 89 | 0.35274377 | 1.3171761 | 0.14206642 | 0.21951672 |
| KEGG_HEDGEHOG_SIGNALING_PATHWAY | 56 | 0.35973972 | 1.3017626 | 0.15500945 | 0.23114865 |
| KEGG_VIBRIO_CHOLERAE_INFECTION | 54 | 0.37472025 | 1.2888708 | 0.1612284 | 0.239954 |
| KEGG_FRUCTOSE_AND_MANNOSE_METABOLISM | 34 | 0.39849544 | 1.2883677 | 0.184 | 0.23753631 |
| KEGG_ONE_CARBON_POOL_BY_FOLATE | 17 | 0.4805784 | 1.2783338 | 0.18292683 | 0.24440442 |
| KEGG_ENDOMETRIAL_CANCER | 52 | 0.3652331 | 1.2641293 | 0.1852552 | 0.25513163 |
| KEGG_TIGHT_JUNCTION | 131 | 0.31303182 | 1.2531307 | 0.15779468 | 0.26340982 |
| KEGG_SNARE_INTERACTIONS_IN_VESICULAR_TRANSPORT | 38 | 0.36918712 | 1.2522234 | 0.19760479 | 0.26122582 |
| KEGG_B_CELL_RECEPTOR_SIGNALING_PATHWAY | 75 | 0.3815475 | 1.2492322 | 0.24366471 | 0.26156136 |
| KEGG_NEUROACTIVE_LIGAND_RECEPTOR_INTERACTION | 272 | 0.3108974 | 1.2458428 | 0.13944954 | 0.26156798 |
| KEGG_LONG_TERM_DEPRESSION | 70 | 0.33224422 | 1.2432185 | 0.15354331 | 0.2612567 |
| KEGG_PRIMARY_IMMUNODEFICIENCY | 35 | 0.5013477 | 1.2422336 | 0.27667984 | 0.25946143 |
| KEGG_GLYCOSPHINGOLIPID_BIOSYNTHESIS_LACTO_AND_NEOLACTO_SERIES | 26 | 0.37668297 | 1.2250817 | 0.20467836 | 0.27314234 |
| KEGG_O_GLYCAN_BIOSYNTHESIS | 30 | 0.39938787 | 1.2051681 | 0.22393823 | 0.29111338 |
| KEGG_CYSTEINE_AND_METHIONINE_METABOLISM | 34 | 0.370829 | 1.2039723 | 0.24390244 | 0.2891482 |
| KEGG_THYROID_CANCER | 29 | 0.36780643 | 1.1904366 | 0.2296748 | 0.30031928 |
| KEGG_BASE_EXCISION_REPAIR | 35 | 0.39521715 | 1.1783944 | 0.29482073 | 0.30914474 |
| KEGG_RIBOFLAVIN_METABOLISM | 16 | 0.4283145 | 1.1767889 | 0.2631579 | 0.3078368 |
| KEGG_RNA_POLYMERASE | 29 | 0.3901762 | 1.1638972 | 0.27125505 | 0.31870794 |
| KEGG_HOMOLOGOUS_RECOMBINATION | 28 | 0.41677198 | 1.1602308 | 0.32409382 | 0.3197919 |
| KEGG_ARGININE_AND_PROLINE_METABOLISM | 54 | 0.3235145 | 1.1590228 | 0.2601626 | 0.31777537 |
| KEGG_GLYCOSAMINOGLYCAN_DEGRADATION | 21 | 0.406653 | 1.158863 | 0.2688766 | 0.31489775 |
| KEGG_DORSO_VENTRAL_AXIS_FORMATION | 24 | 0.36955458 | 1.1528692 | 0.294 | 0.31835374 |
| KEGG_UBIQUITIN_MEDIATED_PROTEOLYSIS | 135 | 0.30179042 | 1.1501591 | 0.27125505 | 0.31804234 |
| KEGG_INSULIN_SIGNALING_PATHWAY | 136 | 0.2768677 | 1.1445595 | 0.2820037 | 0.32118997 |
| KEGG_VASCULAR_SMOOTH_MUSCLE_CONTRACTION | 115 | 0.30301166 | 1.1389184 | 0.2932331 | 0.32407594 |
| KEGG_PYRUVATE_METABOLISM | 40 | 0.34942806 | 1.1388419 | 0.3049505 | 0.32109177 |
| KEGG_AMYOTROPHIC_LATERAL_SCLEROSIS_ALS | 53 | 0.3118413 | 1.1380508 | 0.2651822 | 0.31900617 |
| KEGG_LYSINE_DEGRADATION | 44 | 0.34588692 | 1.1344844 | 0.30343512 | 0.32012045 |
| KEGG_ADIPOCYTOKINE_SIGNALING_PATHWAY | 66 | 0.303322 | 1.108724 | 0.30994153 | 0.34510168 |
| KEGG_STARCH_AND_SUCROSE_METABOLISM | 51 | 0.30831805 | 1.0956322 | 0.31707317 | 0.35685575 |
| KEGG_GLYCOSAMINOGLYCAN_BIOSYNTHESIS_KERATAN_SULFATE | 15 | 0.38928875 | 1.0870975 | 0.36872587 | 0.36297533 |
| KEGG_RNA_DEGRADATION | 59 | 0.3240216 | 1.063087 | 0.37575758 | 0.3879796 |
| KEGG_AMINO_SUGAR_AND_NUCLEOTIDE_SUGAR_METABOLISM | 44 | 0.32868052 | 1.0620961 | 0.4019802 | 0.3856137 |
| KEGG_LYSOSOME | 121 | 0.31093067 | 1.0604715 | 0.39089185 | 0.3842209 |
| KEGG_CITRATE_CYCLE_TCA_CYCLE | 31 | 0.3996721 | 1.0583606 | 0.41617358 | 0.38339365 |
| KEGG_PANTOTHENATE_AND_COA_BIOSYNTHESIS | 16 | 0.36289096 | 1.0503085 | 0.3972056 | 0.3894468 |
| KEGG_STEROID_BIOSYNTHESIS | 17 | 0.39658046 | 1.0068469 | 0.43524417 | 0.43993247 |
| KEGG_NOTCH_SIGNALING_PATHWAY | 47 | 0.2771518 | 1.0004827 | 0.43897638 | 0.44478214 |
| KEGG_PROTEIN_EXPORT | 24 | 0.33831125 | 0.98687845 | 0.46138212 | 0.45776924 |
| KEGG_BIOSYNTHESIS_OF_UNSATURATED_FATTY_ACIDS | 22 | 0.34131566 | 0.97820127 | 0.4828283 | 0.46485266 |
| KEGG_TRYPTOPHAN_METABOLISM | 40 | 0.29105675 | 0.9648937 | 0.4969325 | 0.47926524 |
| KEGG_SPLICEOSOME | 127 | 0.29484436 | 0.9210599 | 0.49898168 | 0.5356265 |
| KEGG_REGULATION_OF_AUTOPHAGY | 35 | 0.28022727 | 0.90906733 | 0.5795678 | 0.54753464 |
| KEGG_N_GLYCAN_BIOSYNTHESIS | 46 | 0.2860665 | 0.9067025 | 0.5532787 | 0.54651016 |
| KEGG_PORPHYRIN_AND_CHLOROPHYLL_METABOLISM | 41 | 0.26232836 | 0.88075113 | 0.64271456 | 0.5782681 |
| KEGG_OLFACTORY_TRANSDUCTION | 387 | 0.2543891 | 0.86319834 | 0.6787879 | 0.5999108 |
| KEGG_GLYCOSPHINGOLIPID_BIOSYNTHESIS_GANGLIO_SERIES | 15 | 0.29953045 | 0.8231711 | 0.6921606 | 0.65337396 |
| KEGG_NITROGEN_METABOLISM | 23 | 0.25929785 | 0.81716925 | 0.73964494 | 0.65623236 |
| KEGG_RENIN_ANGIOTENSIN_SYSTEM | 17 | 0.29640657 | 0.79225785 | 0.7356322 | 0.6880025 |

**Table 10b. GSEA of low rish.**

| NAME | SIZE | ES | NES | NOM p-val | FDR q-val |
| --- | --- | --- | --- | --- | --- |
| KEGG_ALPHA_LINOLENIC_ACID_METABOLISM | 19 | -0.7724566 | -2.2641535 | 0 | 3.60E-04 |
| KEGG_VALINE_LEUCINE_AND_ISOLEUCINE_DEGRADATION | 44 | -0.66707414 | -2.1426907 | 0 | 0.002941816 |
| KEGG_LINOLEIC_ACID_METABOLISM | 29 | -0.6642279 | -2.0364537 | 0 | 0.012096318 |
| KEGG_RIBOSOME | 88 | -0.6408556 | -1.4185188 | 0.19111969 | 0.17647195 |
| KEGG_FATTY_ACID_METABOLISM | 42 | -0.6314287 | -2.1439023 | 0.00212766 | 0.004412724 |
| KEGG_ETHER_LIPID_METABOLISM | 33 | -0.5734103 | -1.9606378 | 0.002083333 | 0.014773856 |
| KEGG_PEROXISOME | 78 | -0.5624614 | -2.0302668 | 0.001972387 | 0.01061591 |
| KEGG_OXIDATIVE_PHOSPHORYLATION | 132 | -0.55205446 | -1.723917 | 0.058380414 | 0.06570958 |
| KEGG_BUTANOATE_METABOLISM | 34 | -0.53355545 | -1.7822242 | 0.00814664 | 0.05257732 |
| KEGG_PARKINSONS_DISEASE | 130 | -0.52621835 | -1.6839135 | 0.05436893 | 0.07815701 |
| KEGG_CARDIAC_MUSCLE_CONTRACTION | 79 | -0.51155704 | -1.7675903 | 0.022357723 | 0.053031974 |
| KEGG_DRUG_METABOLISM_CYTOCHROME_P450 | 71 | -0.50570697 | -1.7856321 | 0.005882353 | 0.06409178 |
| KEGG_GLYCEROPHOSPHOLIPID_METABOLISM | 76 | -0.5015605 | -1.9999448 | 0 | 0.011837669 |
| KEGG_ARACHIDONIC_ACID_METABOLISM | 58 | -0.4961863 | -1.7840674 | 0.004192872 | 0.057515804 |
| KEGG_PENTOSE_AND_GLUCURONATE_INTERCONVERSIONS | 28 | -0.48998585 | -1.496649 | 0.056112226 | 0.14581285 |
| KEGG_METABOLISM_OF_XENOBIOTICS_BY_CYTOCHROME_P450 | 69 | -0.4862542 | -1.6403867 | 0.02534113 | 0.08158909 |
| KEGG_OTHER_GLYCAN_DEGRADATION | 16 | -0.4776123 | -1.2149825 | 0.26477024 | 0.2863338 |
| KEGG_PPAR_SIGNALING_PATHWAY | 69 | -0.4688164 | -1.6828393 | 0.01632653 | 0.07280773 |
| KEGG_PROPANOATE_METABOLISM | 33 | -0.45939893 | -1.4293534 | 0.11924686 | 0.17503393 |
| KEGG_SELENOAMINO_ACID_METABOLISM | 26 | -0.45680594 | -1.432873 | 0.11546392 | 0.18014088 |
| KEGG_TYROSINE_METABOLISM | 42 | -0.45141712 | -1.5747926 | 0.032258064 | 0.11352614 |
| KEGG_GLYCINE_SERINE_AND_THREONINE_METABOLISM | 31 | -0.450863 | -1.4636501 | 0.06613226 | 0.16196029 |
| KEGG_PROXIMAL_TUBULE_BICARBONATE_RECLAMATION | 23 | -0.44621778 | -1.336718 | 0.12582782 | 0.20348068 |
| KEGG_RETINOL_METABOLISM | 64 | -0.43790153 | -1.5606856 | 0.025540275 | 0.11636631 |
| KEGG_GLYCEROLIPID_METABOLISM | 49 | -0.42845717 | -1.653485 | 0.018072288 | 0.08108828 |
| KEGG_ALANINE_ASPARTATE_AND_GLUTAMATE_METABOLISM | 32 | -0.42208728 | -1.38698 | 0.09475806 | 0.18773562 |
| KEGG_ASCORBATE_AND_ALDARATE_METABOLISM | 25 | -0.41762832 | -1.2136229 | 0.22920893 | 0.27948225 |
| KEGG_GLYCOSYLPHOSPHATIDYLINOSITOL_GPI_ANCHOR_BIOSYNTHESIS | 25 | -0.41703498 | -1.1838105 | 0.26403326 | 0.27274382 |
| KEGG_HUNTINGTONS_DISEASE | 182 | -0.4115112 | -1.5226479 | 0.083809525 | 0.13450114 |
| KEGG_TERPENOID_BACKBONE_BIOSYNTHESIS | 15 | -0.4029212 | -1.0124055 | 0.45918366 | 0.42370778 |
| KEGG_PRIMARY_BILE_ACID_BIOSYNTHESIS | 16 | -0.401756 | -1.1124774 | 0.34607646 | 0.3330911 |
| KEGG_HISTIDINE_METABOLISM | 29 | -0.3967088 | -1.3088666 | 0.14979757 | 0.21990915 |
| KEGG_VASOPRESSIN_REGULATED_WATER_REABSORPTION | 44 | -0.38675094 | -1.3559902 | 0.12017167 | 0.20090741 |
| KEGG_BETA_ALANINE_METABOLISM | 22 | -0.38009048 | -1.1856947 | 0.23958333 | 0.27749285 |
| KEGG_GLUTATHIONE_METABOLISM | 49 | -0.37985364 | -1.2834972 | 0.17366412 | 0.23464994 |
| KEGG_ALZHEIMERS_DISEASE | 166 | -0.37305278 | -1.391885 | 0.1436673 | 0.1910049 |
| KEGG_INOSITOL_PHOSPHATE_METABOLISM | 54 | -0.3648621 | -1.3422238 | 0.13135593 | 0.205411 |
| KEGG_VEGF_SIGNALING_PATHWAY | 76 | -0.34040394 | -1.3676922 | 0.091295116 | 0.19745757 |
| KEGG_TYPE_II_DIABETES_MELLITUS | 47 | -0.3336063 | -1.2042615 | 0.19456068 | 0.27372116 |
| KEGG_MATURITY_ONSET_DIABETES_OF_THE_YOUNG | 25 | -0.33300647 | -0.86307603 | 0.60554373 | 0.5810331 |
| KEGG_PHENYLALANINE_METABOLISM | 18 | -0.33071148 | -0.99320745 | 0.43505156 | 0.4378684 |
| KEGG_MTOR_SIGNALING_PATHWAY | 52 | -0.3129585 | -1.1938199 | 0.2112069 | 0.2765661 |
| KEGG_PHOSPHATIDYLINOSITOL_SIGNALING_SYSTEM | 76 | -0.31182405 | -1.2065353 | 0.22222222 | 0.27910683 |
| KEGG_ALDOSTERONE_REGULATED_SODIUM_REABSORPTION | 42 | -0.30773732 | -1.0844843 | 0.33544305 | 0.35517937 |
| KEGG_ENDOCYTOSIS | 181 | -0.30134067 | -1.2634851 | 0.14613779 | 0.24551149 |
| KEGG_FC_EPSILON_RI_SIGNALING_PATHWAY | 79 | -0.29817772 | -1.15981 | 0.26190478 | 0.29052922 |
| KEGG_STEROID_HORMONE_BIOSYNTHESIS | 55 | -0.29527783 | -1.0639379 | 0.35452795 | 0.37078577 |
| KEGG_TASTE_TRANSDUCTION | 51 | -0.28670895 | -0.88802075 | 0.607362 | 0.5569907 |
| KEGG_ABC_TRANSPORTERS | 44 | -0.27090374 | -0.98747313 | 0.46107784 | 0.43587098 |
